# Interactomic exploration of LRRC8A in volume-regulated anion channels

**DOI:** 10.1038/s41420-024-02032-0

**Published:** 2024-06-22

**Authors:** Veronica Carpanese, Margherita Festa, Elena Prosdocimi, Magdalena Bachmann, Soha Sadeghi, Sara Bertelli, Frank Stein, Angelo Velle, Mostafa A. L. Abdel-Salam, Chiara Romualdi, Michael Pusch, Vanessa Checchetto

**Affiliations:** 1 DiBio, Unipd, via Ugo Bassi 58/B, 35131 Padova, Italy; 2 Institute of Biophysics, CNR, Via De Marini, 6, 16149 Genova, Italy; 3 Proteomics Core Facility, EMBL Heidelberg, Meyerhofstraße 1, 69117 Heidelberg, Germany; 4 Padua Center for Network Medicine, University of Padua, Via F. Marzolo 8, 315126 Padova, Italy; 5 RAISE Ecosystem, Genova, Italy; 6 Present Address: Institute of Biophysics, CNR, Via De Marini, 6 16149, Genova, Italy; 7 Present Address: Daba Farber Cancer Research Institute, Boston, MA USA; 8 Present Address: Humboldt Universität Berlin, AG Zelluläre Biophysik, Dorotheenstr, 19-21 10099 Berlin, Germany; 9 Present Address: UPMC Hillman Cancer Center, Pittsburgh, PA USA

**Keywords:** Chloride channels, Protein-protein interaction networks

## Abstract

Ion channels are critical in enabling ion movement into and within cells and are important targets for pharmacological interventions in different human diseases. In addition to their ion transport abilities, ion channels interact with signalling and scaffolding proteins, which affects their function, cellular positioning, and links to intracellular signalling pathways. The study of “channelosomes” within cells has the potential to uncover their involvement in human diseases, although this field of research is still emerging. *LRRC8A* is the gene that encodes a crucial protein involved in the formation of volume-regulated anion channels (VRACs). Some studies suggest that LRRC8A could be a valuable prognostic tool in different types of cancer, serving as a biomarker for predicting patients’ outcomes. LRRC8A expression levels might be linked to tumour progression, metastasis, and treatment response, although its implications in different cancer types can be varied. Here, publicly accessible databases of cancer patients were systematically analysed to determine if a correlation between VRAC channel expression and survival rate exists across distinct cancer types. Moreover, we re-evaluated the impact of LRRC8A on cellular proliferation and migration in colon cancer via HCT116 LRRC8A-KO cells, which is a current topic of debate in the literature. In addition, to investigate the role of LRRC8A in cellular signalling, we conducted biotin proximity-dependent identification (BioID) analysis, revealing a correlation between VRAC channels and cell-cell junctions, mechanisms that govern cellular calcium homeostasis, kinases, and GTPase signalling. Overall, this dataset improves our understanding of LRRC8A/VRAC and explores new research avenues while identifying promising therapeutic targets and promoting inventive methods for disease treatment.

## Introduction

Ion channels mediate the movement of ions across cellular membranes and play important roles in the development and progression of several human diseases [1–4]. Channel proteins are complex structures consisting of a central ion-selective pore and various interacting proteins. Channel-interacting proteins (CIPs) can play a critical role in regulating biophysical properties, such as permeability and gating, or act as signalling and scaffolding proteins that influence the interaction of ion channels with upstream and downstream cellular signalling pathways [1–3, 5, 6].

The increasing knowledge of ion channels as macromolecular signalling complexes is transforming research methods in this field. Although high-throughput gene profiling techniques are commonly employed, studying the ion channel interactome, or channelosome, can reveal interconnected networks that affect not only ion channel activity but also complex signalling pathways. This approach is crucial in identifying groups of molecules that could act as targets for interventions aiming to manage or ameliorate pathological conditions. Additionally, it is essential to acknowledge that network-based approaches provide a more nuanced comprehension of the complex pathways involved in disease.

The identification of protein-protein interactions (PPIs) within a cellular context presents significant challenges, as conventional methods often prove ineffective in a natural cellular environment. Classical approaches may fail to ensure the detection of interactions involving weak or transient interactors, as well as those subject to spatio-temporal regulation. These limitations result in a substantial loss of valuable information, stemming from an incomplete understanding of the dynamic nature of these interactions. Initially, research in CIPs field mainly focused on cation channels, particularly those selectively permeable to calcium, potassium, and sodium ions. Nevertheless, in recent years, there has been a growing interest in anion channels, i.e., chloride (Cl) channels. These channels play a vital role in various cellular processes and their abnormal expression and/or function is associated with various human diseases, such as cystic fibrosis, myotonia, epilepsy, hyperekplexia, lysosomal storage diseases, deafness, renal salt wasting, kidney stones, osteopetrosis and numerous tumour development of numerous types of tumours [7–9].

Among anion channels, the volume-regulated anion channel (VRAC) is emerging as a promising pharmacological target in human pathology and oncology. VRAC has a broad permeability, enabling the transfer of Cl^-^ and other anions, organic compounds, neurotransmitters, taurine, and signalling molecules [10–15]. VRAC plays a critical role in regulating cell volume by reducing it through a process called regulatory volume decrease (RVD) and maintaining cell volume homeostasis. Due to its role in RVD, VRAC has been proposed to be involved in cell volume changes during different aspects of cancer cell behaviour and response to therapies. The activity of VRAC has been linked to cancer cell proliferation, metastasis, and multidrug resistance [11, 16]. Nonetheless, the biological function and prognostic value of the gene encoding the pore-forming subunit of VRAC, LRRC8, require better delineation, as contrasting results have been reported.

In this work, we examined the publicly accessible TCGA database to comprehensively analyse the relationship between LRRC8 expression in cancer and patient survival. We focused specifically on the colon cancer context.

Despite the functional characterization of VRAC has been known for several decades, its molecular identity has been unveiled only recently [17, 18]. These investigations shed light on VRAC as a heteromeric assembly that consists of subunits belonging to the LRRC8 gene family, comprising of five distinct members (LRRC8A-E) [17, 18]. Evolutionary, LRRC8 proteins are formed by combining a pannexin-like transmembrane protein and an intracellular leucine-rich repeat domain (LRRD) [19]. LRRC8 proteins are made up of four transmembrane segments and a C-terminal leucine-rich repeat domain. The N-terminal domain was recently found to fold back into the pore from the cytoplasm, taking part in determining ion selectivity and possibly gating [20]. The precise proportions and arrangement of subunits needed to create functional LRRC8 heteromers have not yet been fully discovered. To operate efficiently, VRAC necessitates the presence of LRRC8A and at least one subunit among the LRRC8B-E isoforms [17, 18]. Recently, in cryoEM studies of heteromeric LRRC8A/LRRC8C complexes, different stoichiometries have been reported: Rutz et al. found 4 LRRC8A subunits and 2 LRRC8C subunits in heteromers [21], while Kern et al. reported a 5 LRRC8A/1 LRRC8C architecture [22]. In general, most cells express more than two different LRRC8 genes and LRRC8A-E assemble in various configurations, leading to the formation of VRACs with differing functional characteristics. At present, we have limited knowledge of the identities of the subunits that compose the VRAC pore, as well as how the channel is activated by cellular swelling.

Over the past three decades, intensive research endeavours have unveiled an extensive network of potential PPIs that exert a pivotal role in modulating the activity of VRAC. Experimental evidence indicates that the activation of VRAC currents can be achieved through purinergic signalling [23], a process involving calcium (Ca^2+^) signalling and protein phosphorylation events [24], as well as by the activation of bradykinin receptor signalling, which is intricately regulated by reactive oxygen species (ROS) and Ca^2+^ nanodomains [23, 25]. Furthermore, ROS have been suggested to influence the activation of VRAC by EGF [26]. Additionally, VRAC can be induced isovolumetrically by intracellular GTPγS [27, 28]. Volume-independent activation of VRAC is also induced by sphingosine-1-phosphate, which is generated by bacterial lipopolysaccharide-activated S-kinase, PDGF, TNFα, thrombin, IgE-bound antigen, and especially ATP [29].

Despite considerable effort, there is an insufficient amount of information available on its signalling networks. In 2016, Syeda et al. generated a cell line expressing LRRC8A with a FLAG-tag to biochemically investigate the protein and its associated partners [30]. The detection of an 800-kDa complex in native gels implies potential interactions between LRRC8 subunits and other proteins. The authors used mass spectrometry (MS) to identify the proteins associated with LRRC8A. Only peptides from the four LRRC8 family members were detected, and no other binding partners were found. Probably, the lack of data on VRAC interaction factors in the literature owes to the channel’s hydrophobicity and the technical challenges associated with biochemical manipulation. Indeed, in the authors themselves, suggest that the use of detergents and the tag affinity purification process may have resulted in the loss of probable associations [30].

In the present investigation, we have undertaken an in-depth exploration of the PPI network associated with LRRC8A. This endeavour was accomplished by the application of the state-of-the art BioID technique, with a specific emphasis on the central subunit LRRC8A. We exploited the BioID methodology to construct an exhaustive compendium of proteins engaged in interactions with LRRC8A/VRAC. These interactions encompass a spectrum of strengths, temporal dynamics, and indirect connections. The discerned proteins are presumed to establish close or direct functional affiliations with the LRRC8A subunit, thereby harbouring the potential to bestow valuable insights into the pathophysiological mechanisms and underlying functional roles.

## Results

### LRRC8s alterations and expression in human cancers: impact on patient survival

**Fig. 1:**
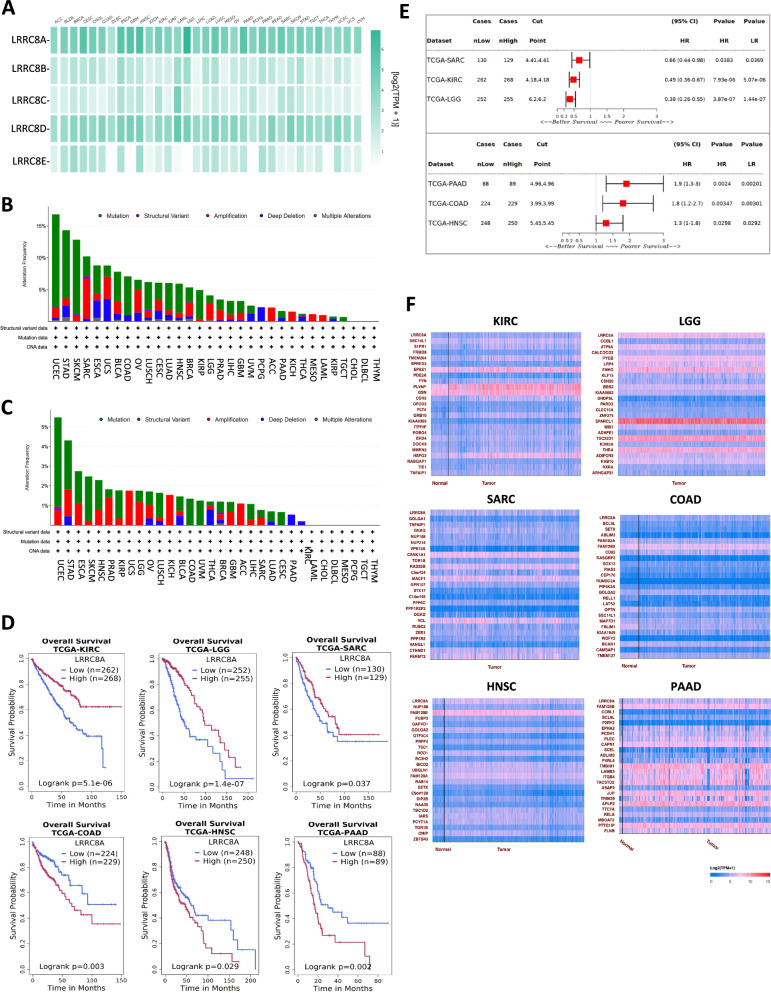
LRRC8s family in human cancers. **A** Differential expression of LRRC8s family in human cancers. LRRC8s multiple gene comparison was performed using TCGA and GTEx datasets and using the GEPIA database. Data were normalized as transcripts per kilobase million (TPM) values. TPM values were converted to log2-normalized transcripts per million [log2(TPM + 1)]. **B** Mutation frequencies of LRRC8s in 32 cancer studies were retrieved from cBioPortal (TCGA Pan-Cancer Atlas dataset). **C** Mutation frequencies of LRRC8A in 32 cancer studies were retrieved from cBioPortal (TCGA Pan-Cancer Atlas dataset). **D** Survival plots based on LRRC8A expression level in represented tumours were obtained through Kaplan–Meier analysis by sorting samples for high and low LRRC8A expression groups according to Survival Genie software. **E** The forest plot illustrates hazard ratio (HR) analyses. The results of the Wald test (HR P-value) and log-rank test (LR P-value) are also displayed. **F** LRRC8A correlated differentially expressed genes and related pathways. The top 25 positively LRRC8A co-expressed genes were mapped using the TCGA KIRC, LGG, SARC, COAD, HNSC, and PAAD datasets in the ULCAN database.

The GEPIA database [31] was used to evaluate the expression profile of LRRC8 genes. A comparative analysis of multiple genes revealed that LRRC8A and LRRC8D exhibit higher expression in tumours when compared to other LRRC8 genes, providing an overall characterization (Fig. 1A). Exploring the publicly accessible TCGA database enabled us to systematically investigate the correlation between VRAC channels in specific cancer types and patient survival. To assess comprehensive alterations, we analysed all LRRC8 genes (LRRC8A-E) using the TCGA Pan-Cancer Atlas dataset, combining data from 32 human cancers, encompassing a total of 10,953 patients. Genetic alterations within the cBioPortal database were categorized as mutations, deep deletions, gene amplifications, structural variants, and multiple alterations [32, 33]. The graphical representation depicting cancer types illustrates a discernible pattern of genomic alterations in LRRC8 genes across the TCGA PanCancer cohorts (Fig. 1B). The results indicate that the 10 types of cancer with the highest frequency of alterations were uterine corpus endometrial carcinoma (UCEC), stomach adenocarcinoma (STAD), skin cutaneous melanoma (SKCM), sarcoma (SARC) and oesophageal adenocarcinoma (ESCA), uterine carcinosarcoma (UCS), bladder urothelial carcinoma (BLCA), colorectal adenocarcinoma (COAD), ovarian serous cystadenocarcinoma (OV), and lung squamous cell carcinoma (LUSC). The cumulative mutation frequency ranged from 16.82% for UCEC to 6.16% for LUSC. Truncating, in-frame, or missense mutations were prevalent in LRRC8 genes, while amplifications, multiple alterations, or deep deletions, especially homozygous deletions in non-aneuploidy cases, were less frequently observed. Figure 1C delineates the specific genetic alterations of the LRRC8A gene.

To highlight the prognostic impact of LRRC8A expression in patients with cancer, we evaluated the Kaplan-Meier analysis to classify samples into groups with high and low LRRC8A expression. Our findings indicate a significant association between LRRC8A expression levels and patient prognosis across six different types of cancer. Patients with high LRRC8A levels had a significantly worse prognosis in cases of COAD, Head-Neck Squamous Cell Carcinoma (HNSC), and Pancreatic adenocarcinoma (PAAD). Conversely, Kidney renal clear cell carcinoma (KIRC), Low-grade glioma (LGG), and SARC demonstrated a favourable outcome (Fig. 1D). However, statistically significant results were not obtained for other types of tumours. The forest plot (Fig. 1E) illustrating hazard ratio (HR) analyses, was generated using the Survival Genie software, a web-based platform designed for conducting survival analyses in both paediatric and adult cancer populations [16]. This graph illustrates the HR and corresponding 95% confidence intervals for two distinct groups analysed univariably. The results of the Wald test (HR P-value) and log-rank test (LR P-value) are also displayed. Important details, such as the cut-off point used to classify patients as having high or low expression, and the sample sizes for each group, are provided. In the figure, the hazard ratio is represented by a central box (significant correlations are highlighted in red), and the lower and upper bounds of the 95% confidence interval are indicated by horizontal lines. For example, for COAD, the patients were divided into two groups, namely high (*n* = 229 samples) and low (*n* = 224 samples). It has been established those patients in the high group, with a median cut-off point of 3.99, exhibit an unfortunate prognosis (HR = 1.8; 95% CI 1.2-2.7; *P* = 0.00347 by Wald-test, and *P* = 0.0030 by log-rank test) (Fig. 1E).

Fascinated by the dual role of LRRC8A/VRAC in tumours, which is linked to both favourable and unfavourable prognoses (as illustrated in Fig. 1D and E), our aim was to recognize differentially expressed genes (DEGs) related to LRRC8A to illuminate its functional roles (Fig. 1F). To achieve this objective, a thorough examination of genes with a positive correlation to LRRC8A in KIRC, LGG, SARC, COAD, HNSC, and PAAD tumour types was conducted in their respective TCGA datasets. This was accomplished by utilizing the UALCAN database [34, 35]. Over 100 differentially DEGs were found to be shared between tumour types associated with both a positive prognosis (KIRC, LGG, and SARC) and those linked to a negative prognosis (COAD, HNSC and PAAD,) within the TCGA tumours datasets. This suggests that these common genes may play a critical role in tumour progression and outcome. The levels of LRRC8A transcripts discovered in different tumours and the difference in LRRC8A expression between normal and tumours tissue lack justification for the impact of genes on survival, suggesting that aspects beyond gene expression need to be studied.

**Fig. 2:**
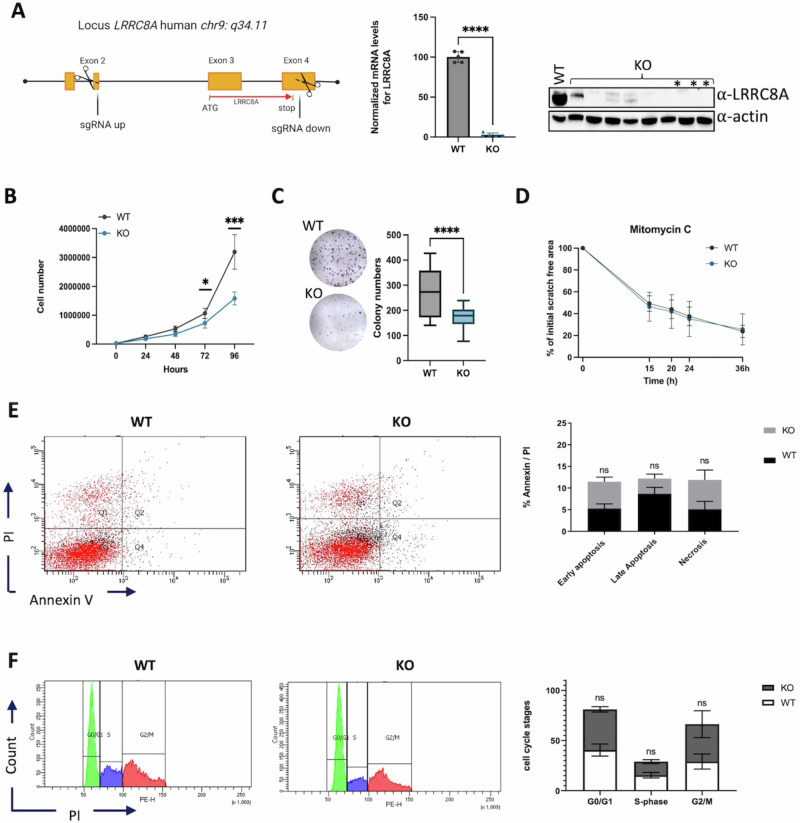
Characterization of LRRC8A knockout HCT116 cells. **A** Strategy of CRISPR/Cas9 editing applied for LRRC8A and characterization of LRRC8A KO HCT116 cells. KO-LRRC8A monoclonal HCTT16 cell lines were isolated and evaluated for the expression of their LRRC8A gene using real-time qPCR. HCT116 WT cells were used as a control, and the housekeeping genes *ACTB* and *TBP* were employed. Gene expression data were normalised to the control and presented as a percentage variation (*n* = 5, one-way ANOVA, ****p < 0.0001). In addition, the α-LRRC8A antibody was used to assess the protein expression of LRRC8A, with HCT116 WT cells as the control. Protein extracts (50 μg) were loaded for each sample, with actin serving as the loading control. Single clones indicated by a red asterisk were selected for further analysis. **B** Growth curves of KO- and WT-LRRC8A HCT116 cells. At the 96-hour, it was found that KO clones exhibited a lower proliferation rate compared to HCT116 WT control (*n* = 4, two-way ANOVA, ***p-value < 0.001). **C** Colony formation assay. LRRC8A-KO cells formed significantly fewer colonies compared to HCT116 WT control (*n* = 5, one-way ANOVA, *p-value < 0.5). **D** The Wound Healing Assay examined migratory capacity by measuring the percentage of the initial scratch area free at various time points (from *t* = 0 h to *t* = 36 h) using ImageJ software. In both experimental settings (mitomycin 5 μg/mL), HCT116 WT control and LRRC8A-KO clones displayed no significant differences in scratch closure speed (*n* = 4, two-way ANOVA). Illustrative histograms showing HCT116 WT and KO clone. Statistical analysis of independent triplicate experiments showed non-significant differences between WT and the KO cells in terms of apoptosis (**E**) and cell cycle (**F**). Data were compared by Nonparametric T- test with a significance level of *p* < 0.05.

Our in-depth analysis, as illustrated in Fig. 2 supports the above findings and specifically examines the effects of LRRC8A deletion on HCT116 cell behaviour. The increased expression of LRRC8A seems to be linked with reduced survival among CRC patients with positive lymph nodes, indicating LRRC8A proteins’ possible involvement in CRC metastasis by aiding cell migration [36]. However, these findings contradict those reported by Liu et al. [37], who showed that the deletion of LRRC8A or all members of LRRC8 did not reduce the migration of HCT116 cells. To gain new insights, we employed CRISPR/Cas9 technology to generate a new LRRC8A-deficient HCT116 knockout cell line. Our model completely removed the LRRC8A gene, and we isolated two independent monoclonal cell lines for further analysis. Target site-specific PCR was utilized to evaluate any modifications in the genomic DNA sequence. The lack of LRRC8A expression was verified by Sanger sequencing, Western blot, and RT-qPCR (Fig. 2A). To investigate the potential effects of LRRC8A, cell proliferation and wound healing assays were performed on HCT116 cells that either expressed or lacked LRRC8A (Fig. 2B–D). Cell counting over a period of up to 96 hours demonstrated a significant decrease in proliferation rate for the KO clones in comparison to the WT controls at both 72 hours (*p* = 0.0130) and 96 hours (*p* < 0.0001), suggesting an involvement of LRRC8A in cell proliferation (Fig. 2B). To further explore the influence of LRRC8A on cell proliferation further, a colony formation assay was conducted. The data demonstrated a substantial reduction (*p* < 0.0001) in colony count for the KO clones relative to the controls, consistent with the outcomes of the cell proliferation assay (Fig. 2C). Finally, we explored the function of LRRC8A in cell migration via a wound healing assay. Supplementary treatment of cells with mitomycin C validated that the formation of a confluent cell monolayer post-scratching was attributed to migration proficiency rather than cellular proliferation. No significant differences were found in the migration rate between the controls and KO clones during both experimental conditions (Fig. 2D).

Numerous studies have suggested that LRRC8A supports cell survival under hypotonic conditions and facilitates tumorigenesis by suppressing apoptosis both in vitro and in vivo [38, 39]. Consequently, we investigated the influence of LRRC8A on apoptosis in HCT116 WT and KO cells using an Annexin V/propidium iodide assay. However, our results revealed no discernible impact on apoptosis events with the deletion of LRRC8A (Fig. 2E). Additionally, the analysis of the cell cycle did not reveal any significant differences in the G0/G1, S, and G2/M phases (Fig. 2F).

### Identification and Analysis of the LRRC8A Differentially Expressed Genes (DEGs) in colon Cancer

**Fig. 3:**
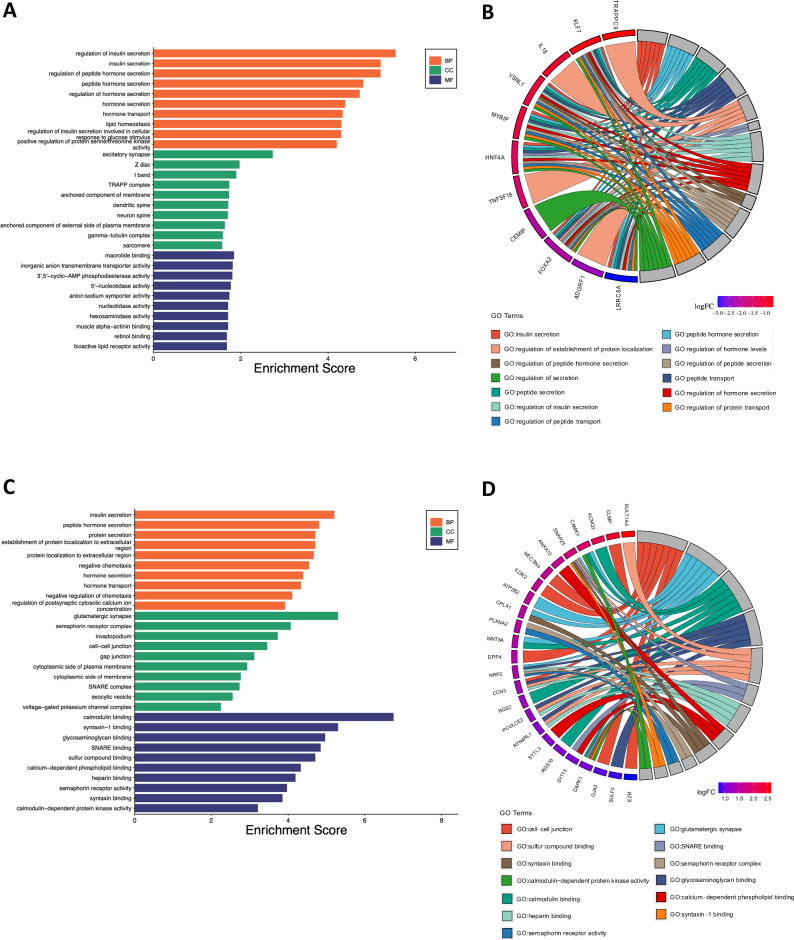
Gene Ontology (GO) enrichment analysis of down- and up-regulated genes across three principal ontologies. **A** The graph provides the enrichment scores from a Gene Ontology analysis focusing on down-regulated genes, categorized into the domains of Biological Processes (BP), Cellular Components (CC), and Molecular Functions (MF). The bars, color-coded as orange for BP, green for CC, and blue for MF, illustrate the degree of each GO term within the studied dataset. Notably, BP terms such as regulation of insulin secretion’ and ‘lipid homeostasis’ show the highest enrichment scores, indicating their significant down-regulation in the genomic profile. **B** The figure delineates a series of genes with their corresponding log fold changes (logFC), indicating a down-regulation in expression levels. The color-coded segments represent individual genes, while the connecting ribbons illustrate their binding to specific GO terms associated. The GO terms lists include cell-cell junctions, synaptic activity, and binding activities (such as calmodulin and glycosaminoglycan). **C** The chart depicts the enrichment scores derived from a GO analysis for up-regulated genes. Notably, “calmodulin-dependent protein kinase activity” in the MF category and “insulin secretion” in the BP category exhibit the highest enrichment scores, suggesting significant up-regulation in these functional areas. **D** The figure draws the genes with their corresponding logFC. GO terms associated with these genes are listed, including those related to cell-cell junctions, synaptic activity, and binding activities (such as calmodulin and glycosaminoglycan).

An RNA-Seq analysis was conducted to assess the impact of the LRRC8A deletion on HCT116 gene expression. A total of 125 genes displayed differential expression in LRRC8A KO versus WT cells: 56 genes being down-regulated and 69 up-regulated in LRRC8A KO cells (Table 1). Figure 3 shows the Gene Ontology (GO) enrichment scores for genes that were identified as either down-regulated or up-regulated. Panels 3A and 3C illustrate the GO enrichment for down-regulated and up-regulated genes, respectively. The enrichment bars are categorized by Biological Processes (BP) in orange, Cellular Components (CC) in green, and Molecular Functions (MF) in blue. Greater enrichment significance is indicated by longer bars, suggesting a more substantial association with the set of genes being analyzed. For example, in Fig. 3A, processes such as ‘regulation of insulin secretion’ show notable enrichment, indicating significant down-regulation. Figure 3C shows that the up-regulated genes are significantly enriched in processes such as ‘insulin secretion’ and components such as ‘cell junctions’. Functions such as ‘calmodulin binding’ are particularly prominent. Figures 3B and D display chord diagrams that visually represent the relationships between genes and their corresponding GO terms. Figure 3B shows down-regulated genes, while Fig. 3D shows up-regulated genes. The outer circle of each diagram lists genes, which are connected by coloured chords to specific GO terms that describe BP, CC, and MF. These chords display the network of associations between genes and their functions or locations within the cell. The chords are colour-matched with the corresponding GO categories they represent. On the gene side, a colour gradient illustrates the log-fold change (logFC) in gene expression. Warmer colours indicate a higher degree of down- or up-regulation. These diagrams summarise the complex interactions and functional implications of the observed changes in gene expression in the study.

**Table 1:** DEGs in LRRC8A KO CRC cells.

Differentially Expressed Genes (DEG)		
ADGRF1	ENSG00000153292	DOWN
ALDH8A1	ENSG00000118514	UP
ANKRD22	ENSG00000152766	DOWN
ANXA10	ENSG00000109511	UP
APOBEC3G	ENSG00000239713	UP
ARHGAP29	ENSG00000137962	DOWN
ARHGEF10L	ENSG00000074964	DOWN
ARRDC4	ENSG00000140450	UP
ATP2B2	ENSG00000157087	UP
B3GALT5	ENSG00000183778	UP
CACNB4	ENSG00000182389	UP
CAMKV	ENSG00000164076	UP
CCN3	ENSG00000136999	UP
CD24	ENSG00000272398	DOWN
CEMIP	ENSG00000103888	DOWN
CLMP	ENSG00000166250	UP
CPLX1	ENSG00000168993	UP
CRYBG1	ENSG00000112297	DOWN
CRYZL2P	ENSG00000242193	DOWN
DAPK1	ENSG00000196730	UP
DPP4	ENSG00000197635	UP
DYNLT1	ENSG00000146425	UP
EZR	ENSG00000092820	UP
FAM222A-AS1	ENSG00000255650	UP
FOXA2	ENSG00000125798	DOWN
FRMD4B	ENSG00000114541	DOWN
FRMPD3	ENSG00000147234	UP
GARIN5A	ENSG00000142530	DOWN
GGT5	ENSG00000099998	DOWN
GJA3	ENSG00000121743	UP
GPAT3	ENSG00000138678	UP
GPD1	ENSG00000167588	UP
GPR55	ENSG00000135898	UP
GREB1L	ENSG00000141449	UP
GTF2H5	ENSG00000272047	UP
HKDC1	ENSG00000156510	UP
HNF4A	ENSG00000101076	DOWN
IKZF2	ENSG00000030419	DOWN
IL18	ENSG00000150782	DOWN
ILDR2	ENSG00000143195	UP
INAVA	ENSG00000163362	DOWN
KCNQ3	ENSG00000184156	UP
KLF7	ENSG00000118263	DOWN
KLRK1-AS1	ENSG00000245648	DOWN
LCN2	ENSG00000148346	DOWN
LGR4	ENSG00000205213	UP
LINC00698	ENSG00000244342	DOWN
LINC02593	ENSG00000223764	UP
LNCOC1	ENSG00000253741	DOWN
LOC102723553	ENSG00000273590	DOWN
LPAR1	ENSG00000198121	DOWN
LRRC8A	ENSG00000136802	DOWN
LYPD3	ENSG00000124466	UP
MECOM + B58:B124	ENSG00000085276	UP
MEF2C	ENSG00000081189	UP
MGLL	ENSG00000074416	UP
MNS1	ENSG00000138587	DOWN
MTUS1-DT	ENSG00000253671	DOWN
MYRIP	ENSG00000170011	DOWN
NAPRT	ENSG00000147813	DOWN
NAV2	ENSG00000166833	DOWN
NAV3	ENSG00000067798	DOWN
NECTIN4	ENSG00000143217	UP
NEMP2-DT	ENSG00000233654	DOWN
NFIB	ENSG00000147862	DOWN
NR2F1	ENSG00000175745	DOWN
NR2F1-AS1	ENSG00000237187	DOWN
NRIP1	ENSG00000180530	DOWN
NRP2	ENSG00000118257	UP
NT5E	ENSG00000135318	DOWN
PALLD	ENSG00000129116	DOWN
PCAT2	ENSG00000254166	DOWN
PCDH19	ENSG00000165194	UP
PCOLCE2	ENSG00000163710	UP
PDE4B	ENSG00000184588	DOWN
PHYHD1	ENSG00000175287	UP
PKIB	ENSG00000135549	DOWN
PLXNA2	ENSG00000076356	UP
PURPL	ENSG00000250337	UP
PXDN	ENSG00000130508	UP
RBP1	ENSG00000114115	DOWN
RBPMS	ENSG00000157110	UP
RGS16	ENSG00000143333	UP
RGS2	ENSG00000116741	UP
RNF43	ENSG00000108375	DOWN
RTN4RL1	ENSG00000185924	UP
RUNX2	ENSG00000124813	UP
SAMD11	ENSG00000187634	UP
SERPING1	ENSG00000149131	UP
SLC16A6	ENSG00000108932	UP
SLC17A9	ENSG00000101194	UP
SLC5A5	ENSG00000105641	DOWN
SLCO3A1	ENSG00000176463	UP
SNAP25	ENSG00000132639	UP
SRGAP1	ENSG00000196935	DOWN
STK39	ENSG00000198648	DOWN
STYK1	ENSG00000060140	DOWN
SULF2	ENSG00000196562	UP
SULT1A4	ENSG00000213648	UP
SYT13	ENSG00000019505	UP
SYTL3	ENSG00000164674	UP
TACSTD2	ENSG00000184292	DOWN
TLL1	ENSG00000038295	UP
TMEM154	ENSG00000170006	UP
TMEM181	ENSG00000146433	UP
TMEM200A	ENSG00000164484	DOWN
TNFRSF10C	ENSG00000173535	UP
TNFSF18	ENSG00000120337	DOWN
TRAPPC9	ENSG00000167632	DOWN
TRHDE	ENSG00000072657	UP
TRPV2	ENSG00000187688	UP
TULP4	ENSG00000130338	UP
VGLL3	ENSG00000206538	UP
VSNL1	ENSG00000163032	DOWN
WNT5A	ENSG00000114251	UP
ZNF585B	ENSG00000245680	DOWN
ZNF595	ENSG00000272602	DOWN
ZNF704	ENSG00000164684	DOWN

A total of 125 genes displayed differential expression in LRRC8A KO versus WT cells. 56 genes being down-regulated and 69 up-regulated in LRRC8A KO cells.

**Fig. 4:**
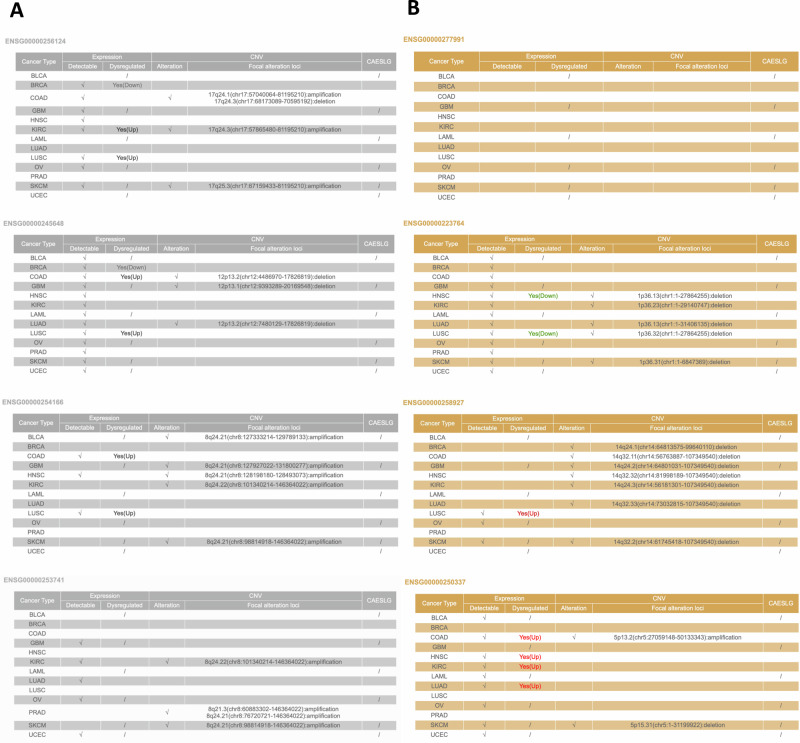
Expression Profiles of lncRNAs Across Various Cancer Types. The tables systematically compare the expression profiles of long non-coding RNAs (lncRNAs) across different cancer types. **A** and **B** illustrate, respectively, the downregulated and upregulated lncRNAs in various types of cancers. Each row represents a specific lncRNA, with columns indicating detectability, expression dysregulation, alterations, and the localization of focal alterations within the genome, as identified in the CAESLG database.

Furthermore, our attention was directed towards long non-coding RNAs (lncRNAs). Recent research has shown a significant correlation between aberrant expression patterns of lncRNAs and various complex human diseases, particularly cancer. The increasing repertoire of lncRNAs has led to their characterization as either ‘oncogenes’ or ‘tumor suppressors’. Dysregulation of lncRNAs has been linked to the initiation, progression, and metastasis of cancer [40, 41]. The Cancer LncRNome Atlas was used to investigate alterations in individual lncRNAs at transcriptional, genomic, and epigenetic levels in human cancers to identify relevant information (Table 2 and Fig. 4). The observed lncRNAs exhibited varied patterns of regulation across different cancer types, with some showing upregulation and others downregulation (Table 2 and Fig. 4). This differential expression, coupled with their location on the chromosomes as noted in the data, hints at the complex genetic architecture that underlies cancer.

**Table 2:** List of lncRNAs.

Regulation	Ensembl ID	log2 Fold Change	Adj.Pval	Symbol	Chr
**Down**	ENSG00000256124	−6.55320993004657	2.72e−03	LINC01152	17q24.3
**Down**	ENSG00000245648	−1.4700236550104	1.85e−05	KLRK1-AS1	12p13.2
**Down**	ENSG00000254166	−1.4339799975236	1.03e−05	CASC19	8q24.21
**Down**	ENSG00000253741	−1.07168286754586	2.20e−03	LNCOC1	8q24.3
**Up**	ENSG00000277991	4.24840538102438	9.66e−05		21p11.2
**Up**	ENSG00000258927	3.31905187015385	4.01e−03		14q32.13
**Up**	ENSG00000223764	1.49820395651212	3.96e−03	LINC02593	1p36.33
**Up**	ENSG00000250337	1.44062288967738	1.34e−03	PURPL	5p14.1

The expression is evaluated considering LRRC8A-KO vs WT in HCT116.

### Defining the LRRC8A interactome in living cells

**Fig. 5:**
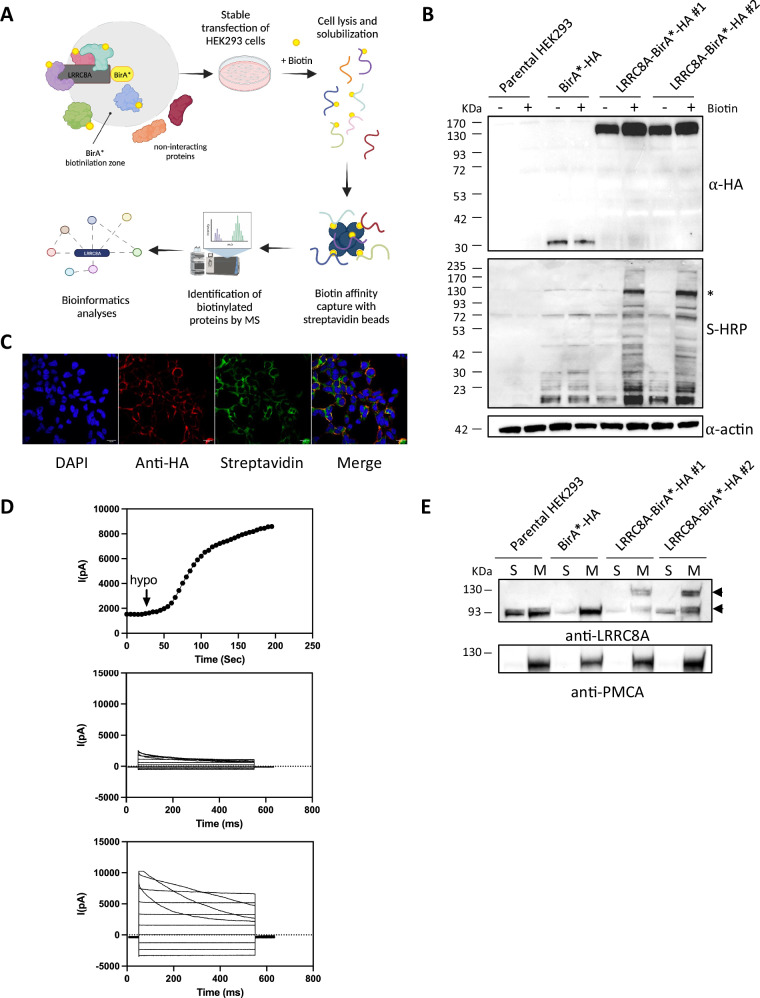
Characterization of cells stably expressing LRRC8A-BioID fusion protein. **A** Schematic representation of the BioID technique, a method for exploring protein complexes in live cells [114, 120]. Within the BioID methodology, the target protein is expressed in cells as a fusion with a specialized tagging enzyme, BirA R118G (a promiscuous mutant biotin ligase, hereinafter referred to as BirA*). This enzyme utilizes exogenous biotin to catalyse the formation of biotinoyl-5’-AMP, a highly reactive molecule that biotinylates primary amines, such as the lysine side chain, within a proximity of approximately 10 nm [121]. Subsequently, cells are lysed, and the labeled proteins are subjected to affinity purification, followed by detection through MS. The identification of pertinent biotinylated proteins is then accomplished through quantitative and statistical methodologies **B** Verification of BirA* activity in cells expressing LRRC8A-BirA*-HA by Western blot. The expression of the LRRC8A-BirA*-HA fusion protein was assessed using an anti-HA antibody in the absence and presence of biotin. BirA* activity in cells expressing LRRC8A-BirA*-HA was assessed by WB using streptavidin-HRP for the detection of biotinylated proteins. Activity was assessed in the presence and absence of biotin (50 μM), using HEK293 cells and cells transfected with pcDNA3.1 MCS-BirA(R118G)-HA (in the blot indicated as BirA*) as controls. Actin was used as a loading control. For each sample, 50 μg of total protein extract was loaded. **C** Validation of the subcellular localization of the LRRC8A-BirA*-HA protein by immunofluorescence. Nuclei were visualized by DAPI staining; α-HA antibody and Alexa Fluor 586-conjugated red-emitting secondary antibody were used to visualize LRRC8A-BirA*-HA; streptavidin-HRP was used to visualize biotinylated proteins. **D** Validation of LRRC8A-BirA*-HA protein channel activity by patch clamp: Representative time course of current activation upon perfusion with a hypotonic solution of cells co-expressing the 8A-BirA*-8E heteromers. **E** Validation of the subcellular localization of the LRRC8A-BirA*-HA protein by Western Blotting. The localization of the LRRC8A-BirA*-HA protein was assessed in the two clones and in HEK293 cells and cells transfected with pcDNA3.1 MCS-BirA(R118G)-HA using the α-LRRC8A antibody. For each sample, 50 μg of protein extract enriched with the cytoplasmic (S) or membrane (M) protein fraction was loaded. The α-PMCA antibody was used to exclude contamination of the S-fraction by proteins from the M-fraction.

To identify regulators of LRRC8A, we employed the BioID technique (Fig. 5A). WT human LRRC8A was fused with BirA*-HA and HEK293 cell lines stably expressing the LRRC8A-BirA*-HA fusion protein were generated. Stable expression was employed to maximize the formation of VRAC heteromers. The parental cell line and cells expressing solely the BirA* enzyme served as negative and positive controls, respectively. To validate the proper production of the BirA* protein fusion, we performed western blot experiments using Streptavidin-HRP to assess the degree of biotinylation in the presence and absence of exogenous biotin (Fig. 5B). As expected, distinct bands of varying sizes were observed in LRRC8A-BirA* cells in the presence of biotin. Conversely, minimal levels of biotinylation were detected in control lysates, both in the presence and absence of biotin (Fig. 5B). Subsequently, to verify that the genetic fusion of LRRC8A with BirA*-HA did not alter intracellular localization and channel function, we conducted confocal microscopy and patch-clamp experiments (Fig. 5C, D). The presence of BirA*-HA did not impact channel activity (Fig. 5D), consistent with observations for LRRC8A with a C-terminal GFP tag—a protein with a similar molecular weight and steric hindrance to the BirA* enzyme [18]. Fluorescence microscopy confirmed that the fusion protein localized to the plasma membrane similarly to endogenous channels (Fig. 5C) Biotinylation was further confirmed using Alexa Fluor 488 nm-conjugated streptavidin (Fig. 5C). Additionally, western blots confirmed the localization of the fusion protein in membranes (Fig. 5E).

**Fig. 6:**
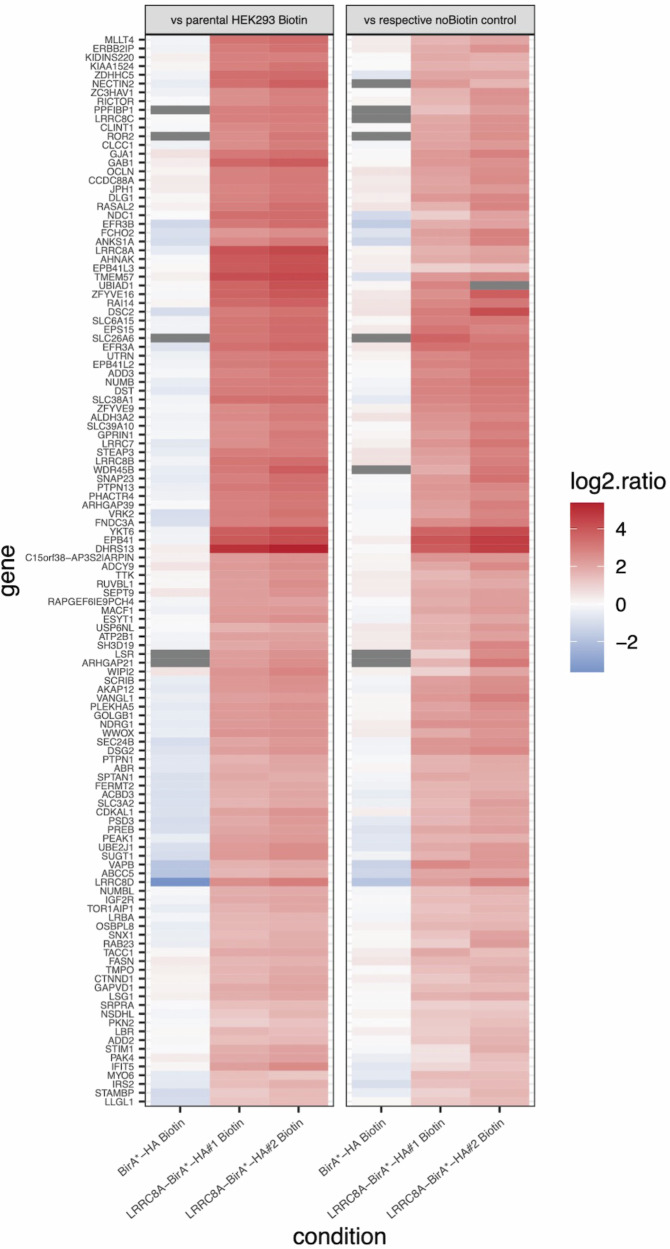
MS results: identification of hits proteins. Heatmap reporting the log2 fold changes as indicated in the heading (left column ratio against the parental HEK293 Biotin control and right column against the corresponding noBiotin control) for the 122 identified enriched proteins (hits).

Following the validation of the correct cellular localization and function of LRRC8A-BioID, purified biotinylated proteins were analysed with quantitative mass spectrometry (MS) using tandem mass tag labelling (TMT). The tagged proteins were isolated through streptavidin affinity purification, TMT labelled and analysed by LC-MS/MS. Raw files were analysed using IsobarQuant and Mascot (v2.2.07) (see overview of normalized TMT reporter ion intensities in Figure S1A for an overview of samples). Quantification of individual protein enrichment was determined by comparing their relative abundance in each LRRC8A-BirA* sample with the experimentally paired Venus-BirA* control (no Biotin control, see Volcano Plots in Figure S1B). To obtain a comprehensive understanding of the proximal protein environment surrounding the VRAC complex, a stringent cut-off was applied to the dataset. Only proteins quantified with two unique peptide matches were retained for analysis. Proteins were further tested for differential abundance using a moderated t-test by applying the limma R package. As a significance cut-off we used a false discovery rate (FDR) of less than 0.05 and a fold change (FC) of at least 2-fold. Out of 1227 proteins, we selected 122 enriched hits (around 10% of the total) with a significant up-biotinylation in the comparisons ‘LRRC8A-BirA*-HA#1 Biotin vs LRRC8A-BirA*-HA#1 noBiotin’ or ‘LRRC8A-BirA*-HA#2 Biotin vs LRRC8A-BirA*-HA#2 noBiotin’ (see Volcano Plots in Figure S1B, Fig. 6, Table 3).

**Table 3:** List and annotations of hit proteins from BioID analyses.

Protein	stringID	Annotation
ABCC5	9606.ENSP00000333926	Multidrug resistance-associated protein 5; Acts as a multispecific organic anion pump which can transport nucleotide analogs; Belongs to the ABC transporter superfamily. ABCC family. Conjugate transporter (TC 3.A.1.208) subfamily
ABR	9606.ENSP00000303909	Active breakpoint cluster region-related protein; GTPase-activating protein for RAC and CDC42. Promotes the exchange of RAC or CDC42-bound GDP by GTP, thereby activating them; C2 domain containing
ACBD3	9606.ENSP00000355777	Acyl-coa binding domain containing 3; Golgi resident protein GCP60; Involved in the maintenance of Golgi structure by interacting with giantin, affecting protein transport between the endoplasmic reticulum and Golgi. Involved in hormone-induced steroid biosynthesis in testicular Leydig cells (By similarity). Recruits PI4KB to the Golgi apparatus membrane; enhances the enzyme activity of PI4KB activity via its membrane recruitment thereby increasing the local concentration of the substrate in the vicinity of the kinase; A-kinase anchoring proteins
ADD2	9606.ENSP00000264436	Beta-adducin; Membrane-cytoskeleton-associated protein that promotes the assembly of the spectrin-actin network. Binds to the erythrocyte membrane receptor SLC2A1/GLUT1 and may therefore provide a link between the spectrin cytoskeleton to the plasma membrane. Binds to calmodulin. Calmodulin binds preferentially to the beta subunit; Belongs to the aldolase class II family.
ADD3	9606.ENSP00000348381	Gamma-adducin; Membrane-cytoskeleton-associated protein that promotes the assembly of the spectrin-actin network. Plays a role in actin filament capping. Binds to calmodulin; Belongs to the aldolase class II family. Adducin subfamily
AHNAK	9606.ENSP00000367263	Neuroblast differentiation-associated protein AHNAK; May be required for neuronal cell differentiation; PDZ domain containing
AKAP12	9606.ENSP00000384537	A-kinase anchor protein 12; Anchoring protein that mediates the subcellular compartmentation of protein kinase A (PKA) and protein kinase C (PKC); A-kinase anchoring proteins
ALDH3A2	9606.ENSP00000345774	Aldehyde dehydrogenase 3 family member a2; Fatty aldehyde dehydrogenase; Catalyzes the oxidation of long-chain aliphatic aldehydes to fatty acids. Active on a variety of saturated and unsaturated aliphatic aldehydes between 6 and 24 carbons in length. Responsible for conversion of the sphingosine 1-phosphate (S1P) degradation product hexadecenal to hexadecenoic acid
ANKS1A	9606.ENSP00000353518	Ankyrin repeat and SAM domain-containing protein 1A; Regulator of different signaling pathways. Regulates EPHA8 receptor tyrosine kinase signaling to control cell migration and neurite retraction (By similarity); Ankyrin repeat domain containing
ARHGAP21	9606.ENSP00000379709	Rho GTPase-activating protein 21; Functions as a GTPase-activating protein (GAP) for RHOA and CDC42. Downstream partner of ARF1 which may control Golgi apparatus structure and function. Also required for CTNNA1 recruitment to adherens junctions; PDZ domain containing
ARHGAP39	9606.ENSP00000366522	Rho gtpase-activating protein 39; Rho GTPase activating protein 39
ATP2B1	9606.ENSP00000392043	Plasma membrane calcium-transporting ATPase 1; This magnesium-dependent enzyme catalyzes the hydrolysis of ATP coupled with the transport of calcium out of the cell; ATPases Ca2+ transporting
CCDC88A	9606.ENSP00000338728	Coiled-coil domain containing 88a; Girdin; Plays a role as a key modulator of the AKT-mTOR signaling pathway controlling the tempo of the process of newborn neurons integration during adult neurogenesis, including correct neuron positioning, dendritic development and synapse formation (By similarity). Enhances phosphoinositide 3-kinase (PI3K)- dependent phosphorylation and kinase activity of AKT1/PKB, but does not possess kinase activity itself (By similarity). Phosphorylation of AKT1/PKB thereby induces the phosphorylation of downstream effectors GSK3 and FOXO1/FKHR
CDKAL1	9606.ENSP00000274695	Threonylcarbamoyladenosine tRNA methylthiotransferase; Catalyzes the methylthiolation of N6- threonylcarbamoyladenosine (t(6)A), leading to the formation of 2- methylthio-N6-threonylcarbamoyladenosine (ms(2)t(6)A) at position 37 in tRNAs that read codons beginning with adenine; Belongs to the methylthiotransferase family. CDKAL1 subfamily
CLCC1	9606.ENSP00000349456	Chloride channel CLIC-like protein 1; Seems to act as a chloride ion channel; Tetraspan junctional complex superfamily
CLINT1	9606.ENSP00000429824	Clathrin interactor 1; Binds to membranes enriched in phosphatidylinositol 4,5- bisphosphate (PtdIns(4,5)P2). May have a role in transport via clathrin-coated vesicles from the trans-Golgi network to endosomes. Stimulates clathrin assembly
CTNND1	9606.ENSP00000382004	Catenin delta-1; Binds to and inhibits the transcriptional repressor ZBTB33, which may lead to activation of target genes of the Wnt signaling pathway (By similarity). Associates with and regulates the cell adhesion properties of both C-, E- and N-cadherins, being critical for their surface stability. Implicated both in cell transformation by SRC and in ligand-induced receptor signaling through the EGF, PDGF, CSF-1 and ERBB2 receptors. Promotes GLIS2 C-terminal cleavage; Belongs to the beta-catenin family
DHRS13	9606.ENSP00000368173	Dehydrogenase/reductase SDR family member 13; Putative oxidoreductase
DLG1	9606.ENSP00000345731	Disks large homolog 1; Essential multidomain scaffolding protein required for normal development (By similarity). Recruits channels, receptors and signaling molecules to discrete plasma membrane domains in polarized cells. May play a role in adherens junction assembly, signal transduction, cell proliferation, synaptogenesis and lymphocyte activation. Regulates the excitability of cardiac myocytes by modulating the functional expression of Kv4 channels. Functional regulator of Kv1.5 channel; Belongs to the MAGUK family
DSC2	9606.ENSP00000280904	Desmocollin-2; Component of intercellular desmosome junctions. Involved in the interaction of plaque proteins and intermediate filaments mediating cell-cell adhesion. May contribute to epidermal cell positioning by mediating differential adhesiveness between cells that express different isoforms
DSG2	9606.ENSP00000261590	Desmoglein-2; Component of intercellular desmosome junctions. Involved in the interaction of plaque proteins and intermediate filaments mediating cell-cell adhesion
DST	9606.ENSP00000307959	Dystonin; Cytoskeletal linker protein. Acts as an integrator of intermediate filaments, actin and microtubule cytoskeleton networks. Required for anchoring either intermediate filaments to the actin cytoskeleton in neural and muscle cells or keratin- containing intermediate filaments to hemidesmosomes in epithelial cells. The proteins may self-aggregate to form filaments or a two- dimensional mesh. Regulates the organization and stability of the microtubule network of sensory neurons to allow axonal transport. Mediates docking of the dynein/dynactin motor complex to vesicle cargos
EFR3A	9606.ENSP00000254624	Protein EFR3 homolog A; Component of a complex required to localize phosphatidylinositol 4-kinase (PI4K) to the plasma membrane. The complex acts as a regulator of phosphatidylinositol 4-phosphate (PtdIns(4)P) synthesis (Probable). In the complex, EFR3A probably acts as the membrane-anchoring component. Also involved in responsiveness to G-protein-coupled receptors; it is however unclear whether this role is direct or indirect
EFR3B	9606.ENSP00000384081	Protein EFR3 homolog B; Component of a complex required to localize phosphatidylinositol 4-kinase (PI4K) to the plasma membrane. The complex acts as a regulator of phosphatidylinositol 4-phosphate (PtdIns(4)P) synthesis (Probable). In the complex, EFR3B probably acts as the membrane-anchoring component. Also involved in responsiveness to G-protein-coupled receptors; it is however unclear whether this role is direct or indirect; Armadillo-like helical domain containing
EPB41	9606.ENSP00000345259	Protein 4.1; Protein 4.1 is a major structural element of the erythrocyte membrane skeleton. It plays a key role in regulating membrane physical properties of mechanical stability and deformability by stabilizing spectrin-actin interaction. Recruits DLG1 to membranes. Required for dynein-dynactin complex and NUMA1 recruitment at the mitotic cell cortex during anaphase
EPB41L2	9606.ENSP00000338481	Band 4.1-like protein 2; Required for dynein-dynactin complex and NUMA1 recruitment at the mitotic cell cortex during anaphase; Erythrocyte membrane protein band 4.1
EPB41L3	9606.ENSP00000343158	Band 4.1-like protein 3; Tumor suppressor that inhibits cell proliferation and promotes apoptosis. Modulates the activity of protein arginine N- methyltransferases, including PRMT3 and PRMT5; Erythrocyte membrane protein band 4.1
EPS15	9606.ENSP00000360798	Epidermal growth factor receptor substrate 15; Involved in cell growth regulation. May be involved in the regulation of mitogenic signals and control of cell proliferation. Involved in the internalization of ligand-inducible receptors of the receptor tyrosine kinase (RTK) type, in particular EGFR. Plays a role in the assembly of clathrin-coated pits (CCPs). Acts as a clathrin adapter required for post-Golgi trafficking. Seems to be involved in CCPs maturation including invagination or budding. Involved in endocytosis of integrin beta- 1 (ITGB1) and transferrin receptor (TFR)
ERBB2IP	9606.ENSP00000426632	Erbin; Acts as an adapter for the receptor ERBB2, in epithelia. By binding the unphosphorylated ‘Tyr-1248’ of receptor ERBB2, it may contribute to stabilize this unphosphorylated state. Inhibits NOD2-dependent NF-kappa-B signaling and proinflammatory cytokine secretion; Belongs to the LAP (LRR and PDZ) protein family
ESYT1	9606.ENSP00000267113	Extended synaptotagmin-1; Binds glycerophospholipids in a barrel-like domain and may play a role in cellular lipid transport (By similarity). Binds calcium (via the C2 domains) and translocates to sites of contact between the endoplasmic reticulum and the cell membrane in response to increased cytosolic calcium levels. Helps tether the endoplasmic reticulum to the cell membrane and promotes the formation of appositions between the endoplasmic reticulum and the cell membrane; Belongs to the extended synaptotagmin family
FASN	9606.ENSP00000304592	Fatty acid synthase; Fatty acid synthetase catalyzes the formation of long- chain fatty acids from acetyl-CoA, malonyl-CoA and NADPH. This multifunctional protein has 7 catalytic activities and an acyl carrier protein; Seven-beta-strand methyltransferase motif containing
FCHO2	9606.ENSP00000393776	F-BAR domain only protein 2; Functions in an early step of clathrin-mediated endocytosis. Has both a membrane binding/bending activity and the ability to recruit proteins essential to the formation of functional clathrin-coated pits. Has a lipid-binding activity with a preference for membranes enriched in phosphatidylserine and phosphoinositides (Pi(4,5) biphosphate) like the plasma membrane. Its membrane-bending activity might be important for the subsequent action of clathrin and adaptors in the formation of clathrin-coated vesicles
FERMT2	9606.ENSP00000342858	Fermitin family homolog 2; Scaffolding protein that enhances integrin activation mediated by TLN1 and/or TLN2, but activates integrins only weakly by itself. Binds to membranes enriched in phosphoinositides. Enhances integrin-mediated cell adhesion onto the extracellular matrix and cell spreading; this requires both its ability to interact with integrins and with phospholipid membranes. Required for the assembly of focal adhesions. Participates in the connection between extracellular matrix adhesion sites and the actin cytoskeleton
FNDC3A	9606.ENSP00000441831	Fibronectin type-III domain-containing protein 3A; Mediates spermatid-Sertoli adhesion during spermatogenesis; Belongs to the FNDC3 family
GAB1	9606.ENSP00000262995	GRB2-associated-binding protein 1; Adapter protein that plays a role in intracellular signaling cascades triggered by activated receptor-type kinases. Plays a role in FGFR1 signaling. Probably involved in signaling by the epidermal growth factor receptor (EGFR) and the insulin receptor (INSR)
GAPVD1	9606.ENSP00000377664	GTPase-activating protein and VPS9 domain-containing protein 1; Acts both as a GTPase-activating protein (GAP) and a guanine nucleotide exchange factor (GEF), and participates in various processes such as endocytosis, insulin receptor internalization or LC2A4/GLUT4 trafficking. Acts as a GEF for the Ras-related protein RAB31 by exchanging bound GDP for free GTP, leading to regulate LC2A4/GLUT4 trafficking. In the absence of insulin, it maintains RAB31 in an active state and promotes a futile cycle between LC2A4/GLUT4 storage vesicles and early endosomes
GJA1	9606.ENSP00000282561	Gap junction alpha-1 protein; Gap junction protein that acts as a regulator of bladder capacity. A gap junction consists of a cluster of closely packed pairs of transmembrane channels, the connexons, through which materials of low MW diffuse from one cell to a neighboring cell. May play a critical role in the physiology of hearing by participating in the recycling of potassium to the cochlear endolymph. Negative regulator of bladder functional capacity: acts by enhancing intercellular electrical and chemical transmission
GOLGB1	9606.ENSP00000377275	Golgin subfamily B member 1; May participate in forming intercisternal cross-bridges of the Golgi complex
GPRIN1	9606.ENSP00000305839	G protein-regulated inducer of neurite outgrowth 1; May be involved in neurite outgrowth
IFIT5	9606.ENSP00000360860	Interferon-induced protein with tetratricopeptide repeats 5; Interferon-induced RNA-binding protein involved in the human innate immune response. Has a broad and adaptable RNA structure recognition important for RNA recognition specificity in antiviral defense. Binds precursor and processed tRNAs as well as poly-U-tailed tRNA fragments. Specifically binds single-stranded RNA bearing a 5’-triphosphate group (PPP-RNA), thereby acting as a sensor of viral single-stranded RNAs.
IGF2R	9606.ENSP00000349437	Cation-independent mannose-6-phosphate receptor; Transport of phosphorylated lysosomal enzymes from the Golgi complex and the cell surface to lysosomes. Lysosomal enzymes bearing phosphomannosyl residues bind specifically to mannose-6- phosphate receptors in the Golgi apparatus and the resulting receptor-ligand complex is transported to an acidic prelyosomal compartment where the low pH mediates the dissociation of the complex. This receptor also binds IGF2. Acts as a positive regulator of T-cell coactivation, by binding DPP4; CD molecules
IRS2	9606.ENSP00000365016	Insulin receptor substrate 2; May mediate the control of various cellular processes by insulin; Pleckstrin homology domain containing
JPH1	9606.ENSP00000344488	Junctophilin-1; Junctophilins contribute to the formation of junctional membrane complexes (JMCs) which link the plasma membrane with the endoplasmic or sarcoplasmic reticulum in excitable cells. Provides a structural foundation for functional cross-talk between the cell surface and intracellular calcium release channels. JPH1 contributes to the construction of the skeletal muscle triad by linking the t-tubule (transverse-tubule) and SR (sarcoplasmic reticulum) membranes
KIAA1524	9606.ENSP00000295746	Cellular inhibitor of pp2a; Protein CIP2A; Oncoprotein that inhibits PP2A and stabilizes MYC in human malignancies. Promotes anchorage-independent cell growth and tumor formation
KIDINS220	9606.ENSP00000256707	Ankyrin repeat-rich membrane spanning protein; Kinase D-interacting substrate of 220 kDa; Promotes a prolonged MAP-kinase signaling by neurotrophins through activation of a Rap1-dependent mechanism. Provides a docking site for the CRKL-C3G complex, resulting in Rap1-dependent sustained ERK activation. May play an important role in regulating postsynaptic signal transduction through the syntrophin-mediated localization of receptor tyrosine kinases such as EPHA4. In cooperation with SNTA1 can enhance EPHA4-induced JAK/STAT activation.
LBR	9606.ENSP00000339883	Delta14-sterol reductase (lamin-B receptor); Lamin-B receptor; Anchors the lamina and the heterochromatin to the inner nuclear membrane; Tudor domain containing
LLGL1	9606.ENSP00000321537	LLGL1, scribble cell polarity complex component; Lethal(2) giant larvae protein homolog 1; Cortical cytoskeleton protein found in a complex involved in maintaining cell polarity and epithelial integrity. Involved in the regulation of mitotic spindle orientation, proliferation, differentiation and tissue organization of neuroepithelial cells. Involved in axonogenesis through RAB10 activation thereby regulating vesicular membrane trafficking toward the axonal plasma membrane
LRBA	9606.ENSP00000349629	Lipopolysaccharide-responsive and beige-like anchor protein; May be involved in coupling signal transduction and vesicle trafficking to enable polarized secretion and/or membrane deposition of immune effector molecules; Armadillo-like helical domain containing
LRRC7	9606.ENSP00000035383	Leucine-rich repeat-containing protein 7; Required for normal synaptic spine architecture and function. Necessary for DISC1 and GRM5 localization to postsynaptic density complexes and for both N-methyl D-aspartate receptor-dependent and metabotropic glutamate receptor-dependent long term depression; Belongs to the LAP (LRR and PDZ) protein family
LRRC8B	9606.ENSP00000332674	Volume-regulated anion channel subunit LRRC8B; Non-essential component of the volume-regulated anion channel (VRAC, also named VSOAC channel), an anion channel required to maintain a constant cell volume in response to extracellular or intracellular osmotic changes. The VRAC channel conducts iodide better than chloride and may also conduct organic osmolytes like taurine. Channel activity requires LRRC8A plus at least one other family member (LRRC8B, LRRC8C, LRRC8D or LRRC8E); channel characteristics depend on the precise subunit composition
LRRC8C	9606.ENSP00000359483	Volume-regulated anion channel subunit LRRC8C; Non-essential component of the volume-regulated anion channel (VRAC, also named VSOAC channel), an anion channel required to maintain a constant cell volume in response to extracellular or intracellular osmotic changes. The VRAC channel conducts iodide better than chloride and may also conduct organic osmolytes like taurine. Channel activity requires LRRC8A plus at least one other family member (LRRC8B, LRRC8C, LRRC8D or LRRC8E); channel characteristics depend on the precise subunit composition
LRRC8D	9606.ENSP00000338887	Volume-regulated anion channel subunit LRRC8D; Non-essential component of the volume-regulated anion channel (VRAC, also named VSOAC channel), an anion channel required to maintain a constant cell volume in response to extracellular or intracellular osmotic changes. The VRAC channel conducts iodide better than chloride and may also conduct organic osmolytes like taurine. Channel activity requires LRRC8A plus at least one other family member (LRRC8B, LRRC8C, LRRC8D or LRRC8E); channel characteristics depend on the precise subunit composition
LSG1	9606.ENSP00000265245	Large subunit GTPase 1 homolog; GTPase required for the XPO1/CRM1-mediated nuclear export of the 60 S ribosomal subunit. Probably acts by mediating the release of NMD3 from the 60 S ribosomal subunit after export into the cytoplasm
LSR	9606.ENSP00000480821	Lipolysis-stimulated lipoprotein receptor; Probable role in the clearance of triglyceride-rich lipoprotein from blood. Binds chylomicrons, LDL and VLDL in presence of free fatty acids and allows their subsequent uptake in the cells (By similarity); Belongs to the immunoglobulin superfamily. LISCH7 family
MACF1	9606.ENSP00000354573	Microtubule-actin cross-linking factor 1, isoforms 1/2/3/5; Isoform 2: F-actin-binding protein which plays a role in cross-linking actin to other cytoskeletal proteins and also binds to microtubules. Plays an important role in ERBB2-dependent stabilization of microtubules at the cell cortex. Acts as a positive regulator of Wnt receptor signaling pathway and is involved in the translocation of AXIN1 and its associated complex (composed of APC, CTNNB1 and GSK3B) from the cytoplasm to the cell membrane (By similarity).
MLLT4	9606.ENSP00000375960	Afadin; Belongs to an adhesion system, probably together with the E-cadherin-catenin system, which plays a role in the organization of homotypic, interneuronal and heterotypic cell-cell adherens junctions (AJs). Nectin- and actin-filament-binding protein that connects nectin to the actin cytoskeleton
MYO6	9606.ENSP00000358994	Unconventional myosin-VI; Myosins are actin-based motor molecules with ATPase activity. Unconventional myosins serve in intracellular movements. Myosin 6 is a reverse-direction motor protein that moves towards the minus-end of actin filaments. Has slow rate of actin-activated ADP release due to weak ATP binding. Functions in a variety of intracellular processes such as vesicular membrane trafficking and cell migration. Required for the structural integrity of the Golgi apparatus via the p53-dependent pro-survival pathway.
NDC1	9606.ENSP00000360483	NDC1 transmembrane nucleoporin; Nucleoporin NDC1; Component of the nuclear pore complex (NPC), which plays a key role in de novo assembly and insertion of NPC in the nuclear envelope. Required for NPC and nuclear envelope assembly, possibly by forming a link between the nuclear envelope membrane and soluble nucleoporins, thereby anchoring the NPC in the membrane
NDRG1	9606.ENSP00000404854	N-myc downstream regulated 1; Protein NDRG1; Stress-responsive protein involved in hormone responses, cell growth, and differentiation. Acts as a tumor suppressor in many cell types. Necessary but not sufficient for p53/TP53- mediated caspase activation and apoptosis. Has a role in cell trafficking, notably of the Schwann cell, and is necessary for the maintenance and development of the peripheral nerve myelin sheath. Required for vesicular recycling of CDH1 and TF. May also function in lipid trafficking. Protects cells from spindle disruption damage.
NECTIN2	9606.ENSP00000252483	Nectin-2; Modulator of T-cell signaling. Can be either a costimulator of T-cell function, or a coinhibitor, depending on the receptor it binds to. Upon binding to CD226, stimulates T-cell proliferation and cytokine production, including that of IL2, IL5, IL10, IL13, and IFNG. Upon interaction with PVRIG, inhibits T-cell proliferation. These interactions are competitive. Probable cell adhesion protein; Belongs to the nectin family
NSDHL	9606.ENSP00000359297	Sterol-4-alpha-carboxylate 3-dehydrogenase, decarboxylating; Involved in the sequential removal of two C-4 methyl groups in post-squalene cholesterol biosynthesis; Short chain dehydrogenase/reductase superfamily
NUMB	9606.ENSP00000451300	Numb, endocytic adaptor protein; Protein numb homolog; Plays a role in the process of neurogenesis. Required throughout embryonic neurogenesis to maintain neural progenitor cells, also called radial glial cells (RGCs), by allowing their daughter cells to choose progenitor over neuronal cell fate. Not required for the proliferation of neural progenitor cells before the onset of neurogenesis. Also involved postnatally in the subventricular zone (SVZ) neurogenesis by regulating SVZ neuroblasts survival and ependymal wall integrity. May also mediate local repair of brain ventricular wall damage
NUMBL	9606.ENSP00000252891	Numb-like protein; Plays a role in the process of neurogenesis. Required throughout embryonic neurogenesis to maintain neural progenitor cells, also called radial glial cells (RGCs), by allowing their daughter cells to choose progenitor over neuronal cell fate. Not required for the proliferation of neural progenitor cells before the onset of embryonic neurogenesis. Also required postnatally in the subventricular zone (SVZ) neurogenesis by regulating SVZ neuroblasts survival and ependymal wall integrity. Negative regulator of NF-kappa-B signaling pathway. The inhibition of NF- kappa-B a […]
OCLN	9606.ENSP00000347379	Occludin; May play a role in the formation and regulation of the tight junction (TJ) paracellular permeability barrier. It is able to induce adhesion when expressed in cells lacking tight junctions; Protein phosphatase 1 regulatory subunits
OSBPL8	9606.ENSP00000261183	Oxysterol-binding protein-related protein 8; Lipid transporter involved in lipid countertransport between the endoplasmic reticulum and the plasma membrane: specifically exchanges phosphatidylserine with phosphatidylinositol 4-phosphate (PI4P), delivering phosphatidylserine to the plasma membrane in exchange for PI4P, which is degraded by the SAC1/SACM1L phosphatase in the endoplasmic reticulum. Binds phosphatidylserine and PI4P in a mutually exclusive manner. Binds oxysterol, 25- hydroxycholesterol and cholesterol; Belongs to the OSBP family
PAK4	9606.ENSP00000469413	Serine/threonine-protein kinase PAK 4; Serine/threonine protein kinase that plays a role in a variety of different signaling pathways including cytoskeleton regulation, cell migration, growth, proliferation or cell survival. Activation by various effectors including growth factor receptors or active CDC42 and RAC1 results in a conformational change and a subsequent autophosphorylation on several serine and/or threonine residues. Phosphorylates and inactivates the protein phosphatase SSH1, leading to increased inhibitory phosphorylation of the actin binding/depolymerizing factor cofilin
PEAK1	9606.ENSP00000452796	Pseudopodium-enriched atypical kinase 1; Tyrosine kinase that may play a role in cell spreading and migration on fibronectin. May directly or indirectly affect phosphorylation levels of cytoskeleton-associated proteins MAPK1/ERK and PXN
PHACTR4	9606.ENSP00000362942	Phosphatase and actin regulator 4; Regulator of protein phosphatase 1 (PP1) required for neural tube and optic fissure closure, and enteric neural crest cell (ENCCs) migration during development. Acts as an activator of PP1 by interacting with PPP1CA and preventing phosphorylation of PPP1CA at ‘Thr-320’. During neural tube closure, localizes to the ventral neural tube and activates PP1, leading to down-regulate cell proliferation within cranial neural tissue and the neural retina. Also acts as a regulator of migration of enteric neural crest cells (ENCCs) by activating PP1
PKN2	9606.ENSP00000359552	Serine/threonine-protein kinase N2; PKC-related serine/threonine-protein kinase and Rho/Rac effector protein that participates in specific signal transduction responses in the cell. Plays a role in the regulation of cell cycle progression, actin cytoskeleton assembly, cell migration, cell adhesion, tumor cell invasion and transcription activation signaling processes. Phosphorylates CTTN in hyaluronan-induced astrocytes and hence decreases CTTN ability to associate with filamentous actin. Phosphorylates HDAC5, therefore lead to impair HDAC5 import.
PLEKHA5	9606.ENSP00000404296	Pleckstrin homology domain-containing family a member 5; Pleckstrin homology domain containing A5
PPFIBP1	9606.ENSP00000314724	Ppfia binding protein 1; Liprin-beta-1; May regulate the disassembly of focal adhesions. Did not bind receptor-like tyrosine phosphatases type 2 A; Sterile alpha motif domain containing
PREB	9606.ENSP00000260643	Prolactin regulatory element-binding protein; Guanine nucleotide exchange factor that specifically activates the small GTPase SAR1B. Mediates the recruitement of SAR1B and other COPII coat components to endoplasmic reticulum membranes and is therefore required for the formation of COPII transport vesicles from the ER; WD repeat domain containing
PSD3	9606.ENSP00000324127	PH and SEC7 domain-containing protein 3; Guanine nucleotide exchange factor for ARF6; Pleckstrin homology domain containing
PTPN1	9606.ENSP00000360683	Tyrosine-protein phosphatase non-receptor type 1; Tyrosine-protein phosphatase which acts as a regulator of endoplasmic reticulum unfolded protein response. Mediates dephosphorylation of EIF2AK3/PERK; inactivating the protein kinase activity of EIF2AK3/PERK. May play an important role in CKII- and p60c-src-induced signal transduction cascades. May regulate the EFNA5-EPHA3 signaling pathway which modulates cell reorganization and cell-cell repulsion. May also regulate the hepatocyte growth factor receptor signaling pathway through dephosphorylation of MET
PTPN13	9606.ENSP00000394794	Tyrosine-protein phosphatase non-receptor type 13; Tyrosine phosphatase which regulates negatively FAS- induced apoptosis and NGFR-mediated pro-apoptotic signaling. May regulate phosphoinositide 3-kinase (PI3K) signaling through dephosphorylation of PIK3R2; FERM domain containing
RAB23	9606.ENSP00000417610	RAB23, member RAS oncogene family; Ras-related protein Rab-23; The small GTPases Rab are key regulators of intracellular membrane trafficking, from the formation of transport vesicles to their fusion with membranes. Rabs cycle between an inactive GDP-bound form and an active GTP-bound form that is able to recruit to membranes different set of downstream effectors directly responsible for vesicle formation, movement, tethering and fusion. Together with SUFU, prevents nuclear import of GLI1, and thereby inhibits GLI1 transcription factor activity.
RAI14	9606.ENSP00000427123	Retinoic acid induced 14; Ankycorbin; Plays a role in actin regulation at the ectoplasmic specialization, a type of cell junction specific to testis. Important for establishment of sperm polarity and normal spermatid adhesion. May also promote integrity of Sertoli cell tight junctions at the blood-testis barrier; Ankyrin repeat domain containing
RAPGEF6	9606.ENSP00000296859	Rap guanine nucleotide exchange factor 6; Guanine nucleotide exchange factor (GEF) for Rap1A, Rap2A and M-Ras GTPases. Does not interact with cAMP; PDZ domain containing
RASAL2	9606.ENSP00000356621	Ras GTPase-activating protein nGAP; Inhibitory regulator of the Ras-cyclic AMP pathway; C2 and RasGAP domain containing
RICTOR	9606.ENSP00000296782	Rapamycin-insensitive companion of mTOR; Subunit of mTORC2, which regulates cell growth and survival in response to hormonal signals. mTORC2 is activated by growth factors, but, in contrast to mTORC1, seems to be nutrient- insensitive. mTORC2 seems to function upstream of Rho GTPases to regulate the actin cytoskeleton, probably by activating one or more Rho-type guanine nucleotide exchange factors. mTORC2 promotes the serum-induced formation of stress-fibers or F-actin. mTORC2 plays a critical role in AKT1 ‘
ROR2	9606.ENSP00000364860	Tyrosine-protein kinase transmembrane receptor ROR2; Tyrosine-protein kinase receptor which may be involved in the early formation of the chondrocytes. It seems to be required for cartilage and growth plate development (By similarity). Phosphorylates YWHAB, leading to induction of osteogenesis and bone formation. In contrast, has also been shown to have very little tyrosine kinase activity in vitro. May act as a receptor for wnt ligand WNT5A which may result in the inhibition of WNT3A-mediated signaling; I-set domain containing
RUVBL1	9606.ENSP00000318297	RuvB-like 1; May be able to bind plasminogen at cell surface and enhance plasminogen activation; AAA ATPases
SCRIB	9606.ENSP00000349486	Protein scribble homolog; Scaffold protein involved in different aspects of polarized cells differentiation regulating epithelial and neuronal morphogenesis. Most probably functions in the establishment of apico-basal cell polarity. May function in cell proliferation regulating progression from G1 to S phase and as a positive regulator of apoptosis for instance during acinar morphogenesis of the mammary epithelium. May also function in cell migration and adhesion and hence regulate cell invasion through MAPK signaling. May play a role in exocytosis and in the targeting synaptic vesicle
SEC24B	9606.ENSP00000428564	Protein transport protein Sec24B; Component of the coat protein complex II (COPII) which promotes the formation of transport vesicles from the endoplasmic reticulum (ER). The coat has two main functions, the physical deformation of the endoplasmic reticulum membrane into vesicles and the selection of cargo molecules for their transport to the Golgi complex. Plays a central role in cargo selection within the COPII complex and together with SEC24A may have a different specificity compared to SEC24C and SEC24D. May package preferentially cargos with cytoplasmic DxE or LxxLE motifs
SEPT9	9606.ENSP00000391249	Septin-9; Filament-forming cytoskeletal GTPase (By similarity). May play a role in cytokinesis (Potential). May play a role in the internalization of 2 intracellular microbial pathogens. Belongs to the TRAFAC class TrmE-Era-EngA-EngB-Septin- like GTPase superfamily. Septin GTPase family
SH3D19	9606.ENSP00000302913	SH3 domain-containing protein 19; May play a role in regulating A disintegrin and metalloproteases (ADAMs) in the signaling of EGFR-ligand shedding. May be involved in suppression of Ras-induced cellular transformation and Ras-mediated activation of ELK1. Plays a role in the regulation of cell morphology and cytoskeletal organization
SLC26A6	9606.ENSP00000378920	Solute carrier family 26 member 6; Apical membrane anion-exchanger with wide epithelial distribution that plays a role as a component of the pH buffering system for maintaining acid-base homeostasis. Acts as a versatile DIDS-sensitive inorganic and organic anion transporter that mediates the uptake of monovalent anions like chloride, bicarbonate, formate and hydroxyl ion and divalent anions like sulfate and oxalate. Function in multiple exchange modes involving pairs of these anions
SLC38A1	9606.ENSP00000449756	Sodium-coupled neutral amino acid transporter 1; Functions as a sodium-dependent amino acid transporter. Mediates the saturable, pH-sensitive and electrogenic cotransport of glutamine and sodium ions with a stoichiometry of 1:1. May also transport small zwitterionic and aliphatic amino acids with a lower affinity. May supply glutamatergic and GABAergic neurons with glutamine which is required for the synthesis of the neurotransmitters glutamate and GABA; Solute carriers
SLC39A10	9606.ENSP00000386766	Solute carrier family 39 (zinc transporter), member 10; Zinc transporter ZIP10; May act as a zinc-influx transporter; Belongs to the ZIP transporter (TC 2.A.5) family
SLC3A2	9606.ENSP00000367123	4F2 cell-surface antigen heavy chain; Required for the function of light chain amino-acid transporters. Involved in sodium-independent, high-affinity transport of large neutral amino acids such as phenylalanine, tyrosine, leucine, arginine and tryptophan. Involved in guiding and targeting of LAT1 and LAT2 to the plasma membrane. When associated with SLC7A6 or SLC7A7 acts as an arginine/glutamine exchanger, following an antiport mechanism for amino acid transport, influencing arginine release in exchange for extracellular amino acids.
SLC6A15	9606.ENSP00000266682	Sodium-dependent neutral amino acid transporter B(0)AT2; Functions as a sodium-dependent neutral amino acid transporter. Exhibits preference for the branched-chain amino acids, particularly leucine, valine and isoleucine and methionine. Mediates the saturable, pH-sensitive and electrogenic cotransport of proline and sodium ions with a stoichiometry of 1:1. May have a role as transporter for neurotransmitter precursors into neurons. In contrast to other members of the neurotransmitter transporter family, does not appear to be chloride-dependent; Solute carriers
SNAP23	9606.ENSP00000249647	Synaptosomal-associated protein 23; Essential component of the high affinity receptor for the general membrane fusion machinery and an important regulator of transport vesicle docking and fusion; Belongs to the SNAP-25 family
SNX1	9606.ENSP00000261889	Sorting nexin-1; Involved in several stages of intracellular trafficking. Interacts with membranes containing phosphatidylinositol 3- phosphate (PtdIns(3 P)) or phosphatidylinositol 3,5-bisphosphate (PtdIns(3,5)P2). Acts in part as component of the retromer membrane-deforming SNX-BAR subcomplex. The SNX-BAR retromer mediates retrograde transport of cargo proteins from endosomes to the trans-Golgi network (TGN) and is involved in endosome-to-plasma membrane transport for cargo protein recycling. The SNX-BAR subcomplex functions to deform the donor membrane into a tubular profile
SPTAN1	9606.ENSP00000361824	Spectrin alpha chain, non-erythrocytic 1; Fodrin, which seems to be involved in secretion, interacts with calmodulin in a calcium-dependent manner and is thus candidate for the calcium-dependent movement of the cytoskeleton at the membrane; EF-hand domain containing
SRPRA	9606.ENSP00000328023	Signal recognition particle receptor subunit alpha; Component of the SRP (signal recognition particle) receptor. Ensures, in conjunction with the signal recognition particle, the correct targeting of the nascent secretory proteins to the endoplasmic reticulum membrane system
STAMBP	9606.ENSP00000377633	STAM-binding protein; Zinc metalloprotease that specifically cleaves ‘Lys-63’- linked polyubiquitin chains. Does not cleave ‘Lys-48’-linked polyubiquitin chains (By similarity). Plays a role in signal transduction for cell growth and MYC induction mediated by IL-2 and GM-CSF. Potentiates BMP (bone morphogenetic protein) signaling by antagonizing the inhibitory action of SMAD6 and SMAD7. Has a key role in regulation of cell surface receptor-mediated endocytosis and ubiquitin-dependent sorting of receptors to lysosomes. Endosomal localization of STAMBP is required for efficient EGFR degradation
STEAP3	9606.ENSP00000376822	Metalloreductase STEAP3; Endosomal ferrireductase required for efficient transferrin-dependent iron uptake in erythroid cells. Participates in erythroid iron homeostasis by reducing Fe(3 + ) to Fe(2 + ). Can also reduce of Cu(2 + ) to Cu(1 + ), suggesting that it participates in copper homeostasis. Uses NADP(+) as acceptor. May play a role downstream of p53/TP53 to interface apoptosis and cell cycle progression. Indirectly involved in exosome secretion by facilitating the secretion of proteins such as TCTP; STEAP family
STIM1	9606.ENSP00000478059	Stromal interaction molecule 1; Plays a role in mediating store-operated Ca(2 + ) entry (SOCE), a Ca(2 + ) influx following depletion of intracellular Ca(2 + ) stores. Acts as Ca(2 + ) sensor in the endoplasmic reticulum via its EF-hand domain. Upon Ca(2 + ) depletion, translocates from the endoplasmic reticulum to the plasma membrane where it activates the Ca(2 + ) release-activated Ca(2 + ) (CRAC) channel subunit ORAI1. Involved in enamel formation. Activated following interaction with STIMATE, leading to promote STIM1 conformational switch; Sterile alpha motif domain containing
SUGT1	9606.ENSP00000367208	SGT1 homolog, MIS12 kinetochore complex assembly cochaperone; Protein SGT1 homolog; May play a role in ubiquitination and subsequent proteasomal degradation of target proteins
TACC1	9606.ENSP00000321703	Transforming acidic coiled-coil-containing protein 1; Likely involved in the processes that promote cell division prior to the formation of differentiated tissues
TMEM57	9606.ENSP00000363463	Macoilin 1; Plays a role in the regulation of neuronal activity
TMPO	9606.ENSP00000266732	Lamina-associated polypeptide 2, isoform alpha; May be involved in the structural organization of the nucleus and in the post-mitotic nuclear assembly. Plays an important role, together with LMNA, in the nuclear anchorage of RB1; Belongs to the LEM family
TOR1AIP1	9606.ENSP00000435365	Torsin-1A-interacting protein 1; Required for nuclear membrane integrity. Induces TOR1A and TOR1B ATPase activity and is required for their location on the nuclear membrane. Binds to A- and B-type lamins. Possible role in membrane attachment and assembly of the nuclear lamina
TTK	9606.ENSP00000358813	Serine/threonine-protein kinase ttk/mps1; Dual specificity protein kinase TTK; Phosphorylates proteins on serine, threonine, and tyrosine. Probably associated with cell proliferation. Essential for chromosome alignment by enhancing AURKB activity (via direct CDCA8 phosphorylation) at the centromere, and for the mitotic checkpoint
UBE2J1	9606.ENSP00000451261	Ubiquitin-conjugating enzyme E2 J1; Catalyzes the covalent attachment of ubiquitin to other proteins. Functions in the selective degradation of misfolded membrane proteins from the endoplasmic reticulum (ERAD); Belongs to the ubiquitin-conjugating enzyme family
UBIAD1	9606.ENSP00000366006	UbiA prenyltransferase domain-containing protein 1; Prenyltransferase that mediates the formation of menaquinone-4 (MK-4) and coenzyme Q10. MK-4 is a vitamin K2 isoform present at high concentrations in the brain, kidney and pancreas, and is required for endothelial cell development. Mediates the conversion of phylloquinone (PK) into MK-4, probably by cleaving the side chain of phylloquinone (PK) to release 2- methyl-1,4-naphthoquinone (menadione; K3) and then prenylating it with geranylgeranyl pyrophosphate (GGPP) to form MK-4.
USP6NL	9606.ENSP00000277575	USP6 N-terminal-like protein; Acts as a GTPase-activating protein for RAB5A and RAB43. Involved in receptor trafficking. In complex with EPS8 inhibits internalization of EGFR. Involved in retrograde transport from the endocytic pathway to the Golgi apparatus. Involved in the transport of Shiga toxin from early and recycling endosomes to the trans-Golgi network. Required for structural integrity of the Golgi complex
UTRN	9606.ENSP00000356515	Utrophin; May play a role in anchoring the cytoskeleton to the plasma membrane; Zinc fingers ZZ-type
VANGL1	9606.ENSP00000347672	VANGL planar cell polarity protein 1
VAPB	9606.ENSP00000417175	Vesicle-associated membrane protein-associated protein B/C; Participates in the endoplasmic reticulum unfolded protein response (UPR) by inducing ERN1/IRE1 activity. Involved in cellular calcium homeostasis regulation
VRK2	9606.ENSP00000408002	Serine/threonine-protein kinase VRK2; Serine/threonine kinase that regulates several signal transduction pathways. Isoform 1 modulates the stress response to hypoxia and cytokines, such as interleukin-1 beta (IL1B) and this is dependent on its interaction with MAPK8IP1, which assembles mitogen-activated protein kinase (MAPK) complexes. Inhibition of signal transmission mediated by the assembly of MAPK8IP1-MAPK complexes reduces JNK phosphorylation and JUN-dependent transcription. Phosphorylates ‘Thr-18’ of p53/TP53, histone H3, and may also phosphorylate MAPK8IP1
WDR45B	9606.ENSP00000376139	Wd repeat domain phosphoinositide-interacting protein 3; Component of the autophagy machinery that controls the major intracellular degradation process by which cytoplasmic materials are packaged into autophagosomes and delivered to lysosomes for degradation. Binds phosphatidylinositol 3-phosphate (PtdIns3P) forming on membranes of the endoplasmic reticulum upon activation of the upstream ULK1 and PI3 kinases and is recruited at phagophore assembly sites where it regulates the elongation of nascent phagophores downstream of WIPI2
WIPI2	9606.ENSP00000288828	WD repeat domain phosphoinositide-interacting protein 2; Early component of the autophagy machinery being involved in formation of preautophagosomal structures and their maturation into mature phagosomes in response to phosphatidylinositol 3-phosphate (PtdIns3P). May bind PtdIns3P
WWOX	9606.ENSP00000457230	WW domain-containing oxidoreductase; Putative oxidoreductase. Acts as a tumor suppressor and plays a role in apoptosis. Required for normal bone development (By similarity). May function synergistically with p53/TP53 to control genotoxic stress-induced cell death. Plays a role in TGFB1 signaling and TGFB1-mediated cell death. May also play a role in tumor necrosis factor (TNF)-mediated cell death. Inhibits Wnt signaling, probably by sequestering DVL2 in the cytoplasm; Short chain dehydrogenase/reductase superfamily
YKT6	9606.ENSP00000223369	Synaptobrevin homolog YKT6; Vesicular soluble NSF attachment protein receptor (v- SNARE) mediating vesicle docking and fusion to a specific acceptor cellular compartment. Functions in endoplasmic reticulum to Golgi transport; as part of a SNARE complex composed of GOSR1, GOSR2 and STX5. Functions in early/recycling endosome to TGN transport; as part of a SNARE complex composed of BET1L, GOSR1 and STX5. Has a S-palmitoyl transferase activity; SNAREs
ZC3HAV1	9606.ENSP00000242351	Zinc finger CCCH-type antiviral protein 1; Antiviral protein which inhibits the replication of viruses by recruiting the cellular RNA degradation machineries to degrade the viral mRNAs. Binds to a ZAP-responsive element (ZRE) present in the target viral mRNA, recruits cellular poly(A)- specific ribonuclease PARN to remove the poly(A) tail, and the 3’- 5’ exoribonuclease complex exosome to degrade the RNA body from the 3’-end. It also recruits the decapping complex DCP1-DCP2 through RNA helicase p72 (DDX17) to remove the cap structure of the viral mRNA to initiate its degradation
ZDHHC5	9606.ENSP00000287169	Palmitoyltransferase ZDHHC5; Palmitoyl acyltransferase for the G-protein coupled receptor SSTR5. Also palmitoylates FLOT2 (By similarity); Zinc fingers DHHC-type
ZFYVE16	9606.ENSP00000337159	Mad, mothers against decapentaplegic interacting protein; Zinc finger FYVE domain-containing protein 16; May be involved in regulating membrane trafficking in the endosomal pathway. Overexpression induces endosome aggregation. Required to target TOM1 to endosomes; Protein phosphatase 1 regulatory subunits
ZFYVE9	9606.ENSP00000287727	Mad, mothers against decapentaplegic interacting protein; Zinc finger FYVE domain-containing protein 9; Early endosomal protein that functions to recruit SMAD2/SMAD3 to intracellular membranes and to the TGF-beta receptor. Plays a significant role in TGF-mediated signaling by regulating the subcellular location of SMAD2 and SMAD3 and modulating the transcriptional activity of the SMAD3/SMAD4 complex. Possibly associated with TGF-beta receptor internalization; Zinc fingers FYVE-type

Prepared using https://string-db.org/.

None of the 122 proteins displayed enrichment in the biotin conditions of the BioID-only samples or the non-transfected HEK293 cells (see heatmap in Fig. 6). This indicates that these proteins underwent biotinylation due to their proximity to LRRC8A when fused with BirA*. Notably, the LRRC8A-associated proteins comprise almost all the known elements of the VRAC complex (LRRC8B/C/D). The lack of the LRRC8E subunit poses no issue, as only LRRC8A is necessary for channel function and must bind with at least one other isoform [10, 13, 17, 18, 42]. Additionally, certain results match with details labelled in GeneMANIA, a versatile and user-friendly online platform for hypothesizing about gene function, scrutinizing gene catalogues, and prioritizing genes for functional assays. LRRC8A has been found to be functionally associated with USP6NL, LBR, SH3D19, and TMPO, as reported in [43], where the authors combine publicly available hybrid radiation datasets from different species to provide a highly reliable map of mammalian genetic interactions. Additionally, we discovered several unforeseen protein interactors, including STIM1, PTPN1, PTPN13, and RICTOR.

**Fig. 7:**
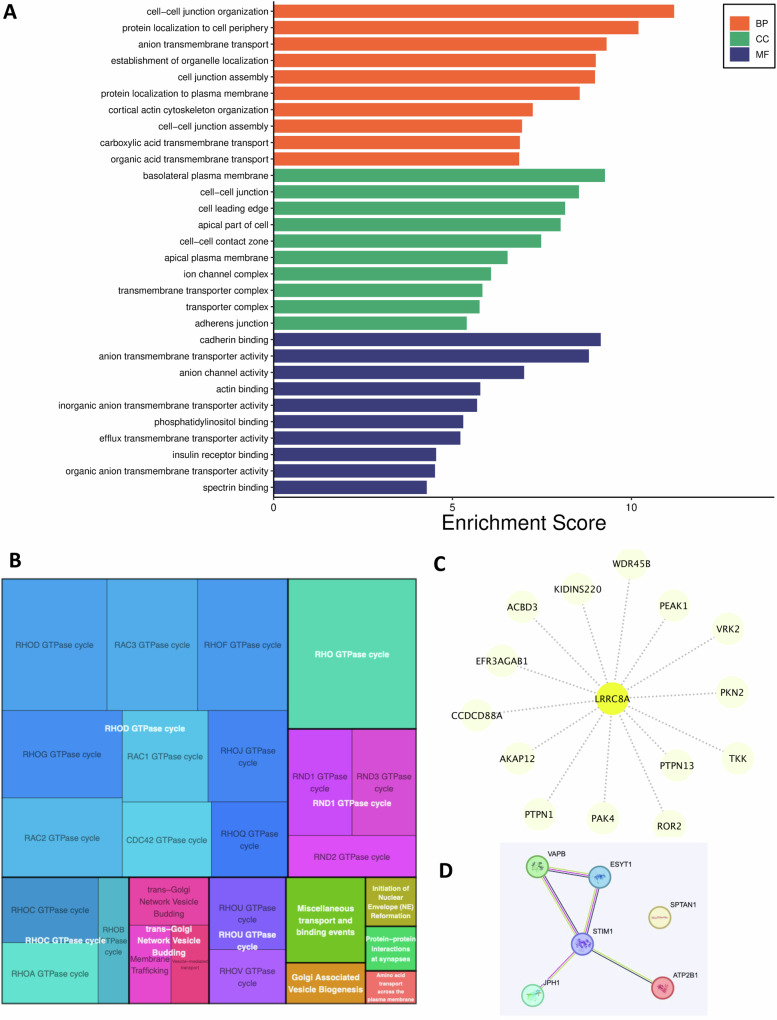
Pathway analysis of the interacting proteins. **A** Analysis of GO-term enrichment, showcasing the top 10 significant biological processes, molecular functions, and cellular components, respectively, derived from the 120 hit proteins. **B** Reactome pathway enrichment FDR < 0.05. **C** Cell-cell junctions exhibited the greatest enrichment in cellular components. The chart explores the range of cell-cell junction structures that contributed to this increased enrichment. **D** Schematic representation of calcium-related proteins identified using BioID. The figure was created using https://string-db.org.

To examine the findings of BioID, we implemented gene set analysis in accordance with the designated methodology from the Methods section. Subsequently, we conducted pathway enrichment analysis by utilizing GO and Pathway Reactome (PR) on the compilation of results from BioID. The figures exhibiting the ten most considerably enriched terms (*p* < 0.05) in each grouping are illustrated in Fig. 7A. The biological processes highlighted here include the cell-cell junctions organization, protein localization to cell periphery, anion transmembrane transport, establishment of organelle localization and cell junction assembly. Notably, the molecular functions identified in the GO terms, in addition to anion transport, comprise of phosphatidylinositol and insulin receptor binding. The cellular components with the highest enrichment were cell-cell junction, leading edge of the cell, actin cytoskeleton, cell-substrate junction, focal adhesion, cell cortex and basal plasma membrane. The list of cell-cell junction organisations includes CTNND1, DLG1, DSG2, EPB41L3, FERMT2, GJA1, LSR, NUMB, NUMBL, OCLN and PKN2 as shown in Fig. 7C. Through our analysis of PR enrichment (FDR < 0.05), we also observed physical proximity of LRRC8A to proteins associated with Rho subfamilies (Fig. 7B). Moreover, our BioID investigation indicates a prospective correlation between the VRAC channel and the management of cellular calcium. This involves proteins such as ATP2B1, ESYT1, JPH1, STIM1, SPTAN1, and VAPB (Fig. 7D).

## Discussion

Numerous investigations have examined the correlation between ion channels and cancer, suggesting their prospective application as targets for oncological therapies [4]. This paper presents a comprehensive outlook of publicly accessible databases addressing VRAC to diverse cancer types. Our meta-analysis infers a conceivable association between LRRC8A - the essential VRAC subunit- expression, and the survival of patients with cancer. Gene modifications of LRRC8A were examined across various tumour types (Fig. 1A–F). The LRRC8A mutation frequency is highest in UCEC, STAD, SKCM, SARC, and ESCA. However, the Kaplan-Meier analysis indicates a correlation between high LRRC8A expression and reduced survival rates only in COAD, HNSC, and PAAD patients (Fig. 1D). In HNSC, there is considerable evidence to suggest that VRAC function plays a critical role in mediating cisplatin sensitivity. Additionally, a molecular network analysis of LRRC8A has identified interactions with oncological factors, including HDAC4, NOTCH, and SNAI1 [44–48]. In PAAD, LRRC8A is associated with cell proliferation, migration, drug resistance, and immune infiltration [9]. Moreover, investigation of the LRRC8A gene’s co-expression and its network in PAAD patients has revealed prominent enrichment of genes situated close to LRRC8A in the PI3K-AKT and focal adhesion pathways [9]. On the contrary, detailed functions and interactions of VRAC in colon cancer are not yet clear and subjected of discussion. Some findings suggest that LRRC8A could serve as a novel biomarker for predicting the survival of colon cancer patients and that is a central mediator in mediating multiple signaling pathways to promote metastasis and targeting LRRC8A. LRRC8A proteins were found highly expressed in hematogenous metastasis from human colorectal cancer samples. The oxaliplatin-resistant HCT116 cells highly expressed LRRC8A, which was related to impaired proliferation and enhanced migration. The over-expressed LRRC8A slowed proliferation and increased migration ex vivo and in vivo [36]. Very recently, LRRC8A proteins were found highly expressed in hematogenous metastasis from human colorectal cancer samples. The elevated LRRC8A upregulated the focal adhesion, MAPK, AMPK, and chemokine signaling pathways via phosphorylation and dephosphorylation. Inhibition of LRRC8A impeded the TNF-α signaling cascade and TNF-α-induced migration. LRRC8A binding to PIP5K1B regulated the PIP2 formation, providing a platform for LRRC8A to mediate cell signaling transduction. Importantly, LRRC8A self- regulated its transcription via NF-κB1 and NF-κB2 pathways and the upregulation of NIK/NF-κB2/LRRC8A transcriptional axis was unfavorable for colon cancer patients [49]. However, in 2019, Liu et al. indicated that VRAC is not essential for the proliferation and migration of human colon cancer cells [37]. In this manuscript, we focused on a human CRC line derived from an adult male (HCT116 cells). Our independent clones of HCT116 LRRC8A-KO exhibited decreased proliferation rates and colony formation. However, the role in cell migration appears to be insignificant (Fig. 2A–C)

For further information in CRC context, we carried out an RNA-Seq analysis to evaluate the effects of LRRC8A deletion on the behaviour of HCT116 cells (Table 1). In the list of down-regulated genes, we found, amongst others, VSNL1, HNF4α, FOXA2, and KLF7. VSNL1 is a member of the neuronal calcium sensor protein family that controls calcium-dependent cell signalling and signal transduction by modulating adenylyl cyclase expression in a cAMP-dependent manner [50]. The precise functions of VSNL-1 have not been elucidated, but it appears to have several roles in tumour invasion and metastasis. Akagi et al. [51], using mRNA microarrays, evaluated the expression of VSNL-1 in >100 colorectal cancer patients. They observed that, compared to low VSNL-1 expression, high VSNL-1 expression was significantly associated with a high rate of lymph node metastases and poor prognosis for patients. It has been reported that down-regulation of VSNL1 inhibits the proliferation, migration, and invasion of colorectal cancer cells. Co-IP experiments indicated that VSNL1 can bind to COL10A1, which, when up-regulated, can promote colorectal cell proliferation, migration and invasion and reverse the effect of sh-VSNL1 on colorectal cancer cells [52]. Similarly, KLFs have been associated with the development of some cancers. Overexpression of KLF7 has been associated with worse prognosis in gastric and lung cancers, according to recent studies [53, 54]. However, mRNA expression levels of KLF7 have also been reported to be significantly elevated in CRC tissues [55]. In the list of down-regulated genes, we also found IL-18. It is known that the exit of Cl^-^ ions from immune cells activates the NLRP3 inflammasome. Green et al. [56] found that, in macrophages, VRACs are the only Cl^-^ channels involved in NLRP3 inflammasome activation when the cell volume changes.

In the list of up-regulated genes, we found: CCN3, CLMP, ILDR2, NECTIN4, DPP4, GJA3, and EZR. In particular, CCN3, a scaffolding protein that controls and balances the interconnection between individual signalling pathways, is involved in numerous biological processes that promote cancer development. CCN3 has antitumour effects in many tumours including CRC [57–61].

Although proteins are single entities, they hardly carry out their biological functions independently. Instead, they combine and create complex and dynamic molecular machines. A global map of PPIs within cellular systems offers fundamental understandings into the functioning of a specific protein. One of the main objectives of biological research has been the analysis and comprehension of molecular interactions within cells. Constructing maps that work out not only direct, binary protein-protein interactions but also incorporate information on indirect interactions is a significant challenge; these interactions include those between proteins that do not directly interact with each other but are part of the same multiprotein complex and proximal protein networks. To identify interactomes, methods must also consider the dynamic nature of various cellular processes and overcome the technical challenges presented by weak protein-protein interactions. The present study employs the innovative BioID proximal-labeling technique to disclose the extensive interactome of LRRC8A in human cells. The reliable cellular localization of biotinylated proteins was validated using LRRC8A-BioID in HEK293 cells before performing MS analysis (Fig. 5). By conducting three independent MS runs, approximately 120 proteins were identified, which represent around 10% of all proteins. To determine the significance of the BioID results, enrichment analyses were conducted using GO and PR (Figs. 6 and 7).

Although BioID and RNA-Seq are distinct in their focus, with BioID focusing on immediate protein interactions and RNA-Seq on gene expression dynamics, the combined results of these methods enrich our understanding of LRRC8A’s intricate role. LRRC8A has been identified as a key regulatory factor in crucial cellular pathways, as illustrated in Figs. 3 and 5. These findings are relevant in the context of colorectal carcinoma, where LRRC8A may regulate mechanisms impacting intercellular communication, metabolic signaling, and cellular stress responses. Disrupted calcium homeostasis could facilitate tumor progression. Recently, it has been revealed that LRRC8A is one of the components of the exosomes released from colon cancer HCT116 cells. Exosomes play a crucial role in intercellular communications within the microscopic tumor environment, facilitating the progression of colon cancer. Recently, it has been revealed that LRRC8A is one of the components of the exosomes released from colon cancer HCT116 cells. Exosomes play a crucial role in intercellular communications within the microscopic tumor environment, facilitating the progression of colon cancer [62]. Additionally, compromised cellular junctions might enhance metastatic potential, leading to a more aggressive and invasive tumor phenotype. Our research also examines hyperinsulinemia, which is prevalent in colorectal cancer. This condition could worsen tumor growth by promoting cellular proliferation and inhibiting apoptosis. Additionally, compromised cellular junctions might enhance metastatic potential, leading to a more aggressive and invasive tumor phenotype. Our research also touches on hyperinsulinemia, prevalent in colorectal cancer, which could exacerbate tumor growth by promoting cellular proliferation and inhibiting apoptosis. The calcium pathway, including IP3R, is implicated in resistance to cell destruction, including evasion of NK cell-mediated killing, positioning it as a possible therapeutic target.

EZR, a member of the ERM protein family, is a common component in RNAseq and BioID. Our data shows that EZR interacts with LRRC8A, and its expression is upregulated in KO cells (Table 3 and Fig. 3D). EZR acts as a linker connecting the actin cytoskeleton to the plasma membrane. The interaction is essential for maintaining cell shape, polarity, and surface structure integrity. EZR participates in several signaling pathways that regulate cell survival, proliferation, and motility [63]. It is involved in the Rho signaling pathway, which impacts actin filament assembly and disassembly, essential for cell movement and structural integrity. EZR also interacts with proteins such as PI3K (phosphoinositide 3-kinase), influencing pathways that control cell growth and survival. EZR interacts with various other cellular proteins, including CD44, a cell-surface glycoprotein involved in cell-cell interactions, cell adhesion, and migration. It interacts with membrane proteins such as ICAM-1 and VCAM-1, which are involved in leukocyte trafficking and immune responses. [64–69]. According to data from The Cancer Genome Atlas (TCGA), EZR expression is significantly decreased in colon cancer, with downregulation observed in 78% of colon adenocarcinoma (COAD) cases and 25% of rectal adenocarcinoma (READ) cases. In our models with LRRC8A KO cells, we observed an upregulation of EZR. This increase may indicate a compensatory mechanism in response to the downregulation of EZR observed in COAD. This could reflect adaptive changes in the cellular environment, or the activation of specific pathways triggered by the absence of LRRC8A.

Additionally, we have conducted an in-depth analysis of long non-coding RNAs (lncRNAs), which are increasingly recognized for their critical roles in cancer biology. LncRNAs, which do not produce proteins, play a crucial role in modulating gene expression and influencing key cellular processes such as growth, survival, and metastasis. They can function as either oncogenes or tumor suppressors. KLRL1-AS1 (ENSG00000245648) and CASC19 (ENSG00000254166), both down-regulated, exhibit atypical expression patterns in colon cancer, indicating significant roles in its pathology (Table 2 and Fig. 4A). KLRL1-AS1 is typically upregulated in colorectal cancer (CRC) cells. It affects cell proliferation by influencing the cell cycle and DNA damage response under TP53 regulation. Koldo Garcia-Etxebarria et al. identified a critical SNP within KLRL1-AS1 (rs10845123) that correlates with CRC prognosis and impacts five-year patient survival rates. [70]. Conversely, Wang et al. suggest that CASC19 acts as an oncogene in CRC progression, potentially serving as a valuable biomarker for CRC diagnosis and treatment [71]. This aligns with our findings where CASC19’s down-regulation in LRRC8A-KO cells is associated with decreased tumor growth. Additionally, the lncRNA ENSG00000250337, known as PURPL (a p53 level regulator increased by p53), exhibits up-regulation in LRRC8A-KO cells (Table 2 and Fig. 4B). This finding is particularly compelling as it contradicts studies by Li et al., who showed that depleting PURPL in colorectal cancer cells increases basal p53 levels and hinders growth, indicating that in its absence, MYBBP1A more effectively stabilizes p53 [72]. This complex interaction may not play a significant role in the reduced tumor growth observed in LRRC8A-KO cells [73, 74]. Also, two novel lncRNAs, ENSG00000277991 and ENSG00000258927, were found to be up-regulated (Table 2 and Fig. 4B). It is worth noting that ENSG00000258927 has only been studied in the context of ovarian cancer and is a newly discovered, uncatalogued lncRNA with limited information on its expression [73, 74].

Considering the limited information available on non-coding RNAs, these findings are significant and could be a valuable focus for further bioinformatic investigations using TCGA databases or other RNA sequencing analyses in the field of colorectal cancer. Additionally, these two lncRNAs may have the potential to serve as markers for identification, prognosis, or even therapeutic targeting in colorectal cancer.

Interestingly, among the LRRC8A interacting proteins, more than 40 have previously been found to interact with the LRRC8A ancestor, PANX1 [75]. Among these, ACBD3, CTNND1, DLG1, FERMT2, NUMB and NUMBL are common proteins found in the cell-cell junction that have been identified. DLG1 is a MAGUK scaffolding protein that regulates the localization and function of multiple ion channels [76, 77]. We also identified three proteins belonging to the 4.1 family: EPB41, EPB41L2 and EPB41L3. These proteins have essential functions in the assembly and maintenance of specific transmembrane protein complexes in the plasma membrane [78, 79]. 4.1 R performs a pivotal function in preserving the regularity of cell structure by bridging the spectrin-actin cytoskeleton to plasma membrane proteins and attracting DLG1 to the membranes. While hitherto, no research has linked VRAC to DLG1 or 4.1 R, it has been shown to associate with other channels such as NaV1.5, a sodium channel, and TRPC4, a non-selective cation channel that allows the transfer of calcium [80, 81].

Another group of significant interactors includes cadherin-binding proteins, such as AHNAK. The interaction between AHNAK and PANX1 was recently demonstrated using BioID techniques. The finding was further confirmed by co-immunoprecipitation, in combination with mass spectrometry [75]. The correlation between AHNAK and PANX1 led to initial insights into the mechanisms by which PANX1 suppresses malignant properties in rhabdomyosarcoma [75].

The dynamic mechanism of post-translational modification regulates several phases of the ion channel life cycle, such as maturation, trafficking, signalling and regulation [82–86]. Phosphorylation is thought to alter the probability of channel opening, gating, voltage dependence, desensitisation, and permeability. In the case of VRAC, post-translational modifications may focus on modifying the intracellular loop connecting the transmembrane pore to the leucine-rich repeat domain. This region contains numerous phosphorylation sites for amino acid residues such as serine, threonine, and tyrosine. Several studies suggest that PKC (protein kinase C) is a main regulator of VRAC activation [87–92]. Furthermore, a recent publication suggests that hypotonicity can significantly influence VRAC activation through the involvement of PKD [93]. Interestingly, AKAP12 and PKN2, two proteins related to PKC and PKA were identified in the list of interactors. AKAP12 controls the subcellular localisation of PKA and PKC through tethering. It can interact with these protein kinases and phosphatases to facilitate signalling pathways. PKN2 plays a critical role in the organisation of the actin cytoskeleton by activating Rho GTPase in coordination with PKC. We also found other kinase-related proteins, such as ACBD3, CCDCD88A, EFR3A, GAB1, PAK4, PEAK1, PTPN1, PTPN13, ROR2, KIDINS220, TKK, VRK2 and WDR45B (see Fig. 7D).

Analysing the Reactome pathways (FDR < 0.05), the physical proximity of LRRC8A to proteins linked to Rho subfamilies was found (Fig. 5E). Our BioID analysis results align with previous findings that suggest a potential association between intracellular GTPγS and VRAC, implying that the Rho pathway may regulate VRAC activation, as reported in [27, 94, 95]. Rho proteins are thought to shift between their active form (Rho-GTP) on the cell membrane to an inactive form (Rho-GDP) in the cytoplasm. Rho GTPases regulate various cellular processes that demand dynamic reorganization of the cytoskeleton, such as cell migration, adhesion, division, polarity establishment, and intracellular transport [96, 97]. Previous research has shown that intracellular application of GTPγS, a non-hydrolysable analogue of GTP, can activate VRAC currents, even in isotonic conditions [27, 28, 98]. In contrast, GDPβS, a non-hydrolysable analogue, results in the time-dependent inhibition of VRAC [99]. Although the role of GTPγS has been subject to extensive scrutiny, the current understanding of how GTPγS modulates VRAC signalling remains limited.

As mentioned previously, VRAC is an important pathway for anion transport during cell volume regulation. It is typically activated in response to cell swelling, but how the channel senses swelling remains unclear. Lemonnier and co-workers provided evidence for colocalization of VRAC with store-operated Ca^2+^ channels and showed that activation of VRAC is strongly dependent on Ca^2+^ release through IP3R [100]. They concluded that VRAC is regulated within Ca^2+^ microdomains. Similarly, Akita and collaborators found that activation is regulated by high concentration regions of intracellular Ca^2+^ in the immediate vicinity of open Ca^2+^-permeable channels, called Ca^2+^ nanodomains [101, 102]. However, Liu et al. showed that intracellular Ca^2+^ is necessary but not sufficient to activate LRRC8A-mediated currents [103]. Our BioID analysis suggests a potential link between the VRAC channel and the regulation of cellular calcium. The list includes ATP2B1, ESYT1, JPH1, STIM1, SPTAN1 and VAPB (Fig. 7F). Of particular interest is the correlation with STIM1. STIM1 is functionally related to the CRAC channel, allowing the influx of Ca^2+^ ions from the extracellular space into the cytosol upon depletion of stored Ca^2+^ ions in the ER [104]. After Ca^2+^ depletion, STIM1 forms oligomers and migrates to ER-PM junctions. The subsequent interaction of STIM1 with ORAI1 and ORAI2 causes the opening of the CRAC channels [105]. ATP2B1 is a plasma membrane ATP dependent calcium pump. It regulates insulin sensitivity through Ca^2+^/calmodulin signalling pathway by regulating AKT1 activation and NOS3 activation in endothelial cells [106]. ESYT1 binds Ca^2+^ and translocates to sites of contact between the endoplasmic reticulum and the plasma membrane in response to elevated cytosolic Ca^2+^ levels. It assists in the tethering of the ER to the plasma membrane [107]. Furthermore, JPH1 provides a structural foundation for functional crosstalk between the cell surface and intracellular Ca^2+^ release channels [108]. SPTAN1 is a protein with an EF-hand domain that appears to be involved in secretion, and it interacts with calmodulin in a Ca^2+^-dependent manner. This interaction allows it to move the cytoskeleton across the membrane [109].

## Conclusion

This study investigates the correlation between the expression levels of VRAC genes and patient survival across different cancer types. The research uncovers a noteworthy correlation between LRRC8A expression and prognosis in specific oncological contexts. Furthermore, RNA-Seq analysis was employed to examine DEGs in CRC cells that lack LRRC8A, providing novel insights into the genetic alterations caused by the absence of this ion channel. This analysis explores the LRRC8A interactome in living mammalian cells, providing insight into the molecular dynamics of VRAC.

The study of PPIs and gene regulatory systems is crucial for comprehending the genetic and molecular basis of diseases and advancing therapeutic developments. BioID is an effective method for identifying interactions, regardless of the solubility properties of the proteins involved. This technique is particularly useful for analysing proteins that are challenging to purify or have variable solubility, such as ion channels. However, it is essential to recognise the inherent limitations of BioID, like any experimental technique. Future validations will be necessary, but these initial findings are crucial as they represent some of the first focused explorations of the VRAC channel members. This data establishes the foundation for more detailed studies on the functions and interactions of VRAC channels. It highlights the importance of these early insights in the fields of cellular biology and physio/pathology.

## Materials and Methods

### Plasmid constructs

The wild-type human LRRC8A gene was subcloned from the “8a-pcdna3-hektor” plasmid provided by Dr. Raul Estevez into pcDNA3.1-MCS-BirA(R118G)-HA (Addgene #36047(Roux, Kim et al. 2012)) using primers that modified the stop codon and appended restriction enzyme sequences to the 5’ overhangs. The reaction made use of the following PCR primers: The reaction utilized the NheI forward (5′-aaagctagcaccatgattccggtgacagagctccgctac-3’) and EcoRI reverse (5’- tgcgaattctgcggccttcagccctccacag-3’) primers. The amplification was conducted using the Phusion enzyme (NEB England). Confirmation of successful LRRC8A-BirA* fusion protein-containing clones was assessed via sequencing (Eurofins Genomics).

### Cell cultures

The study used HEK293 cells and HCT116 cells (received as a gift from Prof. Szabò at the University of Padua). The cells were cultured in Dulbecco’s modified Eagle medium (DMEM) with 10% fetal bovine serum (FBS), 10 mM HEPES, 100 U/ml penicillin, and 100 U/ml streptomycin along with 1X non-essential amino acids (NAA) from Gibco. The cells were grown at 37 °C under a humidified atmosphere with 5% CO2. T25 flasks were used to culture the cells. Upon reaching 70 to 80% confluency, the cells were trypsinized and seeded in culture flasks or Petri dishes with a 10 to 20% density. Coverslips were included in the Petri dishes used for the experiments. For biotinylation assays, the cells were incubated for 24 hours at 37 °C in media with 50 μM of biotin, under 5% CO_2_.

### Survival analysis using Kaplan–Meier Plotter and GEPIA

The correlation between LRRC8A expression and clinical outcomes in cancer was evaluated through the analysis of Kaplan-Meier plots and survival analysis module available on SurvivalGenie (https://bbisr.shinyapps.winship.emory.edu/SurvivalGenie/, accessed on 10 November 2023). The PP-network of LRRC8A-E was analysed using the Search Tool for Retrieval of Interacting Genes/Proteins (https://string-db.org/, accessed on 28 August 2023) and GeneMANIA (http://www.genemania.org, accessed on 10 November 2023).

### Generation of HCT116 LRRC8A knockout cell lines using CRISPR/Cas9 technology

The CRISPR/CAS9 method employed the pSpCas9n(BB)-2A-Puro (PX459) plasmid (Addgene Plasmid #48139 Zhang Lab), according to the instructions provided by Ran et al. (2013). The sgRNA guides were designed specifically to remove exon 3. The following sgRNA sequences were utilized: HsLRR8A_u11 FOR CACCGGCTATCTGCGCGTCGGCTGT, HsLRR8A_u11 REV AAACACAGCCGACGCGCAGATAGCC, HsLRR8A_u12 FOR CACCGTGGCTCTGCTATCTGCGCGT, and HsLRR8A_u12 REV AAACACGCGCAGATAGCAGAGCCAC were used as sgRNA sequences. The following sgRNA sequences were utilized: HsLRR8A_d13 FOR CACCGCCTGGGGCCGCTTGTGAGTC, HsLRR8A_d13 REV AAACGACTCACAAGCGGCCCCAGGC, HsLRR8A_d14 FOR CACCGCCTGGCTGTCCGGGAGTTCT, and HsLRR8A_d14 REV AAACAGAACTCCCGGACAGCCAGGC were used as sgRNA sequences. The annealed and phosphorylated guides were connected to the vector at the BbsI (NEB) restriction site beneath the U6 promoter and authenticated through sequencing. A total of one hundred thousand HCT116 cells were cultured in a six-well plate. The next day, several pairs of single guides were employed to deliver 2.5 micrograms of plasmid DNA (1.25 μg per guide) to the HCT116 cells via the TransIT®-LT1 transfection reagent (Mirus). 48 hours post-transfection, the cell medium had puromycin at a concentration of 1.5 μg/ml (Gibco), which persisted for 96 hours. After selection, the cells were stepwise diluted to 0.5 cells per well in a 96-well plate to limit the presence of multiple cells in a single well. Actively growing cells were gathered and centrifuged at 600 g for 5 minutes. The medium was discarded, and the DNA was extracted from the pellet using a MyTaq Extract-PCR kit (Meridian Bioscience) in accordance with the manufacturer’s guidelines. The quantity was calculated using a Thermo Scientific ND2000 spectrophotometer.

### Evaluation of cell proliferation

25,000 HCT116 cells were seeded in 12-well plates. The cells were detached from the plates at 24-, 36-, 48- and 72-hours intervals using 100 μL Gibco trypsin and then diluted in 100 μL of medium. Cell proliferation was assessed daily by using the Logos Biosystem’s LUNA™II Automated Cell Counter.

### Evaluation of cell migration

The potential for cell migration was measured using wound-scratch experiments. HCT116 cells were seeded in 24-well culture plates and allowed to grow until they reached 80-90% confluence. A treatment of 5 μg/mL mitomycin C (Sigma Aldrich) was administered one hour before the scratch. A plastic 200 μl pipette tip was used to scratch the monolayer of cells. Following a wash in PBS, the medium was substituted with phenol red-free medium containing 5% FBS. The same fields were photographed at 0, 15, 20, and 24 hours after performing the scratch with a Leica DMI4000 inverted microscope. The area of the scratch was measured using the ImageJ software (NIH). The migration rate was quantified as a percentage of the initial scraped area.

### Colony formation assay

A total of six hundred HCT116 cells were cultured in a standard culture medium for 6 days using a 6-well plate. The medium was then discarded, and the cells were washed with PBS twice and then fixed with 3.8% paraformaldehyde for 30 minutes. After three more washes with PBS, the cells were stained with 0.1% crystal violet at room temperature for 15 minutes. The PBS was used to remove the staining until the colonies were cleared. At a magnification of 1 × 0.5, the Leica stereo microscope MZ16F was used to capture the images. The software used to identify the number and dimensions of the colonies was ImageJ (NIH).

### Quantification of apoptotic events

HCT116 cells were cultivated in DMEM medium supplemented with 10% FBS and seeded in a six-well plate at a density of 5 × 10^^5^ cells. After 24 hours, the cells were collected, washed with PBS, and centrifuged at 300 *g*. The cells were subsequently stained with 2.5 µl of annexin V and propidium iodide (PI) in binding buffer (Thermo Fisher) for 15 minutes in the dark at 4 °C to determine apoptotic events. The BD LSR Fortessa X-20 flow cytometer was employed to evaluate cell populations for annexin V-positive or double-positive cells. Histograms for APC-A, PE-A, SSC-A, and FSC-A were obtained.

### Cell Cycle Analysis

HCT116 cells were cultivated using DMEM medium supplemented with 10% FBS, and then cultured in a 6-well plate with a density of 5 × 10^^5^ cells. After 24 hours, the cells underwent cold PBS washing twice and were treated with 70% ethanol-based cell permeabilization at ice-cold temperatures for an hour. Following incubation at 37 °C with a solution containing 50 µg/ml propidium iodide (Sigma-Aldrich) and 10 ng/ml RNase A (Qiagen) for one hour, the cells underwent centrifugation at 1000 *g*/min, were washed with PBS, and analysed using BD LSR Fortessa X-20 flow cytometry. Following incubation at 37 °C with a solution containing 50 µg/ml PI (Sigma-Aldrich) and 10 ng/ml RNase A (Qiagen) for one hour, the cells underwent centrifugation at 1000 *g*/min, were washed with PBS, and analysed using BD LSR Fortessa X-20 flow cytometry. Cell cycle stages were determined, and quantification of PE-A, FSC-AA, and SSC-A histograms was performed using BD FACS Diva 9.0.

### RNA-seq analyses: alignment, pre-processing, and differential expression

HCT116 cells were seeded in a 6-well plate with a density of 0.4 × 10^6 cells for WT and 0.5 × 10^^6^ cells for KO. RNA was extracted using RNeasy Mini Kit (Qiagen) according to the recommended protocol. Reads were aligned to the reference genome with STAR (v 2.7.10a) [110] and quantified with RSEM (v1.3.1). The indexed genome was built with RSEM starting from Ensembl’s Homo Sapiens DNA primary assembly (release 106) [111]. To identify the differentially expressed genes we used the edgeR R package [112]. We provided as input the filtered raw counts with the design matrix defined by the dichotomous variables for the different clones. The RLE normalization was applied to the samples. False Discovery Rate (FDR) less than 0.01 was used to significantly select DEG.

The LncRNome analyses was performed using the Cancer LncRNome Atlas is a comprehensive database or resource that catalogs and characterizes long non-coding RNAs (lncRNAs) associated with various types of cancer (http://fcgportal.org/TCLA/search.php).

### Transfections and the generation of stable cell lines

The TransIT®-LT1 transfection reagent (Mirus) was used to transfect cells with LRRC8A-BirA* following the manufacturer’s recommended protocols for BioID. After transfection, HEK293 cells were selected with 750 μg/mL Geneticin (G418) to create stable cell lines. Monoclonal cells were isolated and grown following colony formation, and their stable expression was confirmed using Western blotting. Stable HEK293 cell lines were maintained using G418.

### Patch-clamp analyses

HEK-5X-KO lrrc8-/- cells used for patch clamp recordings were knock-out for all five genes encoding lrrc8 subunits [14] and were kindly provided by Thomas Jentsch (Berlin). Cells were cultured in DMEM (Pan Biotech) supplemented with 10% FBS, 1% penicillin/streptomycin and 1% glutamine and maintained at 37 °C in a 5% CO2, 100% humidity atmosphere. Cells were grown on plastic tissue culture dishes and splitted every 3–4 days. Cells were co-transfected using the effectene reagent (Qiagen) with the BirA-tagged LRRC8A plasmid and LRRC8E in pCDNA3.1 as in [92]. For the identification of positively transfected cells, a plasmid encoding the CD8 antigen was co-transfected. The transfected cells were identified by microbeads coated with anti-CD8 antibodies (Dynabeads M-450 CD 8; ThermoFisher) as described in [113].

Currents were recorded 24–36 h after transfection. The standard current-voltage protocol (IV) for stimulation consisted of 500 ms-long voltage steps ranging from -80 to 120 mV in 20 mV increments. Current response to the various stimuli were monitored using the “time course protocol”, which consisted of successive steps of 50 ms pulses to −75, −25, 0, 25, and 75 mV every 5 s.

The standard extracellular isotonic solution contained in mM: 145 NaCl, 6 KCl, 1.5 CaCl_2_, 1 MgCl_2_, 10 HEPES, 10 glucose (pH 7.4, 310 mOsm). Hypotonic solution contained in mM: 105 NaCl, 6 CsCl, 1.5 CaCl_2_, 1 MgCl_2_, 10 HEPES, 10 glucose (pH7.4, 230 mOsm). The standard pipette solution to monitor VRAC activation upon hypotonic perfusion contained (in mM) 100 K-Gluconate, 40 CsCl, 2 MgCl_2_, 1.9 CaCl_2_, 5 EGTA-NMDG,1 Na2ATP, and 10 HEPES-NMDG, pH 7.3 (290 mOsm).

### Immunofluorescence

The cells that were transfected with BioID constructs were fixed using 3.8% paraformaldehyde and permeabilized in 0.1% Triton-X100 in phosphate buffer saline (PBS) for five minutes. The coverslips were incubated with primary antibody, anti-HA (Abcam, 1:500), overnight. After washing in PBS, the coverslips were then incubated with streptavidin coupled with Alexa Fluor 488 (Invitrogen, 1:1000), and goat anti-rabbit 568 (Invitrogen, 1:500) secondary antibodies. The coverslips were washed three more times using PBS, then mounted on glass slides using ProLong Gold containing 4′,6′-diamidino-2-phenylindole (DAPI; Invitrogen). Images of the cells were captured using a 63x oil objective with a Leica SP5 confocal microscope (Leica Microsystem, Wetzlar, Germany).

### Membrane extraction

The ProteoExtract kit (Merck/Sigma-Aldrich) was used according to the recommended protocol to separate and collect soluble and membrane protein fractions.

### Immunoblotting

Whole-cell extracts were prepared using a lysis buffer composed of 25 mM Tris-HCl pH 7.8, 2.5 mM EDTA, 10% (v/v) glycerol, and 1% (v/v) NP40, supplemented with a Protease Inhibitor Cocktail (Sigma) and 2 mM DTT. The lysates were resolved by SDS-PAGE using 4-12% ExpressPlus® PAGE gels (GenScript) and then transferred to a membrane (polyvinylidene fluoride, PVDF, Amersham™Hybond™ P 0.45μm). The membranes were checked for equal loading using a Ponceau solution, followed by washing and blocking for one hour in 5% skim milk in Tris-buffered saline (TBS: 10 mM Tris, 150 mM NaCl, pH 7.4). The blotted proteins were detected by incubation overnight at 4 °C with the following antibodies after washing three times with TBS-T (TBS plus 0.05% Tween). All Western blots were developed using the Clarity Western ECL substrate (Bio-Rad) and then imaged on the ChemiDoc Imager (Bio-Rad). We used the following primary antibodies: Streptavidin-HPR (1:40.000, Thermofisher, #21130), anti-PMCA (1:2000, Invitrogen #MA3-914), anti-actin (1:2000, Millipore #MAB1501), anti-HA (1:1000, Abcam #AB9110), anti-LRRC8A (1:1000, Bethyl #A304-175A).

The detection of biotinylated proteins was conducted with specific adaptations tailored for nitrocellulose membranes. The detection of biotinylated proteins was carried out with the following modifications. After transfer, membranes (nitrocellulose, Amersham™Hybond™ P 0.45μm) were blocked for 30 minutes in 1% BSA in phosphate-buffered saline (PBS) containing 0.2% Triton X-100 and then incubated in the same buffer with HRP-conjugated streptavidin (Thermo Fisher Scientific, 21130, 1:40000) for 40 minutes.

### Streptavidin pull-down of biotinylated proteins

For MS analysis, large-scale pull-downs were conducted by seeding 1 × 106 cells expressing BioID-LRRC8A fusion proteins on four 10-cm plates. The cells were grown to 70–80% confluence and then incubated in complete media with 50 μM biotin for 24 hours. Cells were washed with PBS and lysed in RIPA lysis buffer (50 mM Tris-HCl pH 7.5, 150 mM NaCl, 1 mM EDTA, 1% (v/v) NP-40, 0.1% (w/v) SDS, 0.5% (w/v) sodium deoxycholate, 1 mM DTT, and protease inhibitors) at room temperature. The lysate was then incubated on ice for 15 minutes, as described in a previous study [114]. The cell lysate samples were centrifuged at 13000 × *g* for 10 minutes at 4 °C. The supernatant underwent Streptavidin-based pull-down using MyOneDynabeads Streptavidin C1 (Thermo Fisher Scientific), following the method outlined in [115].

To test for protein biotinylation, we reserved around 10% of the whole sample specifically for Western blot analysis. The proteins were extracted from the beads by boiling them with SDS for 5 minutes at 95 °C. The remaining sample was used for MS analysis.

### Mass spectrometry

The raw IsobarQuant output files (protein.txt – files) were processed using the R programming language (www.r-project.org). Only proteins that were quantified with at least two unique peptides were considered for analysis. Raw TMT reporter ion intensities (‘signal_sum’ columns) were first cleaned for batch effects using limma [116] and further normalized using vsn (variance stabilization normalization [117]). The differential expression of the proteins was tested using the limma package. The replicate information was added as a factor in the design matrix given as an argument for the limma ‘lmFit’ function. 122 potentially interacting proteins were defined as proteins with significant change (fdr <= 0.05 and fold-change >= 2) in comparisons ‘LRRC8A-BirA*-HA#1 Biotin vs LRRC8A-BirA*-HA#1 noBiotin’ or ‘LRRC8A-BirA*-HA#2 Biotin vs LRRC8A-BirA*-HA#2 noBiotin’.

### LC-MS/MS acquisition

An UltiMate 3000 RSLC nano LC system (Dionex) was used, which had a trapping cartridge (µ-Precolumn C18 PepMap 100, 5 µm, 300 µm i.d. x 5 mm, 100 Å) and an analytical column (nanoEase™ M/Z HSS T3 column 75 µm x 250 mm C18, 1.8 µm, 100 Å, Waters). The trapping was performed for 6 minutes using a constant trapping solution flow of 0.05% trifluoroacetic acid in water at a rate of 30 µL/min on the trapping column. The peptides were then eluted using solvent A (0.1% formic acid in water, 3% DMSO) with a constant flow rate of 0.3 µL/min through the analytical column. The proportion of solvent B (0.1% formic acid in acetonitrile, 3% DMSO) was gradually increased during this process. The outlet of the analytical column was directly connected to an Orbitrap Fusion™ Lumos™ Tribrid™ Mass Spectrometer (Thermo Fisher) utilizing the Nanospray Flex™ ion source in the positive ion mode.

The peptides were received by the Fusion Lumos using a Pico-Tip Emitter with a 10 µm tip a diameter of 360 µm and an inner diameter of 20 µm (New Objective) while applying a spray voltage of 2.4 kV. The capillary temperature was maintained at 275 °C. The whole mass scan was obtained in the orbitrap with a resolution of 120,000 in profile mode. Mass ranges from 375 to 1500 m/z were employed. The filling time was restricted to a maximum of 50 ms and no more than 4 × 105 ions. Data-dependent acquisition (DDA) with a fill time of 94 ms and a limitation of 1 × 105 ions was carried out. Orbitrap’s resolution was set at 30,000. We used a collision energy of 38 in normalized units. We acquired MS2 data in profile mode.

### MS data analysis – Isobarquant

IsobarQuant and Mascot (v2.2.07) were used to process the acquired data, which were searched against a Uniprot Homo sapiens proteome database (UP000005640) containing common contaminants and reversed sequences. The following modifications were included in the search parameters Carbamidomethyl (C) and TMT11 (K) (fixed modification), Acetyl (protein N-terminal), Oxidation (M) and TMT11 (N-terminal) (variable modifications). A mass error tolerance of 10 ppm was set for the full scan (MS1) and 0.02 Da for the MS/MS spectra (MS2). Other parameters were: trypsin as protease with a maximum of two missed cleavages allowed; a minimum peptide length of seven amino acids; at least two unique peptides were required for protein identification. The false discovery rate at the peptide and protein level was set at 0.01.

### Mass spectrometry data analysis

The raw IsobarQuant output files (protein.txt – files) were processed using the R programming language (www.r-project.org). Only proteins that were quantified with at least two unique peptides and identified in all mass spec runs were considered for analysis. Raw reporter ion intensities (signal_sum columns) were first cleaned for batch effects using limma [116] and further normalized using vsn (variance stabilization normalization [117]. Missing values were imputed with the ‘knn’ method using the Msnbase package [118]. The differential expression of the proteins was tested using the limma package. The replicate information was added as a factor in the design matrix given as an argument for the limma lmFit function. Furthermore, the imputed values were given a weight of 0.05 in the ‘lmFit’ function. A protein was annotated as a hit with a false discovery rate (FDR) smaller than 5% and a fold change of at least 100% and as a candidate with an FDR below 20% and a fold-change (FC) of at least 50%.

### Bioinformatic analysis

We used the list of selected proteins to identify significantly enriched functional categories. We conducted enrichment analyses using the clusterprofiler R package (Wu, Hu et al., 2021) on the Gene Ontology (GO) categories of biological process (BP), molecular function (MF), and cellular component (CC), as well as the Reactome and KEGG pathway databases [119]. We employed the False Discovery Rate (FDR) to control for multiple testing. We identified significantly enriched GO terms and Reactome pathways using a false discovery rate (FDR) threshold of 0.05. To reduce the redundancy of significant GO terms, we employed semantic similarity distance, which is implemented in the R package rrvo (https://ssayols.github.io/rrvgo). The findings were graphically summarized using dot, scatter, and tree plots. The maps that displayed significantly enriched KEGG pathways were color-coded according to the logarithmic fold change (logFC) of the proteins.

### Statistical analysis

The data is presented as means ± standard error of the mean. Data analysis was performed using the GraphPad Prism 8 software. Two-way ANOVA with Bonferroni’s test was used for comparing the data due to two variables. We compared two groups using the unpaired Student’s t-test. Additional statistical information is available in the figure legends.

### Supplementary information

Figure Supplementary S1

Original blots (uncropped)

## Data Availability

The datasets used in the current study are available from the corresponding authors on reasonable request.
