# Publisher Correction: Interactomic exploration of LRRC8A in volume-regulated anion channels

**DOI:** 10.1038/s41420-024-02123-y

**Published:** 2024-08-29

**Authors:** Veronica Carpanese, Margherita Festa, Elena Prosdocimi, Magdalena Bachmann, Soha Sadeghi, Sara Bertelli, Frank Stein, Angelo Velle, Mostafa A. L. Abdel-Salam, Chiara Romualdi, Michael Pusch, Vanessa Checchetto

**Affiliations:** 1 DiBio, Unipd, via Ugo Bassi 58/B, 35131 Padova, Italy; 2 Institute of Biophysics, CNR, Via De Marini, 6, 16149 Genova, Italy; 3 Proteomics Core Facility, EMBL Heidelberg, Meyerhofstraße 1, 69117 Heidelberg, Germany; 4 Padua Center for Network Medicine, University of Padua, Via F. Marzolo 8, 315126 Padova, Italy; 5 RAISE Ecosystem, Genova, Italy; 6 Present Address: Institute of Biophysics, CNR, Via De Marini, 6, 16149 Genova, Italy; 7 Present Address: Daba Farber Cancer Research Institute, Boston, MA USA; 8 Present Address: Humboldt Universität Berlin, AG Zelluläre Biophysik, Dorotheenstr, 19-21 10099 Berlin, Germany; 9 Present Address: UPMC Hillman Cancer Center, Pittsburgh, PA USA

**Keywords:** Chloride channels, Protein-protein interaction networks

Correction to: *Cell Death Discovery* 10.1038/s41420-024-02032-0, published online 22 June 2024

In this article, Fig. 7 and Table 3 have been corrected.

Old Fig. 7
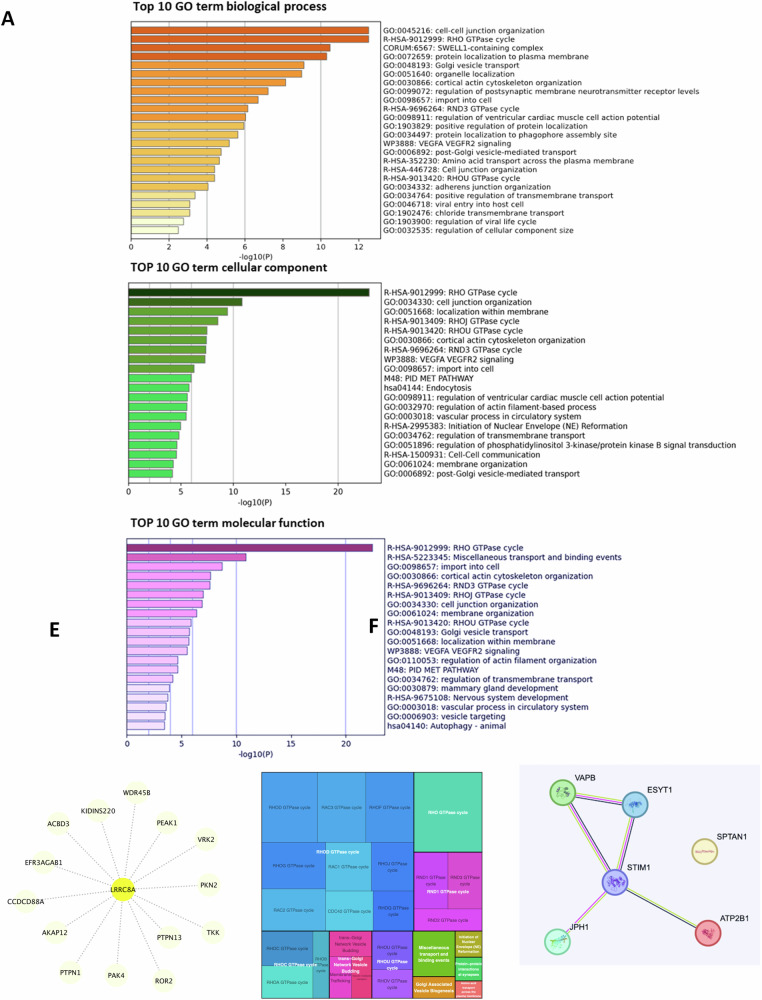


New Fig. 7
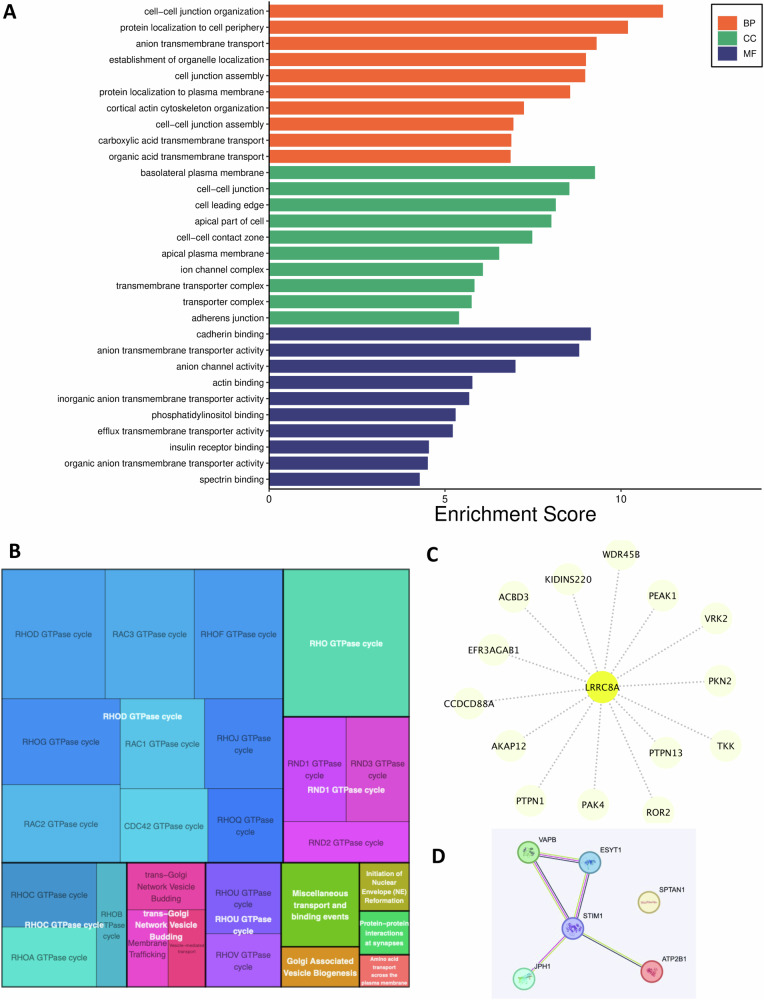


Old Table 3

**Table 3:** List and annotations of hit proteins from BioID analyses. Prepared using.

Protein	stringID	Annotation
ABCC5	9606.ENSP00000333926	Multidrug resistance-associated protein 5; Acts as a multispecific organic anion pump that can transport nucleotide analogs; Belongs to the ABC transporter superfamily. ABCC family. Conjugate transporter (TC 3.A.1.208) subfamily
ABR	9606.ENSP00000303909	Active breakpoint cluster region-related protein; GTPase-activating protein for RAC and CDC42. Promotes the exchange of RAC or CDC42-bound GDP by GTP, thereby activating them; C2 domain containing
ACBD3	9606.ENSP00000355777	Acyl-coa-binding domain containing 3; Golgi resident protein GCP60; Involved in the maintenance of Golgi structure by interacting with giantin, affecting protein transport between the endoplasmic reticulum and Golgi. Involved in hormone-induced steroid biosynthesis in testicular Leydig cells (by similarity). Recruits PI4KB to the Golgi apparatus membrane; enhances the enzyme activity of PI4KB activity via its membrane recruitment, thereby increasing the local concentration of the substrate in the vicinity of the kinase; A-kinase anchoring proteins
ADD2	9606.ENSP00000264436	Beta-adducin; Membrane-cytoskeleton-associated protein that promotes the assembly of the spectrin–actin network. Binds to the erythrocyte membrane receptor SLC2A1/GLUT1 and may, therefore, provide a link between the spectrin cytoskeleton to the plasma membrane. Binds to calmodulin. Calmodulin binds preferentially to the beta subunit; Belongs to the aldolase class II family.
ADD3	9606.ENSP00000348381	Gamma-adducin; Membrane-cytoskeleton-associated protein that promotes the assembly of the spectrin–actin network. Plays a role in actin filament capping. Binds to calmodulin; Belongs to the aldolase class II family. Adducin subfamily
AHNAK	9606.ENSP00000367263	Neuroblast differentiation-associated protein AHNAK; May be required for neuronal cell differentiation; PDZ domain-containing
AKAP12	9606.ENSP00000384537	A-kinase anchor protein 12; Anchoring protein that mediates the subcellular compartmentation of protein kinase A (PKA) and protein kinase C (PKC); A-kinase anchoring proteins
ALDH3A2	9606.ENSP00000345774	Aldehyde dehydrogenase 3 family member a2; Fatty aldehyde dehydrogenase; Catalyzes the oxidation of long-chain aliphatic aldehydes to fatty acids. Active on a variety of saturated and unsaturated aliphatic aldehydes between 6 and 24 carbons in length. Responsible for conversion of the sphingosine 1-phosphate (S1P) degradation product hexadecenal to hexadecenoic acid
ANKS1A	9606.ENSP00000353518	Ankyrin repeat and SAM domain-containing protein 1A; Regulator of different signaling pathways. Regulates EPHA8 receptor tyrosine kinase signaling to control cell migration and neurite retraction (By similarity); Ankyrin repeat domain-containing
ARHGAP21	9606.ENSP00000379709	Rho GTPase-activating protein 21; Functions as a GTPase-activating protein (GAP) for RHOA and CDC42. Downstream partner of ARF1, which may control Golgi apparatus structure and function. Also required for CTNNA1 recruitment to adherens junctions; PDZ domain-containing
ARHGAP39	9606.ENSP00000366522	Rho gtpase-activating protein 39; Rho GTPase-activating protein 39
ATP2B1	9606.ENSP00000392043	Plasma membrane calcium-transporting ATPase 1; This magnesium-dependent enzyme catalyzes the hydrolysis of ATP coupled with the transport of calcium out of the cell; ATPases Ca^2+^ transporting
CCDC88A	9606.ENSP00000338728	Coiled-coil domain-containing 88a; Girdin; Plays a role as a key modulator of the AKT-mTOR signaling pathway controlling the tempo of the process of newborn neuron integration during adult neurogenesis, including correct neuron positioning, dendritic development, and synapse formation (by similarity). Enhances phosphoinositide 3-kinase (PI3K)-dependent phosphorylation and kinase activity of AKT1/PKB, but does not possess kinase activity itself (by similarity). Phosphorylation of AKT1/PKB thereby induces the phosphorylation of downstream effectors GSK3 and FOXO1/FKHR
CDKAL1	9606.ENSP00000274695	Threonylcarbamoyladenosine tRNA methylthiotransferase; Catalyzes the methylthiolation of N6- threonylcarbamoyladenosine (t(6)A), leading to the formation of 2-methylthio-N6-threonylcarbamoyladenosine (ms(2)t(6)A) at position 37 in tRNAs that read codons beginning with adenine; Belongs to the methylthiotransferase family. CDKAL1 subfamily
CLCC1	9606.ENSP00000349456	Chloride channel CLIC-like protein 1; Seems to act as a chloride ion channel; Tetraspan junctional complex superfamily
CLINT1	9606.ENSP00000429824	Clathrin interactor 1; Binds to membranes enriched in phosphatidylinositol 4,5-bisphosphate (PtdIns(4,5)P2). May have a role in transport via clathrin-coated vesicles from the trans-Golgi network to endosomes. Stimulates clathrin assembly
CTNND1	9606.ENSP00000382004	Catenin delta-1; Binds to and inhibits the transcriptional repressor ZBTB33, which may lead to the activation of target genes of the Wnt signaling pathway (By similarity). Associates with and regulates the cell adhesion properties of both C-, E- and N-cadherins, being critical for their surface stability. Implicated both in cell transformation by SRC and in ligand-induced receptor signaling through the EGF, PDGF, CSF-1, and ERBB2 receptors. Promotes GLIS2 C-terminal cleavage; Belongs to the beta-catenin family
DHRS13	9606.ENSP00000368173	Dehydrogenase/reductase SDR family member 13; Putative oxidoreductase
DLG1	9606.ENSP00000345731	Disks large homolog 1; Essential multidomain scaffolding protein required for normal development (By similarity). Recruits channels, receptors, and signaling molecules to discrete plasma membrane domains in polarized cells. May play a role in adherens junction assembly, signal transduction, cell proliferation, synaptogenesis, and lymphocyte activation. Regulates the excitability of cardiac myocytes by modulating the functional expression of Kv4 channels. Functional regulator of Kv1.5 channel; Belongs to the MAGUK family
DSC2	9606.ENSP00000280904	Desmocollin-2; Component of intercellular desmosome junctions. Involved in the interaction of plaque proteins and intermediate filaments mediating cell–cell adhesion. May contribute to epidermal cell positioning by mediating differential adhesiveness between cells that express different isoforms
DSG2	9606.ENSP00000261590	Desmoglein-2; Component of intercellular desmosome junctions. Involved in the interaction of plaque proteins and intermediate filaments mediating cell–cell adhesion
DST	9606.ENSP00000307959	Dystonin; Cytoskeletal linker protein. Acts as an integrator of intermediate filaments, actin and microtubule cytoskeleton networks. Required for anchoring either intermediate filaments to the actin cytoskeleton in neural and muscle cells or keratin-containing intermediate filaments to hemidesmosomes in epithelial cells. The proteins may self-aggregate to form filaments or a two-dimensional mesh. Regulates the organization and stability of the microtubule network of sensory neurons to allow axonal transport. Mediates docking of the dynein/dynactin motor complex to vesicle cargo
EFR3A	9606.ENSP00000254624	Protein EFR3 homolog A; Component of a complex required to localize phosphatidylinositol 4-kinase (PI4K) to the plasma membrane. The complex acts as a regulator of phosphatidylinositol 4-phosphate (PtdIns(4)P) synthesis (Probable). In the complex, EFR3A probably acts as the membrane-anchoring component. Also involved in responsiveness to G-protein-coupled receptors; it is, however, unclear whether this role is direct or indirect
EFR3B	9606.ENSP00000384081	Protein EFR3 homolog B; Component of a complex required to localize phosphatidylinositol 4-kinase (PI4K) to the plasma membrane. The complex acts as a regulator of phosphatidylinositol 4-phosphate (PtdIns(4)P) synthesis (Probable). In the complex, EFR3B probably acts as the membrane-anchoring component. Also involved in responsiveness to G-protein-coupled receptors; it is, however, unclear whether this role is direct or indirect; Armadillo-like helical domain containing
EPB41	9606.ENSP00000345259	Protein 4.1; Protein 4.1 is a major structural element of the erythrocyte membrane skeleton. It plays a key role in regulating membrane physical properties of mechanical stability and deformability by stabilizing spectrin–actin interaction. Recruits DLG1 to membranes. Required for dynein–dynactin complex and NUMA1 recruitment at the mitotic cell cortex during anaphase
EPB41L2	9606.ENSP00000338481	Band 4.1-like protein 2; Required for dynein–dynactin complex and NUMA1 recruitment at the mitotic cell cortex during anaphase; Erythrocyte membrane protein band 4.1
EPB41L3	9606.ENSP00000343158	Band 4.1-like protein 3; Tumor suppressor that inhibits cell proliferation and promotes apoptosis. Modulates the activity of protein arginine N-methyltransferases, including PRMT3 and PRMT5; Erythrocyte membrane protein band 4.1
EPS15	9606.ENSP00000360798	Epidermal growth factor receptor substrate 15; Involved in cell growth regulation. May be involved in the regulation of mitogenic signals and control of cell proliferation. Involved in the internalization of ligand-inducible receptors of the receptor tyrosine kinase (RTK) type, in particular EGFR. Plays a role in the assembly of clathrin-coated pits (CCPs). Acts as a clathrin adapter required for post-Golgi trafficking. Seems to be involved in CCPs maturation, including invagination or budding. Involved in endocytosis of integrin beta- 1 (ITGB1) and transferrin receptor (TFR)
ERBB2IP	9606.ENSP00000426632	Erbin; Acts as an adapter for the receptor ERBB2, in epithelia. By binding the unphosphorylated ‘Tyr-1248’ of receptor ERBB2, it may contribute to stabilizing this unphosphorylated state. Inhibits NOD2-dependent NF-kappa-B signaling and proinflammatory cytokine secretion; Belongs to the LAP (LRR and PDZ) protein family
ESYT1	9606.ENSP00000267113	Extended synaptotagmin-1; Binds glycerophospholipids in a barrel-like domain and may play a role in cellular lipid transport (By similarity). Binds calcium (via the C2 domains) and translocates to sites of contact between the endoplasmic reticulum and the cell membrane in response to increased cytosolic calcium levels. Helps tether the endoplasmic reticulum to the cell membrane and promotes the formation of appositions between the endoplasmic reticulum and the cell membrane; Belongs to the extended synaptotagmin family
FASN	9606.ENSP00000304592	Fatty acid synthase: Fatty acid synthetase catalyzes the formation of long-chain fatty acids from acetyl-CoA, malonyl-CoA, and NADPH. This multifunctional protein has 7 catalytic activities and an acyl carrier protein; a Seven-beta-strand methyltransferase motif containing
FCHO2	9606.ENSP00000393776	F-BAR domain only protein 2; Functions in an early step of clathrin-mediated endocytosis. Has both a membrane binding/bending activity and the ability to recruit proteins essential to the formation of functional clathrin-coated pits. Has a lipid-binding activity with a preference for membranes enriched in phosphatidylserine and phosphoinositides (Pi(4,5) biphosphate) like the plasma membrane. Its membrane-bending activity might be important for the subsequent action of clathrin and adaptors in the formation of clathrin-coated vesicles
FERMT2	9606.ENSP00000342858	Fermitin family homolog 2; Scaffolding protein that enhances integrin activation mediated by TLN1 and/or TLN2 but activates integrins only weakly by itself. Binds to membranes enriched in phosphoinositides. Enhances integrin-mediated cell adhesion onto the extracellular matrix and cell spreading; this requires both its ability to interact with integrins and with phospholipid membranes. Required for the assembly of focal adhesions. Participates in the connection between extracellular matrix adhesion sites and the actin cytoskeleton
FNDC3A	9606.ENSP00000441831	Fibronectin type-III domain-containing protein 3A; Mediates spermatid–Sertoli adhesion during spermatogenesis; Belongs to the FNDC3 family
GAB1	9606.ENSP00000262995	GRB2-associated-binding protein 1; Adapter protein that plays a role in intracellular signaling cascades triggered by activated receptor-type kinases. Plays a role in FGFR1 signaling. Probably involved in signaling by the epidermal growth factor receptor (EGFR) and the insulin receptor (INSR)
GAPVD1	9606.ENSP00000377664	GTPase-activating protein and VPS9 domain-containing protein 1; Acts both as a GTPase-activating protein (GAP) and a guanine nucleotide exchange factor (GEF), and participates in various processes such as endocytosis, insulin receptor internalization, or LC2A4/GLUT4 trafficking. Acts as a GEF for the Ras-related protein RAB31 by exchanging bound GDP for free GTP, leading to regulating LC2A4/GLUT4 trafficking. In the absence of insulin, it maintains RAB31 in an active state and promotes a futile cycle between LC2A4/GLUT4 storage vesicles and early endosomes
GJA1	9606.ENSP00000282561	Gap junction alpha-1 protein; Gap junction protein that acts as a regulator of bladder capacity. A gap junction consists of a cluster of closely packed pairs of transmembrane channels, the connexons, through which materials of low MW diffuse from one cell to a neighboring cell. May play a critical role in the physiology of hearing by participating in the recycling of potassium to the cochlear endolymph. Negative regulator of bladder functional capacity: acts by enhancing intercellular electrical and chemical transmission
GOLGB1	9606.ENSP00000377275	Golgin subfamily B member 1; May participate in forming intercisternal cross-bridges of the Golgi complex
GPRIN1	9606.ENSP00000305839	G protein-regulated inducer of neurite outgrowth 1; May be involved in neurite outgrowth
IFIT5	9606.ENSP00000360860	Interferon-induced protein with tetratricopeptide repeats 5; Interferon-induced RNA-binding protein involved in the human innate immune response. Has a broad and adaptable RNA structure recognition important for RNA recognition specificity in antiviral defense. Binds precursor and processed tRNAs as well as poly-U-tailed tRNA fragments. Specifically binds single-stranded RNA bearing a 5’-triphosphate group (PPP-RNA), thereby acting as a sensor of viral single-stranded RNAs.
IGF2R	9606.ENSP00000349437	Cation-independent mannose-6-phosphate receptor; Transport of phosphorylated lysosomal enzymes from the Golgi complex and the cell surface to lysosomes. Lysosomal enzymes bearing phosphomannosyl residues bind specifically to mannose-6- phosphate receptors in the Golgi apparatus, and the resulting receptor–ligand complex is transported to an acidic prelyosomal compartment where the low pH mediates the dissociation of the complex. This receptor also binds IGF2. Acts as a positive regulator of T-cell coactivation, by binding DPP4; CD molecules
IRS2	9606.ENSP00000365016	Insulin receptor substrate 2; May mediate the control of various cellular processes by insulin; Pleckstrin homology domain containing
JPH1	9606.ENSP00000344488	Junctophilin-1; Junctophilins contribute to the formation of junctional membrane complexes (JMCs), which link the plasma membrane with the endoplasmic or sarcoplasmic reticulum in excitable cells. Provides a structural foundation for functional cross-talk between the cell surface and intracellular calcium release channels. JPH1 contributes to the construction of the skeletal muscle triad by linking the t-tubule (transverse-tubule) and SR (sarcoplasmic reticulum) membranes
KIAA1524	9606.ENSP00000295746	Cellular inhibitor of pp2a; Protein CIP2A; Oncoprotein that inhibits PP2A and stabilizes MYC in human malignancies. Promotes anchorage-independent cell growth and tumor formation
KIDINS220	9606.ENSP00000256707	Ankyrin repeat-rich membrane spanning protein; Kinase D-interacting substrate of 220 kDa; Promotes a prolonged MAP-kinase signaling by neurotrophins through activation of a Rap1-dependent mechanism. Provides a docking site for the CRKL-C3G complex, resulting in Rap1-dependent sustained ERK activation. May play an important role in regulating postsynaptic signal transduction through the syntrophin-mediated localization of receptor tyrosine kinases such as EPHA4. In cooperation with SNTA1 can enhance EPHA4-induced JAK/STAT activation.
LBR	9606.ENSP00000339883	Delta14-sterol reductase (lamin-B receptor); Lamin-B receptor; Anchors the lamina and the heterochromatin to the inner nuclear membrane; Tudor domain containing
LLGL1	9606.ENSP00000321537	LLGL1, scribble cell polarity complex component; Lethal(2) giant larvae protein homolog 1; Cortical cytoskeleton protein found in a complex involved in maintaining cell polarity and epithelial integrity. Involved in the regulation of mitotic spindle orientation, proliferation, differentiation, and tissue organization of neuroepithelial cells. Involved in axonogenesis through RAB10 activation, thereby regulating vesicular membrane trafficking toward the axonal plasma membrane
LRBA	9606.ENSP00000349629	Lipopolysaccharide-responsive and beige-like anchor protein; May be involved in coupling signal transduction and vesicle trafficking to enable polarized secretion and/or membrane deposition of immune effector molecules; Armadillo-like helical domain containing
LRRC7	9606.ENSP00000035383	Leucine-rich repeat-containing protein 7; Required for normal synaptic spine architecture and function. Necessary for DISC1 and GRM5 localization to postsynaptic density complexes and for both N-methyl d-aspartate receptor-dependent and metabotropic glutamate receptor-dependent long-term depression; Belongs to the LAP (LRR and PDZ) protein family
LRRC8B	9606.ENSP00000332674	Volume-regulated anion channel subunit LRRC8B; Non-essential component of the volume-regulated anion channel (VRAC, also named VSOAC channel), an anion channel required to maintain a constant cell volume in response to extracellular or intracellular osmotic changes. The VRAC channel conducts iodide better than chloride and may also conduct organic osmolytes like taurine. Channel activity requires LRRC8A plus at least one other family member (LRRC8B, LRRC8C, LRRC8D or LRRC8E); channel characteristics depend on the precise subunit composition
LRRC8C	9606.ENSP00000359483	Volume-regulated anion channel subunit LRRC8C; Non-essential component of the volume-regulated anion channel (VRAC, also named VSOAC channel), an anion channel required to maintain a constant cell volume in response to extracellular or intracellular osmotic changes. The VRAC channel conducts iodide better than chloride and may also conduct organic osmolytes like taurine. Channel activity requires LRRC8A plus at least one other family member (LRRC8B, LRRC8C, LRRC8D or LRRC8E); channel characteristics depend on the precise subunit composition
LRRC8D	9606.ENSP00000338887	Volume-regulated anion channel subunit LRRC8D; Non-essential component of the volume-regulated anion channel (VRAC, also named VSOAC channel), an anion channel required to maintain a constant cell volume in response to extracellular or intracellular osmotic changes. The VRAC channel conducts iodide better than chloride and may also conduct organic osmolytes like taurine. Channel activity requires LRRC8A plus at least one other family member (LRRC8B, LRRC8C, LRRC8D or LRRC8E); channel characteristics depend on the precise subunit composition
LSG1	9606.ENSP00000265245	Large subunit GTPase 1 homolog; GTPase required for the XPO1/CRM1-mediated nuclear export of the 60 S ribosomal subunit. Probably acts by mediating the release of NMD3 from the 60S ribosomal subunit after export into the cytoplasm
LSR	9606.ENSP00000480821	Lipolysis-stimulated lipoprotein receptor; Probable role in the clearance of triglyceride-rich lipoprotein from blood. Binds chylomicrons, LDL, and VLDL in the presence of free fatty acids and allows their subsequent uptake in the cells (By similarity); Belongs to the immunoglobulin superfamily. LISCH7 family
MACF1	9606.ENSP00000354573	Microtubule-actin cross-linking factor 1, isoforms 1/2/3/5; Isoform 2: F-actin-binding protein which plays a role in cross-linking actin to other cytoskeletal proteins and also binds to microtubules. Plays an important role in ERBB2-dependent stabilization of microtubules at the cell cortex. Acts as a positive regulator of the Wnt receptor signaling pathway and is involved in the translocation of AXIN1 and its associated complex (composed of APC, CTNNB1, and GSK3B) from the cytoplasm to the cell membrane (By similarity).
MLLT4	9606.ENSP00000375960	Afadin; Belongs to an adhesion system, probably together with the E-cadherin-catenin system, which plays a role in the organization of homotypic, interneuronal, and heterotypic cell–cell adherens junctions (AJs). Nectin- and actin-filament-binding protein that connects nectin to the actin cytoskeleton
MYO6	9606.ENSP00000358994	Unconventional myosin-VI; Myosins are actin-based motor molecules with ATPase activity. Unconventional myosins serve in intracellular movements. Myosin 6 is a reverse-direction motor protein that moves towards the minus-end of actin filaments. Has a slow rate of actin-activated ADP release due to weak ATP binding. Functions in a variety of intracellular processes, such as vesicular membrane trafficking and cell migration. Required for the structural integrity of the Golgi apparatus via the p53-dependent pro-survival pathway.
NDC1	9606.ENSP00000360483	NDC1 transmembrane nucleoporin; Nucleoporin NDC1; Component of the nuclear pore complex (NPC), which plays a key role in de novo assembly and insertion of NPC in the nuclear envelope. Required for NPC and nuclear envelope assembly, possibly by forming a link between the nuclear envelope membrane and soluble nucleoporins, thereby anchoring the NPC in the membrane
NDRG1	9606.ENSP00000404854	N-myc downstream regulated 1; Protein NDRG1; Stress-responsive protein involved in hormone responses, cell growth, and differentiation. Acts as a tumor suppressor in many cell types. Necessary but not sufficient for p53/TP53-mediated caspase activation and apoptosis. Has a role in cell trafficking, notably of the Schwann cell, and is necessary for the maintenance and development of the peripheral nerve myelin sheath. Required for vesicular recycling of CDH1 and TF. May also function in lipid trafficking. Protects cells from spindle disruption damage.
NECTIN2	9606.ENSP00000252483	Nectin-2; Modulator of T-cell signaling. Can be either a costimulator of T-cell function or a coinhibitor depending on the receptor it binds to. Upon binding to CD226, stimulates T-cell proliferation and cytokine production, including that of IL2, IL5, IL10, IL13, and IFNG. Upon interaction with PVRIG, inhibits T-cell proliferation. These interactions are competitive. Probable cell adhesion protein; Belongs to the nectin family
NSDHL	9606.ENSP00000359297	Sterol-4-alpha-carboxylate 3-dehydrogenase, decarboxylating; Involved in the sequential removal of two C-4 methyl groups in post-squalene cholesterol biosynthesis; Short chain dehydrogenase/reductase superfamily
NUMB	9606.ENSP00000451300	Numb, endocytic adaptor protein; Protein numb homolog; Plays a role in the process of neurogenesis. Required throughout embryonic neurogenesis to maintain neural progenitor cells, also called radial glial cells (RGCs), by allowing their daughter cells to choose progenitor over neuronal cell fate. Not required for the proliferation of neural progenitor cells before the onset of neurogenesis. Also involved postnatally in the subventricular zone (SVZ) neurogenesis by regulating SVZ neuroblasts survival and ependymal wall integrity. May also mediate local repair of brain ventricular wall damage
NUMBL	9606.ENSP00000252891	Numb-like protein; Plays a role in the process of neurogenesis. Required throughout embryonic neurogenesis to maintain neural progenitor cells, also called radial glial cells (RGCs), by allowing their daughter cells to choose progenitor over neuronal cell fate. Not required for the proliferation of neural progenitor cells before the onset of embryonic neurogenesis. Also required postnatally in the subventricular zone (SVZ) neurogenesis by regulating SVZ neuroblasts survival and ependymal wall integrity. Negative regulator of NF-kappa-B-signaling pathway. The inhibition of NF-kappa-B a […]
OCLN	9606.ENSP00000347379	Occludin; May play a role in the formation and regulation of the tight junction (TJ) paracellular permeability barrier. It is able to induce adhesion when expressed in cells lacking tight junctions; Protein phosphatase 1 regulatory subunits
OSBPL8	9606.ENSP00000261183	Oxysterol-binding protein-related protein 8; Lipid transporter involved in lipid countertransport between the endoplasmic reticulum and the plasma membrane: specifically exchanges phosphatidylserine with phosphatidylinositol 4-phosphate (PI4P), delivering phosphatidylserine to the plasma membrane in exchange for PI4P, which is degraded by the SAC1/SACM1L phosphatase in the endoplasmic reticulum. Binds phosphatidylserine and PI4P in a mutually exclusive manner. Binds oxysterol, 25-hydroxycholesterol, and cholesterol; Belongs to the OSBP family
PAK4	9606.ENSP00000469413	Serine/threonine-protein kinase PAK 4: Serine/threonine protein kinase plays a role in a variety of different signaling pathways, including cytoskeleton regulation, cell migration, growth, proliferation, or cell survival. Activation by various effectors, including growth factor receptors or active CDC42 and RAC1, results in a conformational change and a subsequent autophosphorylation on several serine and/or threonine residues. Phosphorylates and inactivates the protein phosphatase SSH1, leading to increased inhibitory phosphorylation of the actin-binding/depolymerizing factor cofilin
PEAK1	9606.ENSP00000452796	Pseudopodium-enriched atypical kinase 1; Tyrosine kinase that may play a role in cell spreading and migration on fibronectin. May directly or indirectly affect phosphorylation levels of cytoskeleton-associated proteins MAPK1/ERK and PXN
PHACTR4	9606.ENSP00000362942	Phosphatase and actin regulator 4; Regulator of protein phosphatase 1 (PP1) required for neural tube and optic fissure closure, and enteric neural crest cell (ENCCs) migration during development. Acts as an activator of PP1 by interacting with PPP1CA and preventing phosphorylation of PPP1CA at ‘Thr-320’. During neural tube closure, localizes to the ventral neural tube and activates PP1, leading to down-regulate cell proliferation within cranial neural tissue and the neural retina. Also acts as a regulator of migration of enteric neural crest cells (ENCCs) by activating PP1
PKN2	9606.ENSP00000359552	Serine/threonine-protein kinase N2; PKC-related serine/threonine-protein kinase and Rho/Rac effector protein that participates in specific signal transduction responses in the cell. Plays a role in the regulation of cell cycle progression, actin cytoskeleton assembly, cell migration, cell adhesion, tumor cell invasion, and transcription activation signaling processes. Phosphorylates CTTN in hyaluronan-induced astrocytes and hence decreases CTTN's ability to associate with filamentous actin. Phosphorylates HDAC5, therefore, lead to impaired HDAC5 import.
PLEKHA5	9606.ENSP00000404296	Pleckstrin homology domain-containing family a member 5; Pleckstrin homology domain-containing A5
PPFIBP1	9606.ENSP00000314724	Ppfia binding protein 1; Liprin-beta-1; May regulate the disassembly of focal adhesions. Did not bind receptor-like tyrosine phosphatases type 2A; Sterile alpha motif domain containing
PREB	9606.ENSP00000260643	Prolactin regulatory element-binding protein; Guanine nucleotide exchange factor that specifically activates the small GTPase SAR1B. Mediates the recruitment of SAR1B and other COPII coat components to endoplasmic reticulum membranes and is therefore required for the formation of COPII transport vesicles from the ER; WD repeat domain-containing
PSD3	9606.ENSP00000324127	PH and SEC7 domain-containing protein 3; Guanine nucleotide exchange factor for ARF6; Pleckstrin homology domain containing
PTPN1	9606.ENSP00000360683	Tyrosine-protein phosphatase non-receptor type 1; Tyrosine-protein phosphatase which acts as a regulator of endoplasmic reticulum unfolded protein response. Mediates dephosphorylation of EIF2AK3/PERK; inactivating the protein kinase activity of EIF2AK3/PERK. May play an important role in CKII- and p60c-src-induced signal transduction cascades. It may regulate the EFNA5–EPHA3 signaling pathway, which modulates cell reorganization and cell-cell repulsion. May also regulate the hepatocyte growth factor receptor signaling pathway through the dephosphorylation of MET
PTPN13	9606.ENSP00000394794	Tyrosine-protein phosphatase non-receptor type 13; Tyrosine phosphatase which regulates negatively FAS-induced apoptosis and NGFR-mediated pro-apoptotic signaling. May regulate phosphoinositide 3-kinase (PI3K) signaling through dephosphorylation of PIK3R2; FERM domain containing
RAB23	9606.ENSP00000417610	RAB23, member RAS oncogene family; Ras-related protein Rab-23; The small GTPases Rab are key regulators of intracellular membrane trafficking, from the formation of transport vesicles to their fusion with membranes. Rabs cycle between an inactive GDP-bound form and an active GTP-bound form that is able to recruit to membranes different sets of downstream effectors directly responsible for vesicle formation, movement, tethering, and fusion. Together with SUFU, it prevents nuclear import of GLI1, and thereby inhibits GLI1 transcription factor activity.
RAI14	9606.ENSP00000427123	Retinoic acid-induced 14; Ankycorbin; Plays a role in actin regulation at the ectoplasmic specialization, a type of cell junction specific to the testis. Important for the establishment of sperm polarity and normal spermatid adhesion. May also promote the integrity of Sertoli cell tight junctions at the blood-testis barrier; Ankyrin repeat domain-containing
RAPGEF6	9606.ENSP00000296859	Rap guanine nucleotide exchange factor 6; Guanine nucleotide exchange factor (GEF) for Rap1A, Rap2A, and M-Ras GTPases. Does not interact with cAMP; PDZ domain-containing
RASAL2	9606.ENSP00000356621	Ras GTPase-activating protein nGAP; Inhibitory regulator of the Ras-cyclic AMP pathway; C2 and RasGAP domain containing
RICTOR	9606.ENSP00000296782	Rapamycin-insensitive companion of mTOR; Subunit of mTORC2, which regulates cell growth and survival in response to hormonal signals. mTORC2 is activated by growth factors, but, in contrast to mTORC1, seems to be nutrient-insensitive. mTORC2 seems to function upstream of Rho GTPases to regulate the actin cytoskeleton, probably by activating one or more Rho-type guanine nucleotide exchange factors. mTORC2 promotes the serum-induced formation of stress fibers or F-actin. mTORC2 plays a critical role in AKT1 ‘
ROR2	9606.ENSP00000364860	Tyrosine-protein kinase transmembrane receptor ROR2; Tyrosine-protein kinase receptor which may be involved in the early formation of the chondrocytes. It seems to be required for cartilage and growth plate development (By similarity). Phosphorylates YWHAB, leading to induction of osteogenesis and bone formation. In contrast, has also been shown to have very little tyrosine kinase activity in vitro. May act as a receptor for wnt ligand WNT5A, which may result in the inhibition of WNT3A-mediated signaling; I-set domain containing
RUVBL1	9606.ENSP00000318297	RuvB-like 1; May be able to bind plasminogen at the cell surface and enhance plasminogen activation; AAA ATPases
SCRIB	9606.ENSP00000349486	Protein scribble homolog: Scaffold protein involved in different aspects of polarized cell differentiation regulating epithelial and neuronal morphogenesis. Most probably functions in the establishment of apico-basal cell polarity. May function in cell proliferation regulating progression from G1 to S phase and as a positive regulator of apoptosis for instance during acinar morphogenesis of the mammary epithelium. May also function in cell migration and adhesion and hence regulate cell invasion through MAPK signaling. May play a role in exocytosis and in the targeting synaptic vesicle
SEC24B	9606.ENSP00000428564	Protein transport protein Sec24B; Component of the coat protein complex II (COPII) which promotes the formation of transport vesicles from the endoplasmic reticulum (ER). The coat has two main functions, the physical deformation of the endoplasmic reticulum membrane into vesicles and the selection of cargo molecules for their transport to the Golgi complex. Plays a central role in cargo selection within the COPII complex and, together with SEC24A, may have a different specificity compared to SEC24C and SEC24D. May package preferentially cargos with cytoplasmic DxE or LxxLE motifs
SEPT9	9606.ENSP00000391249	Septin-9; Filament-forming cytoskeletal GTPase (By similarity). May play a role in cytokinesis (Potential). May play a role in the internalization of 2 intracellular microbial pathogens. Belongs to the TRAFAC class TrmE-Era-EngA-EngB-Septin- like GTPase superfamily. Septin GTPase family
SH3D19	9606.ENSP00000302913	SH3 domain-containing protein 19; May play a role in regulating A disintegrin and metalloproteases (ADAMs) in the signaling of EGFR-ligand shedding. May be involved in suppression of Ras-induced cellular transformation and Ras-mediated activation of ELK1. Plays a role in the regulation of cell morphology and cytoskeletal organization
SLC26A6	9606.ENSP00000378920	Solute carrier family 26 member 6; Apical membrane anion-exchanger with a wide epithelial distribution that plays a role as a component of the pH buffering system for maintaining acid-base homeostasis. Acts as a versatile DIDS-sensitive inorganic and organic anion transporter that mediates the uptake of monovalent anions like chloride, bicarbonate, formate, and hydroxyl ion and divalent anions like sulfate and oxalate. Function in multiple exchange modes involving pairs of these anions
SLC38A1	9606.ENSP00000449756	Sodium-coupled neutral amino acid transporter 1; Functions as a sodium-dependent amino acid transporter. Mediates the saturable, pH-sensitive, and electrogenic cotransport of glutamine and sodium ions with a stoichiometry of 1:1. May also transport small zwitterionic and aliphatic amino acids with a lower affinity. May supply glutamatergic and GABAergic neurons with glutamine which is required for the synthesis of the neurotransmitters glutamate and GABA; Solute carriers
SLC39A10	9606.ENSP00000386766	Solute carrier family 39 (zinc transporter), member 10; Zinc transporter ZIP10; May act as a zinc-influx transporter; Belongs to the ZIP transporter (TC 2.A.5) family
SLC3A2	9606.ENSP00000367123	4F2 cell-surface antigen heavy chain; Required for the function of light chain amino-acid transporters. Involved in sodium-independent, high-affinity transport of large neutral amino acids such as phenylalanine, tyrosine, leucine, arginine, and tryptophan. Involved in guiding and targeting LAT1 and LAT2 to the plasma membrane. When associated with SLC7A6 or SLC7A7 acts as an arginine/glutamine exchanger, following an antiport mechanism for amino acid transport, influencing arginine release in exchange for extracellular amino acids.
SLC6A15	9606.ENSP00000266682	Sodium-dependent neutral amino acid transporter B(0)AT2; Functions as a sodium-dependent neutral amino acid transporter. Exhibits preference for the branched-chain amino acids, particularly leucine, valine, and isoleucine, and methionine. Mediates the saturable, pH-sensitive, and electrogenic cotransport of proline and sodium ions with a stoichiometry of 1:1. May have a role as a transporter for neurotransmitter precursors into neurons. In contrast to other members of the neurotransmitter transporter family, does not appear to be chloride-dependent; Solute carriers
SNAP23	9606.ENSP00000249647	Synaptosomal-associated protein 23; Essential component of the high-affinity receptor for the general membrane fusion machinery and an important regulator of transport vesicle docking and fusion; Belongs to the SNAP-25 family
SNX1	9606.ENSP00000261889	Sorting nexin-1; Involved in several stages of intracellular trafficking. Interacts with membranes containing phosphatidylinositol 3- phosphate (PtdIns(3P)) or phosphatidylinositol 3,5-bisphosphate (PtdIns(3,5)P2). Acts in part as a component of the retromer membrane-deforming SNX-BAR subcomplex. The SNX-BAR retromer mediates retrograde transport of cargo proteins from endosomes to the trans-Golgi network (TGN) and is involved in endosome-to-plasma membrane transport for cargo protein recycling. The SNX-BAR subcomplex functions to deform the donor membrane into a tubular profile
SPTAN1	9606.ENSP00000361824	Spectrin alpha chain, non-erythrocytic 1; Fodrin, which seems to be involved in secretion, interacts with calmodulin in a calcium-dependent manner and is thus a candidate for the calcium-dependent movement of the cytoskeleton at the membrane; EF-hand domain containing
SRPRA	9606.ENSP00000328023	Signal recognition particle receptor subunit alpha; Component of the SRP (signal recognition particle) receptor. Ensures, in conjunction with the signal recognition particle, the correct targeting of the nascent secretory proteins to the endoplasmic reticulum membrane system
STAMBP	9606.ENSP00000377633	STAM-binding protein; Zinc metalloprotease that specifically cleaves ‘Lys-63’- linked polyubiquitin chains. Does not cleave ‘Lys-48’-linked polyubiquitin chains (By similarity). Plays a role in signal transduction for cell growth and MYC induction mediated by IL-2 and GM-CSF. Potentiates BMP (bone morphogenetic protein) signaling by antagonizing the inhibitory action of SMAD6 and SMAD7. Has a key role in the regulation of cell surface receptor-mediated endocytosis and ubiquitin-dependent sorting of receptors to lysosomes. Endosomal localization of STAMBP is required for efficient EGFR degradation
STEAP3	9606.ENSP00000376822	Metalloreductase STEAP3; Endosomal ferrireductase required for efficient transferrin-dependent iron uptake in erythroid cells. Participates in erythroid iron homeostasis by reducing Fe(3+) to Fe(2+). Can also reduce Cu(2+) to Cu(1+), suggesting that it participates in copper homeostasis. Uses NADP(+) as an acceptor. May play a role downstream of p53/TP53 to interface apoptosis and cell cycle progression. Indirectly involved in exosome secretion by facilitating the secretion of proteins such as TCTP; STEAP family
STIM1	9606.ENSP00000478059	Stromal interaction molecule 1; Plays a role in mediating store-operated Ca(2+) entry (SOCE), a Ca(2+) influx following the depletion of intracellular Ca(2+) stores. Acts as Ca(2+) sensor in the endoplasmic reticulum via its EF-hand domain. Upon Ca(2+) depletion, it translocates from the endoplasmic reticulum to the plasma membrane, where it activates the Ca(2+) release-activated Ca(2+) (CRAC) channel subunit ORAI1. Involved in enamel formation. Activated following interaction with STIMATE, leading to promote STIM1 conformational switch; Sterile alpha motif domain containing
SUGT1	9606.ENSP00000367208	SGT1 homolog, MIS12 kinetochore complex assembly cochaperone; Protein SGT1 homolog; May play a role in ubiquitination and subsequent proteasomal degradation of target proteins
TACC1	9606.ENSP00000321703	Transforming acidic coiled-coil-containing protein 1; Likely involved in the processes that promote cell division prior to the formation of differentiated tissues
TMEM57	9606.ENSP00000363463	Macoilin 1; Plays a role in the regulation of neuronal activity
TMPO	9606.ENSP00000266732	Lamina-associated polypeptide 2, isoform alpha; May be involved in the structural organization of the nucleus and in the post-mitotic nuclear assembly. Plays an important role, together with LMNA, in the nuclear anchorage of RB1; Belongs to the LEM family
TOR1AIP1	9606.ENSP00000435365	Torsin-1A-interacting protein 1; Required for nuclear membrane integrity. Induces TOR1A and TOR1B ATPase activity and is required for their location on the nuclear membrane. Binds to A- and B-type lamins. Possible role in membrane attachment and assembly of the nuclear lamina
TTK	9606.ENSP00000358813	Serine/threonine-protein kinase ttk/mps1; Dual specificity protein kinase TTK; Phosphorylates proteins on serine, threonine, and tyrosine. Probably associated with cell proliferation. Essential for chromosome alignment by enhancing AURKB activity (via direct CDCA8 phosphorylation) at the centromere, and for the mitotic checkpoint
UBE2J1	9606.ENSP00000451261	Ubiquitin-conjugating enzyme E2 J1; Catalyzes the covalent attachment of ubiquitin to other proteins. Functions in the selective degradation of misfolded membrane proteins from the endoplasmic reticulum (ERAD); Belongs to the ubiquitin-conjugating enzyme family
UBIAD1	9606.ENSP00000366006	UbiA prenyltransferase domain-containing protein 1; Prenyltransferase that mediates the formation of menaquinone-4 (MK-4) and coenzyme Q10. MK-4 is a vitamin K2 isoform present at high concentrations in the brain, kidney, and pancreas, and is required for endothelial cell development. Mediates the conversion of phylloquinone (PK) into MK-4, probably by cleaving the side chain of phylloquinone (PK) to release 2- methyl-1,4-naphthoquinone (menadione; K3) and then prenylating it with geranylgeranyl pyrophosphate (GGPP) to form MK-4.
USP6NL	9606.ENSP00000277575	USP6 N-terminal-like protein; Acts as a GTPase-activating protein for RAB5A and RAB43. Involved in receptor trafficking. In complex with EPS8, it inhibits the internalization of EGFR. Involved in retrograde transport from the endocytic pathway to the Golgi apparatus. Involved in the transport of Shiga toxin from early and recycling endosomes to the trans-Golgi network. Required for the structural integrity of the Golgi complex
UTRN	9606.ENSP00000356515	Utrophin; May play a role in anchoring the cytoskeleton to the plasma membrane; Zinc fingers ZZ-type
VANGL1	9606.ENSP00000347672	VANGL planar cell polarity protein 1
VAPB	9606.ENSP00000417175	Vesicle-associated membrane protein-associated protein B/C; Participates in the endoplasmic reticulum unfolded protein response (UPR) by inducing ERN1/IRE1 activity. Involved in cellular calcium homeostasis regulation
VRK2	9606.ENSP00000408002	Serine/threonine-protein kinase VRK2; Serine/threonine kinase that regulates several signal transduction pathways. Isoform 1 modulates the stress response to hypoxia and cytokines, such as interleukin-1 beta (IL1B), and this is dependent on its interaction with MAPK8IP1, which assembles mitogen-activated protein kinase (MAPK) complexes. Inhibition of signal transmission mediated by the assembly of MAPK8IP1–MAPK complexes reduces JNK phosphorylation and JUN-dependent transcription. Phosphorylates ‘Thr-18’ of p53/TP53, histone H3, and may also phosphorylate MAPK8IP1
WDR45B	9606.ENSP00000376139	Wd repeat domain phosphoinositide-interacting protein 3; Component of the autophagy machinery that controls the major intracellular degradation process by which cytoplasmic materials are packaged into autophagosomes and delivered to lysosomes for degradation. Binds phosphatidylinositol 3-phosphate (PtdIns3P) forming on membranes of the endoplasmic reticulum upon activation of the upstream ULK1 and PI3 kinases and is recruited at phagophore assembly sites where it regulates the elongation of nascent phagophores downstream of WIPI2
WIPI2	9606.ENSP00000288828	WD repeat domain phosphoinositide-interacting protein 2; Early component of the autophagy machinery being involved in the formation of preautophagosomal structures and their maturation into mature phagosomes in response to phosphatidylinositol 3-phosphate (PtdIns3P). May bind PtdIns3P
WWOX	9606.ENSP00000457230	WW domain-containing oxidoreductase; Putative oxidoreductase. Acts as a tumor suppressor and plays a role in apoptosis. Required for normal bone development (By similarity). May function synergistically with p53/TP53 to control genotoxic stress-induced cell death. Plays a role in TGFB1 signaling and TGFB1-mediated cell death. May also play a role in tumor necrosis factor (TNF)-mediated cell death. Inhibits Wnt signaling, probably by sequestering DVL2 in the cytoplasm; Short chain dehydrogenase/reductase superfamily
YKT6	9606.ENSP00000223369	Synaptobrevin homolog YKT6; Vesicular soluble NSF attachment protein receptor (v-SNARE) mediating vesicle docking and fusion to a specific acceptor cellular compartment. Functions in endoplasmic reticulum to Golgi transport as part of a SNARE complex composed of GOSR1, GOSR2, and STX5. Functions in early/recycling endosome to TGN transport as part of a SNARE complex composed of BET1L, GOSR1, and STX5. Has a S-palmitoyl transferase activity; SNAREs
ZC3HAV1	9606.ENSP00000242351	Zinc finger CCCH-type antiviral protein 1; Antiviral protein that inhibits the replication of viruses by recruiting the cellular RNA degradation machinery to degrade the viral mRNAs. Binds to a ZAP-responsive element (ZRE) present in the target viral mRNA, recruits cellular poly(A)- specific ribonuclease PARN to remove the poly(A) tail, and the 3’- 5’ exoribonuclease complex exosome to degrade the RNA body from the 3’-end. It also recruits the decapping complex DCP1–DCP2 through RNA helicase p72 (DDX17) to remove the cap structure of the viral mRNA to initiate its degradation
ZDHHC5	9606.ENSP00000287169	Palmitoyltransferase ZDHHC5; Palmitoyl acyltransferase for the G-protein-coupled receptor SSTR5. Also palmitoylates FLOT2 (By similarity); Zinc fingers DHHC-type
ZFYVE16	9606.ENSP00000337159	Mad, mothers against decapentaplegic interacting protein; Zinc finger FYVE domain-containing protein 16; May be involved in regulating membrane trafficking in the endosomal pathway. Overexpression induces endosome aggregation. Required to target TOM1 to endosomes; Protein phosphatase 1 regulatory subunits
ZFYVE9	9606.ENSP00000287727	Mad, mothers against decapentaplegic interacting protein; Zinc finger FYVE domain-containing protein 9; Early endosomal protein that functions to recruit SMAD2/SMAD3 to intracellular membranes and to the TGF-beta receptor. Plays a significant role in TGF-mediated signaling by regulating the subcellular location of SMAD2 and SMAD3 and modulating the transcriptional activity of the SMAD3/SMAD4 complex. Possibly associated with TGF-beta receptor internalization; Zinc fingers FYVE-type

https://string-db.org/.

New Table 3

**Table 3:** List and annotations of hit proteins from BioID analyses.

Protein	stringID	Annotation
ABCC5	9606.ENSP00000333926	Multidrug resistance-associated protein 5; Acts as a multispecific organic anion pump that can transport nucleotide analogs; Belongs to the ABC transporter superfamily. ABCC family. Conjugate transporter (TC 3.A.1.208) subfamily
ABR	9606.ENSP00000303909	Active breakpoint cluster region-related protein; GTPase-activating protein for RAC and CDC42. Promotes the exchange of RAC or CDC42-bound GDP by GTP, thereby activating them; C2 domain containing
ACBD3	9606.ENSP00000355777	Acyl-coa-binding domain containing 3; Golgi resident protein GCP60; Involved in the maintenance of Golgi structure by interacting with giantin, affecting protein transport between the endoplasmic reticulum and Golgi. Involved in hormone-induced steroid biosynthesis in testicular Leydig cells (By similarity). Recruits PI4KB to the Golgi apparatus membrane; enhances the enzyme activity of PI4KB activity via its membrane recruitment, thereby increasing the local concentration of the substrate in the vicinity of the kinase; A-kinase anchoring proteins
ADD2	9606.ENSP00000264436	Beta-adducin; Membrane-cytoskeleton-associated protein that promotes the assembly of the spectrin-actin network. Binds to the erythrocyte membrane receptor SLC2A1/GLUT1 and may, therefore, provide a link between the spectrin cytoskeleton to the plasma membrane. Binds to calmodulin. Calmodulin binds preferentially to the beta subunit; Belongs to the aldolase class II family.
ADD3	9606.ENSP00000348381	Gamma-adducin; Membrane-cytoskeleton-associated protein that promotes the assembly of the spectrin–actin network. Plays a role in actin filament capping. Binds to calmodulin; Belongs to the aldolase class II family. Adducin subfamily
AHNAK	9606.ENSP00000367263	Neuroblast differentiation-associated protein AHNAK; May be required for neuronal cell differentiation; PDZ domain-containing
AKAP12	9606.ENSP00000384537	A-kinase anchor protein 12; Anchoring protein that mediates the subcellular compartmentation of protein kinase A (PKA) and protein kinase C (PKC); A-kinase anchoring proteins
ALDH3A2	9606.ENSP00000345774	Aldehyde dehydrogenase 3 family member a2; Fatty aldehyde dehydrogenase; Catalyzes the oxidation of long-chain aliphatic aldehydes to fatty acids. Active on a variety of saturated and unsaturated aliphatic aldehydes between 6 and 24 carbons in length. Responsible for conversion of the sphingosine 1-phosphate (S1P) degradation product hexadecenal to hexadecenoic acid
ANKS1A	9606.ENSP00000353518	Ankyrin repeat and SAM domain-containing protein 1A; Regulator of different signaling pathways. Regulates EPHA8 receptor tyrosine kinase signaling to control cell migration and neurite retraction (By similarity); Ankyrin repeat domain-containing
ARHGAP21	9606.ENSP00000379709	Rho GTPase-activating protein 21; Functions as a GTPase-activating protein (GAP) for RHOA and CDC42. Downstream partner of ARF1, which may control Golgi apparatus structure and function. Also required for CTNNA1 recruitment to adherens junctions; PDZ domain-containing
ARHGAP39	9606.ENSP00000366522	Rho gtpase-activating protein 39; Rho GTPase-activating protein 39
ATP2B1	9606.ENSP00000392043	Plasma membrane calcium-transporting ATPase 1; This magnesium-dependent enzyme catalyzes the hydrolysis of ATP coupled with the transport of calcium out of the cell; ATPases Ca2+ transporting
CCDC88A	9606.ENSP00000338728	Coiled-coil domain-containing 88a; Girdin; Plays a role as a key modulator of the AKT-mTOR signaling pathway controlling the tempo of the process of newborn neuron integration during adult neurogenesis, including correct neuron positioning, dendritic development and synapse formation (By similarity). Enhances phosphoinositide 3-kinase (PI3K)-dependent phosphorylation and kinase activity of AKT1/PKB, but does not possess kinase activity itself (By similarity). Phosphorylation of AKT1/PKB thereby induces the phosphorylation of downstream effectors GSK3 and FOXO1/FKHR
CDKAL1	9606.ENSP00000274695	Threonylcarbamoyladenosine tRNA methylthiotransferase; Catalyzes the methylthiolation of N6-threonylcarbamoyladenosine (t(6)A), leading to the formation of 2-methylthio-N6-threonylcarbamoyladenosine (ms(2)t(6)A) at position 37 in tRNAs that read codons beginning with adenine; Belongs to the methylthiotransferase family. CDKAL1 subfamily
CLCC1	9606.ENSP00000349456	Chloride channel CLIC-like protein 1; Seems to act as a chloride ion channel; Tetraspan junctional complex superfamily
CLINT1	9606.ENSP00000429824	Clathrin interactor 1; Binds to membranes enriched in phosphatidylinositol 4,5- bisphosphate (PtdIns(4,5)P2). May have a role in transport via clathrin-coated vesicles from the trans-Golgi network to endosomes. Stimulates clathrin assembly
CTNND1	9606.ENSP00000382004	Catenin delta-1; Binds to and inhibits the transcriptional repressor ZBTB33, which may lead to the activation of target genes of the Wnt signaling pathway (By similarity). Associates with and regulates the cell adhesion properties of both C-, E- and N-cadherins, being critical for their surface stability. Implicated both in cell transformation by SRC and in ligand-induced receptor signaling through the EGF, PDGF, CSF-1, and ERBB2 receptors. Promotes GLIS2 C-terminal cleavage; Belongs to the beta-catenin family
DHRS13	9606.ENSP00000368173	Dehydrogenase/reductase SDR family member 13; Putative oxidoreductase
DLG1	9606.ENSP00000345731	Disks large homolog 1; Essential multidomain scaffolding protein required for normal development (By similarity). Recruits channels, receptors, and signaling molecules to discrete plasma membrane domains in polarized cells. May play a role in adherens junction assembly, signal transduction, cell proliferation, synaptogenesis, and lymphocyte activation. Regulates the excitability of cardiac myocytes by modulating the functional expression of Kv4 channels. Functional regulator of Kv1.5 channel; Belongs to the MAGUK family
DSC2	9606.ENSP00000280904	Desmocollin-2; Component of intercellular desmosome junctions. Involved in the interaction of plaque proteins and intermediate filaments mediating cell-cell adhesion. May contribute to epidermal cell positioning by mediating differential adhesiveness between cells that express different isoforms
DSG2	9606.ENSP00000261590	Desmoglein-2; Component of intercellular desmosome junctions. Involved in the interaction of plaque proteins and intermediate filaments mediating cell–cell adhesion
DST	9606.ENSP00000307959	Dystonin; Cytoskeletal linker protein. Acts as an integrator of intermediate filaments, actin and microtubule cytoskeleton networks. Required for anchoring either intermediate filaments to the actin cytoskeleton in neural and muscle cells or keratin-containing intermediate filaments to hemidesmosomes in epithelial cells. The proteins may self-aggregate to form filaments or a two-dimensional mesh. Regulates the organization and stability of the microtubule network of sensory neurons to allow axonal transport. Mediates docking of the dynein/dynactin motor complex to vesicle cargo
EFR3A	9606.ENSP00000254624	Protein EFR3 homolog A; Component of a complex required to localize phosphatidylinositol 4-kinase (PI4K) to the plasma membrane. The complex acts as a regulator of phosphatidylinositol 4-phosphate (PtdIns(4)P) synthesis (Probable). In the complex, EFR3A probably acts as the membrane-anchoring component. Also involved in responsiveness to G-protein-coupled receptors; it is, however unclear whether this role is direct or indirect
EFR3B	9606.ENSP00000384081	Protein EFR3 homolog B; Component of a complex required to localize phosphatidylinositol 4-kinase (PI4K) to the plasma membrane. The complex acts as a regulator of phosphatidylinositol 4-phosphate (PtdIns(4)P) synthesis (Probable). In the complex, EFR3B probably acts as the membrane-anchoring component. Also involved in responsiveness to G-protein-coupled receptors; it is, however, unclear whether this role is direct or indirect; Armadillo-like helical domain containing
EPB41	9606.ENSP00000345259	Protein 4.1; Protein 4.1 is a major structural element of the erythrocyte membrane skeleton. It plays a key role in regulating membrane physical properties of mechanical stability and deformability by stabilizing spectrin–actin interaction. Recruits DLG1 to membranes. Required for dynein–dynactin complex and NUMA1 recruitment at the mitotic cell cortex during anaphase
EPB41L2	9606.ENSP00000338481	Band 4.1-like protein 2; Required for dynein–dynactin complex and NUMA1 recruitment at the mitotic cell cortex during anaphase; Erythrocyte membrane protein band 4.1
EPB41L3	9606.ENSP00000343158	Band 4.1-like protein 3; Tumor suppressor that inhibits cell proliferation and promotes apoptosis. Modulates the activity of protein arginine N-methyltransferases, including PRMT3 and PRMT5; Erythrocyte membrane protein band 4.1
EPS15	9606.ENSP00000360798	Epidermal growth factor receptor substrate 15; Involved in cell growth regulation. May be involved in the regulation of mitogenic signals and control of cell proliferation. Involved in the internalization of ligand-inducible receptors of the receptor tyrosine kinase (RTK) type, in particular EGFR. Plays a role in the assembly of clathrin-coated pits (CCPs). Acts as a clathrin adapter required for post-Golgi trafficking. Seems to be involved in CCPs maturation, including invagination or budding. Involved in endocytosis of integrin beta- 1 (ITGB1) and transferrin receptor (TFR)
ERBB2IP	9606.ENSP00000426632	Erbin; Acts as an adapter for the receptor ERBB2, in epithelia. By binding the unphosphorylated ‘Tyr-1248’ of receptor ERBB2, it may contribute to stabilize this unphosphorylated state. Inhibits NOD2-dependent NF-kappa-B signaling and proinflammatory cytokine secretion; Belongs to the LAP (LRR and PDZ) protein family
ESYT1	9606.ENSP00000267113	Extended synaptotagmin-1; Binds glycerophospholipids in a barrel-like domain and may play a role in cellular lipid transport (By similarity). Binds calcium (via the C2 domains) and translocates to sites of contact between the endoplasmic reticulum and the cell membrane in response to increased cytosolic calcium levels. Helps tether the endoplasmic reticulum to the cell membrane and promotes the formation of appositions between the endoplasmic reticulum and the cell membrane; Belongs to the extended synaptotagmin family
FASN	9606.ENSP00000304592	Fatty acid synthase; Fatty acid synthetase catalyzes the formation of long-chain fatty acids from acetyl-CoA, malonyl-CoA, and NADPH. This multifunctional protein has 7 catalytic activities and an acyl carrier protein; Seven-beta-strand methyltransferase motif containing
FCHO2	9606.ENSP00000393776	F-BAR domain only protein 2; Functions in an early step of clathrin-mediated endocytosis. Has both a membrane binding/bending activity and the ability to recruit proteins essential to the formation of functional clathrin-coated pits. Has a lipid-binding activity with a preference for membranes enriched in phosphatidylserine and phosphoinositides (Pi(4,5) biphosphate) like the plasma membrane. Its membrane-bending activity might be important for the subsequent action of clathrin and adaptors in the formation of clathrin-coated vesicles
FERMT2	9606.ENSP00000342858	Fermitin family homolog 2; Scaffolding protein that enhances integrin activation mediated by TLN1 and/or TLN2 but activates integrins only weakly by itself. Binds to membranes enriched in phosphoinositides. Enhances integrin-mediated cell adhesion onto the extracellular matrix and cell spreading; this requires both its ability to interact with integrins and with phospholipid membranes. Required for the assembly of focal adhesions. Participates in the connection between extracellular matrix adhesion sites and the actin cytoskeleton
FNDC3A	9606.ENSP00000441831	Fibronectin type-III domain-containing protein 3A; Mediates spermatid–sertoli adhesion during spermatogenesis; Belongs to the FNDC3 family
GAB1	9606.ENSP00000262995	GRB2-associated-binding protein 1; Adapter protein that plays a role in intracellular signaling cascades triggered by activated receptor-type kinases. Plays a role in FGFR1 signaling. Probably involved in signaling by the epidermal growth factor receptor (EGFR) and the insulin receptor (INSR)
GAPVD1	9606.ENSP00000377664	GTPase-activating protein and VPS9 domain-containing protein 1; Acts both as a GTPase-activating protein (GAP) and a guanine nucleotide exchange factor (GEF), and participates in various processes such as endocytosis, insulin receptor internalization, or LC2A4/GLUT4 trafficking. Acts as a GEF for the Ras-related protein RAB31 by exchanging bound GDP for free GTP, leading to regulate LC2A4/GLUT4 trafficking. In the absence of insulin, it maintains RAB31 in an active state and promotes a futile cycle between LC2A4/GLUT4 storage vesicles and early endosomes
GJA1	9606.ENSP00000282561	Gap junction alpha-1 protein; Gap junction protein that acts as a regulator of bladder capacity. A gap junction consists of a cluster of closely packed pairs of transmembrane channels, the connexons, through which materials of low MW diffuse from one cell to a neighboring cell. May play a critical role in the physiology of hearing by participating in the recycling of potassium to the cochlear endolymph. Negative regulator of bladder functional capacity: acts by enhancing intercellular electrical and chemical transmission
GOLGB1	9606.ENSP00000377275	Golgin subfamily B member 1; May participate in forming intercisternal cross-bridges of the Golgi complex
GPRIN1	9606.ENSP00000305839	G protein-regulated inducer of neurite outgrowth 1; May be involved in neurite outgrowth
IFIT5	9606.ENSP00000360860	Interferon-induced protein with tetratricopeptide repeats 5; Interferon-induced RNA-binding protein involved in the human innate immune response. Has a broad and adaptable RNA structure recognition important for RNA recognition specificity in antiviral defense. Binds precursor and processed tRNAs as well as poly-U-tailed tRNA fragments. Specifically binds single-stranded RNA bearing a 5’-triphosphate group (PPP-RNA), thereby acting as a sensor of viral single-stranded RNAs.
IGF2R	9606.ENSP00000349437	Cation-independent mannose-6-phosphate receptor; Transport of phosphorylated lysosomal enzymes from the Golgi complex and the cell surface to lysosomes. Lysosomal enzymes bearing phosphomannosyl residues bind specifically to mannose-6- phosphate receptors in the Golgi apparatus, and the resulting receptor–ligand complex is transported to an acidic prelyosomal compartment where the low pH mediates the dissociation of the complex. This receptor also binds IGF2. Acts as a positive regulator of T-cell coactivation, by binding DPP4; CD molecules
IRS2	9606.ENSP00000365016	Insulin receptor substrate 2; May mediate the control of various cellular processes by insulin; Pleckstrin homology domain containing
JPH1	9606.ENSP00000344488	Junctophilin-1; Junctophilins contribute to the formation of junctional membrane complexes (JMCs), which link the plasma membrane with the endoplasmic or sarcoplasmic reticulum in excitable cells. Provides a structural foundation for functional cross-talk between the cell surface and intracellular calcium release channels. JPH1 contributes to the construction of the skeletal muscle triad by linking the t-tubule (transverse-tubule) and SR (sarcoplasmic reticulum) membranes
KIAA1524	9606.ENSP00000295746	Cellular inhibitor of pp2a; Protein CIP2A; Oncoprotein that inhibits PP2A and stabilizes MYC in human malignancies. Promotes anchorage-independent cell growth and tumor formation
KIDINS220	9606.ENSP00000256707	Ankyrin repeat-rich membrane spanning protein; Kinase D-interacting substrate of 220 kDa; Promotes a prolonged MAP-kinase signaling by neurotrophins through activation of a Rap1-dependent mechanism. Provides a docking site for the CRKL-C3G complex, resulting in Rap1-dependent sustained ERK activation. May play an important role in regulating postsynaptic signal transduction through the syntrophin-mediated localization of receptor tyrosine kinases such as EPHA4. In cooperation with SNTA1 can enhance EPHA4-induced JAK/STAT activation.
LBR	9606.ENSP00000339883	Delta14-sterol reductase (lamin-B receptor); Lamin-B receptor; Anchors the lamina and the heterochromatin to the inner nuclear membrane; Tudor domain containing
LLGL1	9606.ENSP00000321537	LLGL1, scribble cell polarity complex component; Lethal(2) giant larvae protein homolog 1; Cortical cytoskeleton protein found in a complex involved in maintaining cell polarity and epithelial integrity. Involved in the regulation of mitotic spindle orientation, proliferation, differentiation, and tissue organization of neuroepithelial cells. Involved in axonogenesis through RAB10 activation, thereby regulating vesicular membrane trafficking toward the axonal plasma membrane
LRBA	9606.ENSP00000349629	Lipopolysaccharide-responsive and beige-like anchor protein; May be involved in coupling signal transduction and vesicle trafficking to enable polarized secretion and/or membrane deposition of immune effector molecules; Armadillo-like helical domain containing
LRRC7	9606.ENSP00000035383	Leucine-rich repeat-containing protein 7; Required for normal synaptic spine architecture and function. Necessary for DISC1 and GRM5 localization to postsynaptic density complexes and for both N-methyl d-aspartate receptor-dependent and metabotropic glutamate receptor-dependent long-term depression; Belongs to the LAP (LRR and PDZ) protein family
LRRC8B	9606.ENSP00000332674	Volume-regulated anion channel subunit LRRC8B; Non-essential component of the volume-regulated anion channel (VRAC, also named VSOAC channel), an anion channel required to maintain a constant cell volume in response to extracellular or intracellular osmotic changes. The VRAC channel conducts iodide better than chloride and may also conduct organic osmolytes like taurine. Channel activity requires LRRC8A plus at least one other family member (LRRC8B, LRRC8C, LRRC8D or LRRC8E); channel characteristics depend on the precise subunit composition
LRRC8C	9606.ENSP00000359483	Volume-regulated anion channel subunit LRRC8C; Non-essential component of the volume-regulated anion channel (VRAC, also named VSOAC channel), an anion channel required to maintain a constant cell volume in response to extracellular or intracellular osmotic changes. The VRAC channel conducts iodide better than chloride and may also conduct organic osmolytes like taurine. Channel activity requires LRRC8A plus at least one other family member (LRRC8B, LRRC8C, LRRC8D or LRRC8E); channel characteristics depend on the precise subunit composition
LRRC8D	9606.ENSP00000338887	Volume-regulated anion channel subunit LRRC8D; Non-essential component of the volume-regulated anion channel (VRAC, also named VSOAC channel), an anion channel required to maintain a constant cell volume in response to extracellular or intracellular osmotic changes. The VRAC channel conducts iodide better than chloride and may also conduct organic osmolytes like taurine. Channel activity requires LRRC8A plus at least one other family member (LRRC8B, LRRC8C, LRRC8D or LRRC8E); channel characteristics depend on the precise subunit composition
LSG1	9606.ENSP00000265245	Large subunit GTPase 1 homolog; GTPase required for the XPO1/CRM1-mediated nuclear export of the 60S ribosomal subunit. Probably acts by mediating the release of NMD3 from the 60S ribosomal subunit after export into the cytoplasm
LSR	9606.ENSP00000480821	Lipolysis-stimulated lipoprotein receptor; Probable role in the clearance of triglyceride-rich lipoprotein from blood. Binds chylomicrons, LDL and VLDL in the presence of free fatty acids and allows their subsequent uptake in the cells (By similarity); Belongs to the immunoglobulin superfamily. LISCH7 family
MACF1	9606.ENSP00000354573	Microtubule-actin cross-linking factor 1, isoforms 1/2/3/5; Isoform 2: F-actin-binding protein which plays a role in cross-linking actin to other cytoskeletal proteins and also binds to microtubules. Plays an important role in ERBB2-dependent stabilization of microtubules at the cell cortex. Acts as a positive regulator of the Wnt receptor signaling pathway and is involved in the translocation of AXIN1 and its associated complex (composed of APC, CTNNB1, and GSK3B) from the cytoplasm to the cell membrane (By similarity).
MLLT4	9606.ENSP00000375960	Afadin; Belongs to an adhesion system, probably together with the E-cadherin–catenin system, which plays a role in the organization of homotypic, interneuronal, and heterotypic cell–cell adherens junctions (AJs). Nectin- and actin-filament-binding protein that connects nectin to the actin cytoskeleton
MYO6	9606.ENSP00000358994	Unconventional myosin-VI; Myosins are actin-based motor molecules with ATPase activity. Unconventional myosins serve in intracellular movements. Myosin 6 is a reverse-direction motor protein that moves towards the minus-end of actin filaments. Has a slow rate of actin-activated ADP release due to weak ATP binding. Functions in a variety of intracellular processes such as vesicular membrane trafficking and cell migration. Required for the structural integrity of the Golgi apparatus via the p53-dependent pro-survival pathway.
NDC1	9606.ENSP00000360483	NDC1 transmembrane nucleoporin; Nucleoporin NDC1; Component of the nuclear pore complex (NPC), which plays a key role in de novo assembly and insertion of NPC in the nuclear envelope. Required for NPC and nuclear envelope assembly, possibly by forming a link between the nuclear envelope membrane and soluble nucleoporins, thereby anchoring the NPC in the membrane
NDRG1	9606.ENSP00000404854	N-myc downstream regulated 1; Protein NDRG1; Stress-responsive protein involved in hormone responses, cell growth, and differentiation. Acts as a tumor suppressor in many cell types. Necessary but not sufficient for p53/TP53-mediated caspase activation and apoptosis. Has a role in cell trafficking, notably of the Schwann cell, and is necessary for the maintenance and development of the peripheral nerve myelin sheath. Required for vesicular recycling of CDH1 and TF. May also function in lipid trafficking. Protects cells from spindle disruption damage.
NECTIN2	9606.ENSP00000252483	Nectin-2; Modulator of T-cell signaling. Can be either a costimulator of T-cell function or a coinhibitor depending on the receptor it binds to. Upon binding to CD226, stimulates T-cell proliferation and cytokine production, including that of IL2, IL5, IL10, IL13, and IFNG. Upon interaction with PVRIG, inhibits T-cell proliferation. These interactions are competitive. Probable cell adhesion protein; Belongs to the nectin family
NSDHL	9606.ENSP00000359297	Sterol-4-alpha-carboxylate 3-dehydrogenase, decarboxylating; Involved in the sequential removal of two C-4 methyl groups in post-squalene cholesterol biosynthesis; Short chain dehydrogenase/reductase superfamily
NUMB	9606.ENSP00000451300	Numb, endocytic adaptor protein; Protein numb homolog; Plays a role in the process of neurogenesis. Required throughout embryonic neurogenesis to maintain neural progenitor cells, also called radial glial cells (RGCs), by allowing their daughter cells to choose progenitor over neuronal cell fate. Not required for the proliferation of neural progenitor cells before the onset of neurogenesis. Also involved postnatally in the subventricular zone (SVZ) neurogenesis by regulating SVZ neuroblasts survival and ependymal wall integrity. May also mediate local repair of brain ventricular wall damage
NUMBL	9606.ENSP00000252891	Numb-like protein; Plays a role in the process of neurogenesis. Required throughout embryonic neurogenesis to maintain neural progenitor cells, also called radial glial cells (RGCs), by allowing their daughter cells to choose progenitor over neuronal cell fate. Not required for the proliferation of neural progenitor cells before the onset of embryonic neurogenesis. Also required postnatally in the subventricular zone (SVZ) neurogenesis by regulating SVZ neuroblasts survival and ependymal wall integrity. Negative regulator of NF-kappa-B signaling pathway. The inhibition of NF-kappa-B a […]
OCLN	9606.ENSP00000347379	Occludin; May play a role in the formation and regulation of the tight junction (TJ) paracellular permeability barrier. It is able to induce adhesion when expressed in cells lacking tight junctions; Protein phosphatase 1 regulatory subunits
OSBPL8	9606.ENSP00000261183	Oxysterol-binding protein-related protein 8; Lipid transporter involved in lipid countertransport between the endoplasmic reticulum and the plasma membrane: specifically exchanges phosphatidylserine with phosphatidylinositol 4-phosphate (PI4P), delivering phosphatidylserine to the plasma membrane in exchange for PI4P, which is degraded by the SAC1/SACM1L phosphatase in the endoplasmic reticulum. Binds phosphatidylserine and PI4P in a mutually exclusive manner. Binds oxysterol, 25-hydroxycholesterol, and cholesterol; Belongs to the OSBP family
PAK4	9606.ENSP00000469413	Serine/threonine-protein kinase PAK 4; Serine/threonine protein kinase that plays a role in a variety of different signaling pathways, including cytoskeleton regulation, cell migration, growth, proliferation, or cell survival. Activation by various effectors, including growth factor receptors or active CDC42 and RAC1, results in a conformational change and a subsequent autophosphorylation on several serine and/or threonine residues. Phosphorylates and inactivates the protein phosphatase SSH1, leading to increased inhibitory phosphorylation of the actin-binding/depolymerizing factor cofilin
PEAK1	9606.ENSP00000452796	Pseudopodium-enriched atypical kinase 1; Tyrosine kinase that may play a role in cell spreading and migration on fibronectin. May directly or indirectly affect phosphorylation levels of cytoskeleton-associated proteins MAPK1/ERK and PXN
PHACTR4	9606.ENSP00000362942	Phosphatase and actin regulator 4; Regulator of protein phosphatase 1 (PP1) required for neural tube and optic fissure closure, and enteric neural crest cell (ENCCs) migration during development. Acts as an activator of PP1 by interacting with PPP1CA and preventing phosphorylation of PPP1CA at ‘Thr-320’. During neural tube closure, localizes to the ventral neural tube and activates PP1, leading to down-regulate cell proliferation within cranial neural tissue and the neural retina. Also acts as a regulator of migration of enteric neural crest cells (ENCCs) by activating PP1
PKN2	9606.ENSP00000359552	Serine/threonine-protein kinase N2; PKC-related serine/threonine-protein kinase and Rho/Rac effector protein that participates in specific signal transduction responses in the cell. Plays a role in the regulation of cell cycle progression, actin cytoskeleton assembly, cell migration, cell adhesion, tumor cell invasion, and transcription activation signaling processes. Phosphorylates CTTN in hyaluronan-induced astrocytes and hence decreases CTTN's ability to associate with filamentous actin. Phosphorylates HDAC5, therefore, lead to impaired HDAC5 import.
PLEKHA5	9606.ENSP00000404296	Pleckstrin homology domain-containing family a member 5; Pleckstrin homology domain-containing A5
PPFIBP1	9606.ENSP00000314724	Ppfia binding protein 1, Liprin-beta-1; May regulate the disassembly of focal adhesions. Did not bind receptor-like tyrosine phosphatases type 2 A; Sterile alpha motif domain containing
PREB	9606.ENSP00000260643	Prolactin regulatory element-binding protein; Guanine nucleotide exchange factor that specifically activates the small GTPase SAR1B. Mediates the recruitment of SAR1B and other COPII coat components to endoplasmic reticulum membranes and is therefore required for the formation of COPII transport vesicles from the ER; WD repeat domain-containing
PSD3	9606.ENSP00000324127	PH and SEC7 domain-containing protein 3; Guanine nucleotide exchange factor for ARF6; Pleckstrin homology domain containing
PTPN1	9606.ENSP00000360683	Tyrosine-protein phosphatase non-receptor type 1; Tyrosine-protein phosphatase which acts as a regulator of endoplasmic reticulum unfolded protein response. Mediates dephosphorylation of EIF2AK3/PERK; inactivating the protein kinase activity of EIF2AK3/PERK. May play an important role in CKII- and p60c-src-induced signal transduction cascades. May regulate the EFNA5-EPHA3 signaling pathway, which modulates cell reorganization and cell-cell repulsion. May also regulate the hepatocyte growth factor receptor signaling pathway through the dephosphorylation of MET
PTPN13	9606.ENSP00000394794	Tyrosine-protein phosphatase non-receptor type 13; Tyrosine phosphatase which regulates negatively FAS-induced apoptosis and NGFR-mediated pro-apoptotic signaling. May regulate phosphoinositide 3-kinase (PI3K) signaling through dephosphorylation of PIK3R2; FERM domain containing
RAB23	9606.ENSP00000417610	RAB23, member RAS oncogene family; Ras-related protein Rab-23; The small GTPases Rab are key regulators of intracellular membrane trafficking, from the formation of transport vesicles to their fusion with membranes. Rabs cycle between an inactive GDP-bound form and an active GTP-bound form that is able to recruit to membranes different sets of downstream effectors directly responsible for vesicle formation, movement, tethering, and fusion. Together with SUFU, it prevents nuclear import of GLI1, and thereby inhibits GLI1 transcription factor activity.
RAI14	9606.ENSP00000427123	Retinoic acid-induced 14; Ankycorbin; Plays a role in actin regulation at the ectoplasmic specialization, a type of cell junction specific to the testis. Important for the establishment of sperm polarity and normal spermatid adhesion. May also promote the integrity of Sertoli cell tight junctions at the blood-testis barrier; Ankyrin repeat domain-containing
RAPGEF6	9606.ENSP00000296859	Rap guanine nucleotide exchange factor 6; Guanine nucleotide exchange factor (GEF) for Rap1A, Rap2A, and M-Ras GTPases. Does not interact with cAMP; PDZ domain-containing
RASAL2	9606.ENSP00000356621	Ras GTPase-activating protein nGAP; Inhibitory regulator of the Ras-cyclic AMP pathway; C2 and RasGAP domain containing
RICTOR	9606.ENSP00000296782	Rapamycin-insensitive companion of mTOR; Subunit of mTORC2, which regulates cell growth and survival in response to hormonal signals. mTORC2 is activated by growth factors but, in contrast to mTORC1, seems to be nutrient-insensitive. mTORC2 seems to function upstream of Rho GTPases to regulate the actin cytoskeleton, probably by activating one or more Rho-type guanine nucleotide exchange factors. mTORC2 promotes the serum-induced formation of stress fibers or F-actin. mTORC2 plays a critical role in AKT1 ‘
ROR2	9606.ENSP00000364860	Tyrosine-protein kinase transmembrane receptor ROR2; Tyrosine-protein kinase receptor which may be involved in the early formation of the chondrocytes. It seems to be required for cartilage and growth plate development (By similarity). Phosphorylates YWHAB, leading to induction of osteogenesis and bone formation. In contrast, has also been shown to have very little tyrosine kinase activity in vitro. May act as a receptor for wnt ligand WNT5A, which may result in the inhibition of WNT3A-mediated signaling; I-set domain containing
RUVBL1	9606.ENSP00000318297	RuvB-like 1; May be able to bind plasminogen at the cell surface and enhance plasminogen activation; AAA ATPases
SCRIB	9606.ENSP00000349486	Protein scribble homolog; Scaffold protein involved in different aspects of polarized cell differentiation regulating epithelial and neuronal morphogenesis. Most probably functions in the establishment of apico-basal cell polarity. May function in cell proliferation regulating progression from G1 to S phase and as a positive regulator of apoptosis for instance during acinar morphogenesis of the mammary epithelium. May also function in cell migration and adhesion and hence regulate cell invasion through MAPK signaling. May play a role in exocytosis and in the targeting synaptic vesicle
SEC24B	9606.ENSP00000428564	Protein transport protein Sec24B; Component of the coat protein complex II (COPII) which promotes the formation of transport vesicles from the endoplasmic reticulum (ER). The coat has two main functions, the physical deformation of the endoplasmic reticulum membrane into vesicles and the selection of cargo molecules for their transport to the Golgi complex. Plays a central role in cargo selection within the COPII complex and, together with SEC24A, may have a different specificity compared to SEC24C and SEC24D. May package preferentially cargos with cytoplasmic DxE or LxxLE motifs
SEPT9	9606.ENSP00000391249	Septin-9; Filament-forming cytoskeletal GTPase (By similarity). May play a role in cytokinesis (Potential). May play a role in the internalization of 2 intracellular microbial pathogens. Belongs to the TRAFAC class TrmE-Era-EngA-EngB-Septin-like GTPase superfamily. Septin GTPase family
SH3D19	9606.ENSP00000302913	SH3 domain-containing protein 19; May play a role in regulating A disintegrin and metalloproteases (ADAMs) in the signaling of EGFR-ligand shedding. May be involved in suppression of Ras-induced cellular transformation and Ras-mediated activation of ELK1. Plays a role in the regulation of cell morphology and cytoskeletal organization
SLC26A6	9606.ENSP00000378920	Solute carrier family 26 member 6; Apical membrane anion-exchanger with a wide epithelial distribution that plays a role as a component of the pH buffering system for maintaining acid–base homeostasis. Acts as a versatile DIDS-sensitive inorganic and organic anion transporter that mediates the uptake of monovalent anions like chloride, bicarbonate, formate, and hydroxyl ion and divalent anions like sulfate and oxalate. Function in multiple exchange modes involving pairs of these anions
SLC38A1	9606.ENSP00000449756	Sodium-coupled neutral amino acid transporter 1; Functions as a sodium-dependent amino acid transporter. Mediates the saturable, pH-sensitive, and electrogenic cotransport of glutamine and sodium ions with a stoichiometry of 1:1. May also transport small zwitterionic and aliphatic amino acids with a lower affinity. May supply glutamatergic and GABAergic neurons with glutamine which is required for the synthesis of the neurotransmitters glutamate and GABA; Solute carriers
SLC39A10	9606.ENSP00000386766	Solute carrier family 39 (zinc transporter), member 10; Zinc transporter ZIP10; May act as a zinc-influx transporter; Belongs to the ZIP transporter (TC 2.A.5) family
SLC3A2	9606.ENSP00000367123	4F2 cell-surface antigen heavy chain; Required for the function of light chain amino-acid transporters. Involved in sodium-independent, high-affinity transport of large neutral amino acids such as phenylalanine, tyrosine, leucine, arginine, and tryptophan. Involved in guiding and targeting LAT1 and LAT2 to the plasma membrane. When associated with SLC7A6 or SLC7A7 acts as an arginine/glutamine exchanger, following an antiport mechanism for amino acid transport, influencing arginine release in exchange for extracellular amino acids.
SLC6A15	9606.ENSP00000266682	Sodium-dependent neutral amino acid transporter B(0)AT2; Functions as a sodium-dependent neutral amino acid transporter. Exhibits preference for the branched-chain amino acids, particularly leucine, valine, isoleucine, and methionine. Mediates the saturable, pH-sensitive, and electrogenic cotransport of proline and sodium ions with a stoichiometry of 1:1. May have a role as a transporter for neurotransmitter precursors into neurons. In contrast to other members of the neurotransmitter transporter family, does not appear to be chloride-dependent; Solute carriers
SNAP23	9606.ENSP00000249647	Synaptosomal-associated protein 23; Essential component of the high-affinity receptor for the general membrane fusion machinery and an important regulator of transport vesicle docking and fusion; Belongs to the SNAP-25 family
SNX1	9606.ENSP00000261889	Sorting nexin-1; Involved in several stages of intracellular trafficking. Interacts with membranes containing phosphatidylinositol 3- phosphate (PtdIns(3P)) or phosphatidylinositol 3,5-bisphosphate (PtdIns(3,5)P2). Acts in part as a component of the retromer membrane-deforming SNX-BAR subcomplex. The SNX-BAR retromer mediates retrograde transport of cargo proteins from endosomes to the trans-Golgi network (TGN) and is involved in endosome-to-plasma membrane transport for cargo protein recycling. The SNX-BAR subcomplex functions to deform the donor membrane into a tubular profile
SPTAN1	9606.ENSP00000361824	Spectrin alpha chain, non-erythrocytic 1; Fodrin, which seems to be involved in secretion, interacts with calmodulin in a calcium-dependent manner and is thus a candidate for the calcium-dependent movement of the cytoskeleton at the membrane; EF-hand domain containing
SRPRA	9606.ENSP00000328023	Signal recognition particle receptor subunit alpha; Component of the SRP (signal recognition particle) receptor. Ensures, in conjunction with the signal recognition particle, the correct targeting of the nascent secretory proteins to the endoplasmic reticulum membrane system
STAMBP	9606.ENSP00000377633	STAM-binding protein; Zinc metalloprotease that specifically cleaves ‘Lys-63’- linked polyubiquitin chains. Does not cleave ‘Lys-48’-linked polyubiquitin chains (By similarity). Plays a role in signal transduction for cell growth and MYC induction mediated by IL-2 and GM-CSF. Potentiates BMP (bone morphogenetic protein) signaling by antagonizing the inhibitory action of SMAD6 and SMAD7. Has a key role in the regulation of cell surface receptor-mediated endocytosis and ubiquitin-dependent sorting of receptors to lysosomes. Endosomal localization of STAMBP is required for efficient EGFR degradation
STEAP3	9606.ENSP00000376822	Metalloreductase STEAP3; Endosomal ferrireductase required for efficient transferrin-dependent iron uptake in erythroid cells. Participates in erythroid iron homeostasis by reducing Fe(3+) to Fe(2+). Can also reduce of Cu(2+) to Cu(1+), suggesting that it participates in copper homeostasis. Uses NADP(+) as acceptor. May play a role downstream of p53/TP53 to interface apoptosis and cell cycle progression. Indirectly involved in exosome secretion by facilitating the secretion of proteins such as TCTP; STEAP family
STIM1	9606.ENSP00000478059	Stromal interaction molecule 1; Plays a role in mediating store-operated Ca(2+) entry (SOCE), a Ca(2+) influx following the depletion of intracellular Ca(2+) stores. Acts as Ca(2+) sensor in the endoplasmic reticulum via its EF-hand domain. Upon Ca(2+) depletion, it translocates from the endoplasmic reticulum to the plasma membrane where it activates the Ca(2+) release-activated Ca(2+) (CRAC) channel subunit ORAI1. Involved in enamel formation. Activated following interaction with STIMATE, leading to promote STIM1 conformational switch; Sterile alpha motif domain containing
SUGT1	9606.ENSP00000367208	SGT1 homolog, MIS12 kinetochore complex assembly cochaperone; Protein SGT1 homolog; May play a role in ubiquitination and subsequent proteasomal degradation of target proteins
TACC1	9606.ENSP00000321703	Transforming acidic coiled-coil-containing protein 1; Likely involved in the processes that promote cell division prior to the formation of differentiated tissues
TMEM57	9606.ENSP00000363463	Macoilin 1; Plays a role in the regulation of neuronal activity
TMPO	9606.ENSP00000266732	Lamina-associated polypeptide 2, isoform alpha; May be involved in the structural organization of the nucleus and in the post-mitotic nuclear assembly. Plays an important role, together with LMNA, in the nuclear anchorage of RB1; Belongs to the LEM family
TOR1AIP1	9606.ENSP00000435365	Torsin-1A-interacting protein 1; Required for nuclear membrane integrity. Induces TOR1A and TOR1B ATPase activity and is required for their location on the nuclear membrane. Binds to A- and B-type lamins. Possible role in membrane attachment and assembly of the nuclear lamina
TTK	9606.ENSP00000358813	Serine/threonine-protein kinase ttk/mps1; Dual specificity protein kinase TTK; Phosphorylates proteins on serine, threonine, and tyrosine. Probably associated with cell proliferation. Essential for chromosome alignment by enhancing AURKB activity (via direct CDCA8 phosphorylation) at the centromere, and for the mitotic checkpoint
UBE2J1	9606.ENSP00000451261	Ubiquitin-conjugating enzyme E2 J1; Catalyzes the covalent attachment of ubiquitin to other proteins. Functions in the selective degradation of misfolded membrane proteins from the endoplasmic reticulum (ERAD); Belongs to the ubiquitin-conjugating enzyme family
UBIAD1	9606.ENSP00000366006	UbiA prenyltransferase domain-containing protein 1; Prenyltransferase that mediates the formation of menaquinone-4 (MK-4) and coenzyme Q10. MK-4 is a vitamin K2 isoform present at high concentrations in the brain, kidney, and pancreas and is required for endothelial cell development. Mediates the conversion of phylloquinone (PK) into MK-4, probably by cleaving the side chain of phylloquinone (PK) to release 2- methyl-1,4-naphthoquinone (menadione; K3) and then prenylating it with geranylgeranyl pyrophosphate (GGPP) to form MK-4.
USP6NL	9606.ENSP00000277575	USP6 N-terminal-like protein; Acts as a GTPase-activating protein for RAB5A and RAB43. Involved in receptor trafficking. In complex with EPS8, it inhibits the internalization of EGFR. Involved in retrograde transport from the endocytic pathway to the Golgi apparatus. Involved in the transport of Shiga toxin from early and recycling endosomes to the trans-Golgi network. Required for the structural integrity of the Golgi complex
UTRN	9606.ENSP00000356515	Utrophin; May play a role in anchoring the cytoskeleton to the plasma membrane; Zinc fingers ZZ-type
VANGL1	9606.ENSP00000347672	VANGL planar cell polarity protein 1
VAPB	9606.ENSP00000417175	Vesicle-associated membrane protein-associated protein B/C; Participates in the endoplasmic reticulum unfolded protein response (UPR) by inducing ERN1/IRE1 activity. Involved in cellular calcium homeostasis regulation
VRK2	9606.ENSP00000408002	Serine/threonine-protein kinase VRK2; Serine/threonine kinase that regulates several signal transduction pathways. Isoform 1 modulates the stress response to hypoxia and cytokines, such as interleukin-1 beta (IL1B), and this is dependent on its interaction with MAPK8IP1, which assembles mitogen-activated protein kinase (MAPK) complexes. Inhibition of signal transmission mediated by the assembly of MAPK8IP1-MAPK complexes reduces JNK phosphorylation and JUN-dependent transcription. Phosphorylates ‘Thr-18’ of p53/TP53, histone H3, and may also phosphorylate MAPK8IP1
WDR45B	9606.ENSP00000376139	Wd repeat domain phosphoinositide-interacting protein 3; Component of the autophagy machinery that controls the major intracellular degradation process by which cytoplasmic materials are packaged into autophagosomes and delivered to lysosomes for degradation. Binds phosphatidylinositol 3-phosphate (PtdIns3P) forming on membranes of the endoplasmic reticulum upon activation of the upstream ULK1 and PI3 kinases and is recruited at phagophore assembly sites where it regulates the elongation of nascent phagophores downstream of WIPI2
WIPI2	9606.ENSP00000288828	WD repeat domain phosphoinositide-interacting protein 2; Early component of the autophagy machinery being involved in the formation of preautophagosomal structures and their maturation into mature phagosomes in response to phosphatidylinositol 3-phosphate (PtdIns3P). May bind PtdIns3P
WWOX	9606.ENSP00000457230	WW domain-containing oxidoreductase; Putative oxidoreductase. Acts as a tumor suppressor and plays a role in apoptosis. Required for normal bone development (By similarity). May function synergistically with p53/TP53 to control genotoxic stress-induced cell death. Plays a role in TGFB1 signaling and TGFB1-mediated cell death. May also play a role in tumor necrosis factor (TNF)-mediated cell death. Inhibits Wnt signaling, probably by sequestering DVL2 in the cytoplasm; Short chain dehydrogenase/reductase superfamily
YKT6	9606.ENSP00000223369	Synaptobrevin homolog YKT6; Vesicular soluble NSF attachment protein receptor (v-SNARE) mediating vesicle docking and fusion to a specific acceptor cellular compartment. Functions in endoplasmic reticulum to Golgi transport as part of a SNARE complex composed of GOSR1, GOSR2, and STX5. Functions in early/recycling endosome to TGN transport as part of a SNARE complex composed of BET1L, GOSR1, and STX5. Has a S-palmitoyl transferase activity; SNAREs
ZC3HAV1	9606.ENSP00000242351	Zinc finger CCCH-type antiviral protein 1; Antiviral protein that inhibits the replication of viruses by recruiting the cellular RNA degradation machineries to degrade the viral mRNAs. Binds to a ZAP-responsive element (ZRE) present in the target viral mRNA, recruits cellular poly(A)- specific ribonuclease PARN to remove the poly(A) tail, and the 3’- 5’ exoribonuclease complex exosome to degrade the RNA body from the 3’-end. It also recruits the decapping complex DCP1-DCP2 through RNA helicase p72 (DDX17) to remove the cap structure of the viral mRNA to initiate its degradation
ZDHHC5	9606.ENSP00000287169	Palmitoyltransferase ZDHHC5; Palmitoyl acyltransferase for the G-protein coupled receptor SSTR5. Also palmitoylates FLOT2 (By similarity); Zinc fingers DHHC-type
ZFYVE16	9606.ENSP00000337159	Mad, mothers against decapentaplegic interacting protein; Zinc finger FYVE domain-containing protein 16; May be involved in regulating membrane trafficking in the endosomal pathway. Overexpression induces endosome aggregation. Required to target TOM1 to endosomes; Protein phosphatase 1 regulatory subunits
ZFYVE9	9606.ENSP00000287727	Mad, mothers against decapentaplegic interacting protein; Zinc finger FYVE domain-containing protein 9; Early endosomal protein that functions to recruit SMAD2/SMAD3 to intracellular membranes and to the TGF-beta receptor. Plays a significant role in TGF-mediated signaling by regulating the subcellular location of SMAD2 and SMAD3 and modulating the transcriptional activity of the SMAD3/SMAD4 complex. Possibly associated with TGF-beta receptor internalization; Zinc fingers FYVE-type

Prepared using https://string-db.org/.

The original article has been updated.

