# Supplementary figures and images for: Interactomic exploration of LRRC8A in volume-regulated anion channels
Source: Cell Death Discov. 2024 Jun 22;10:299. doi: 10.1038/s41420-024-02032-0 (PMC11193767; doi:10.1038/s41420-024-02032-0)

## Slide 1
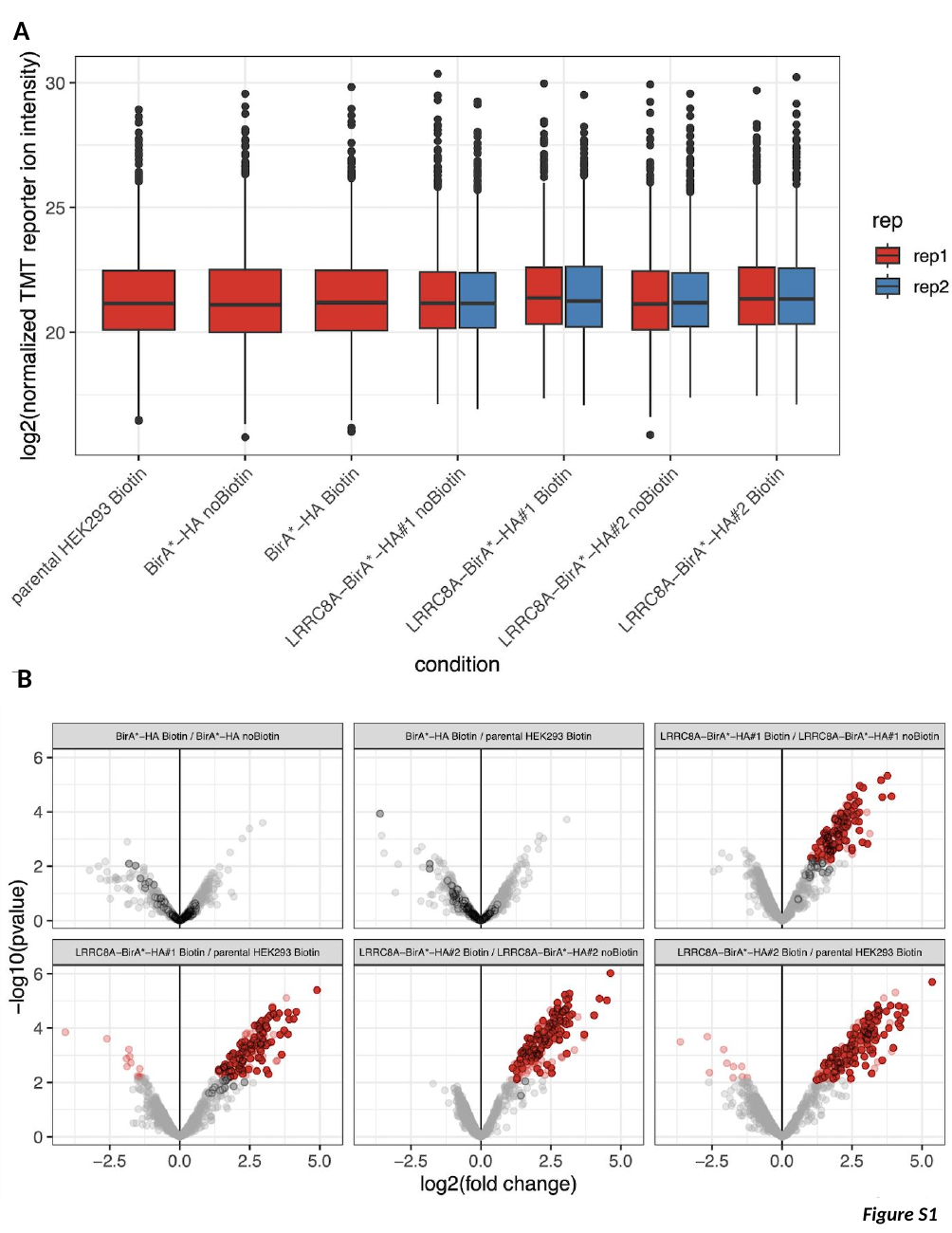

A
B
Figure S1

## Slide 2
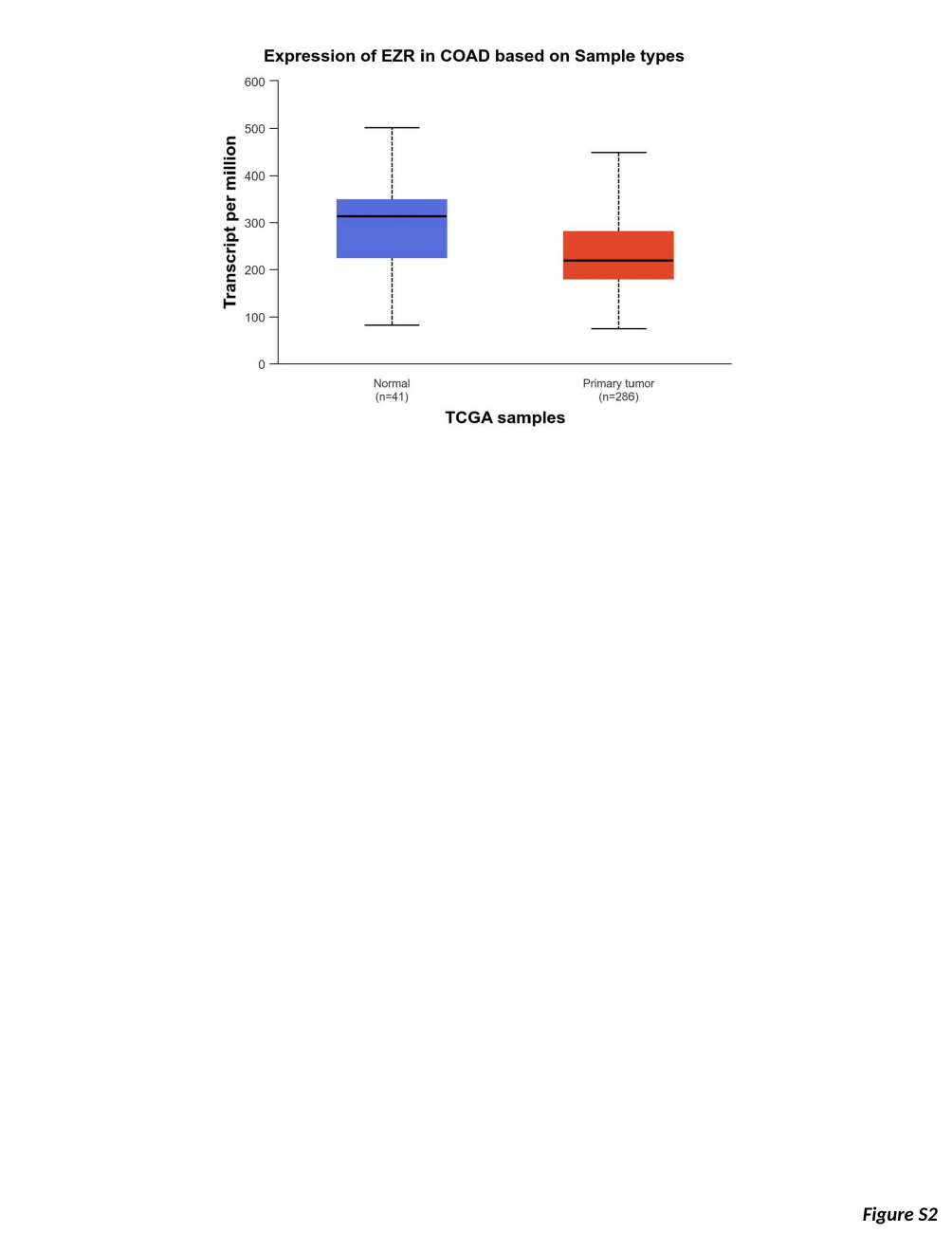

Figure S2

Supplement: Supplementary file 1 — Figure Supplementary S1 [file 41420_2024_2032_MOESM1_ESM.pptx]

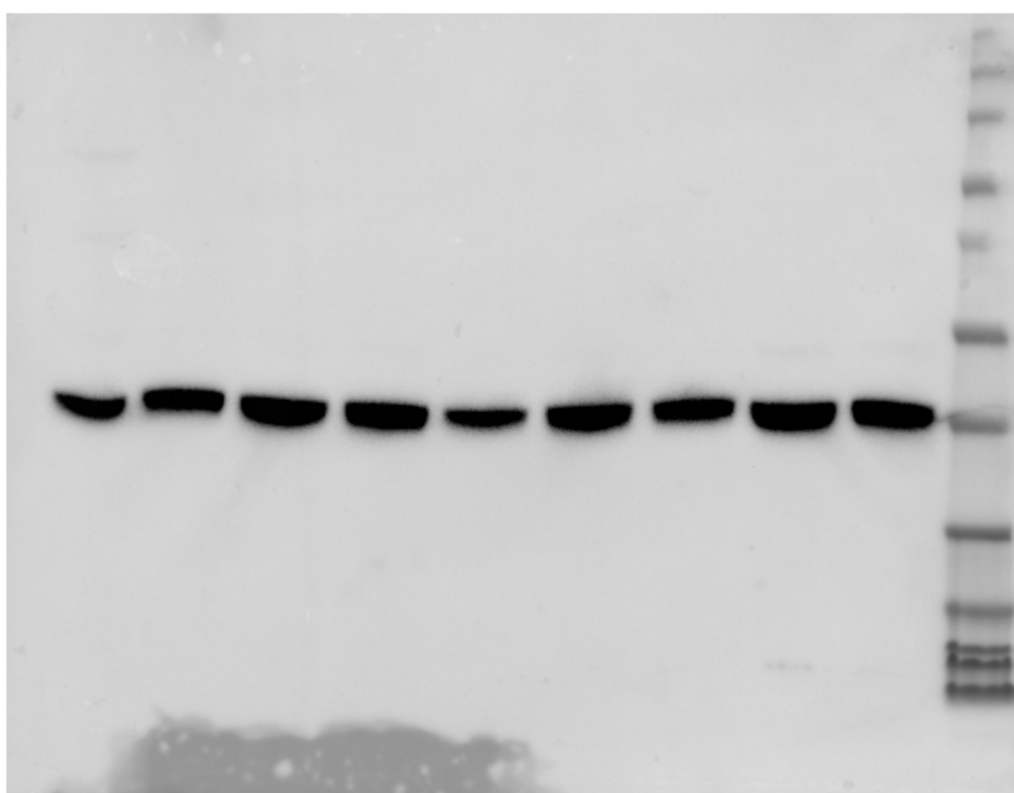

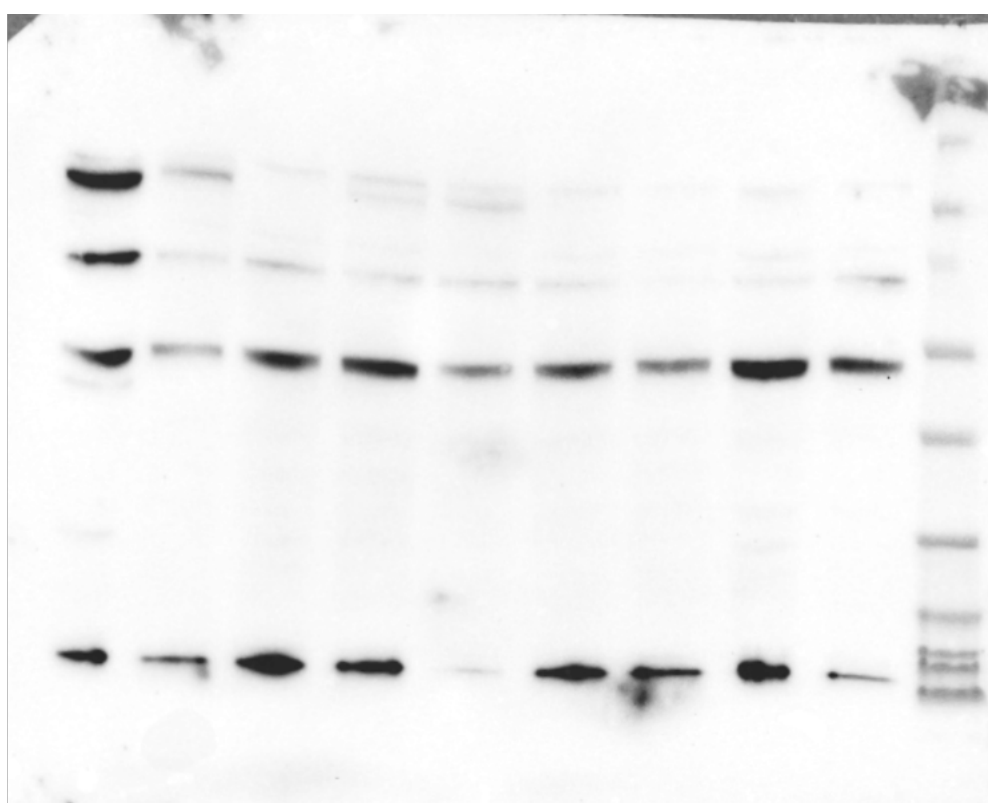

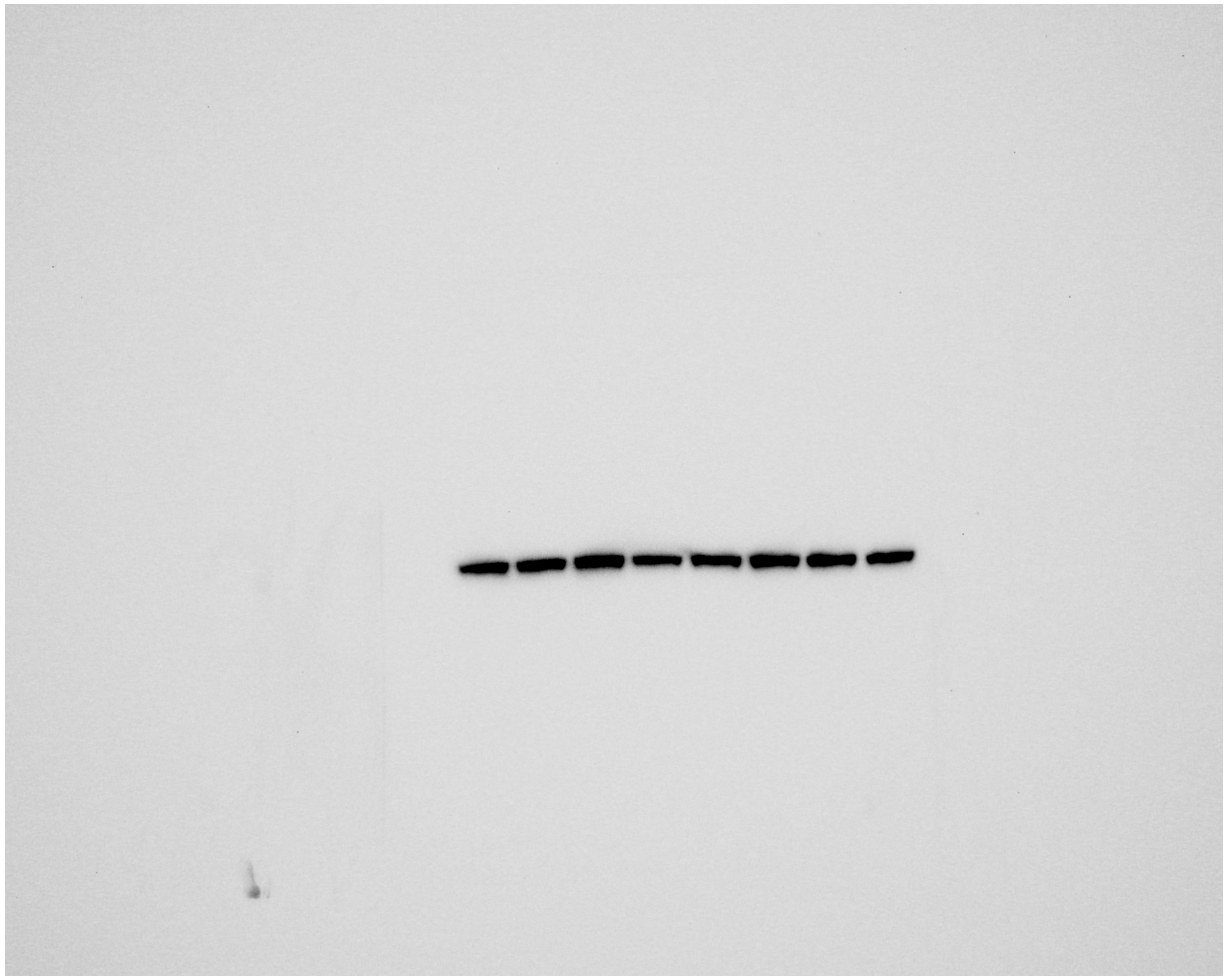

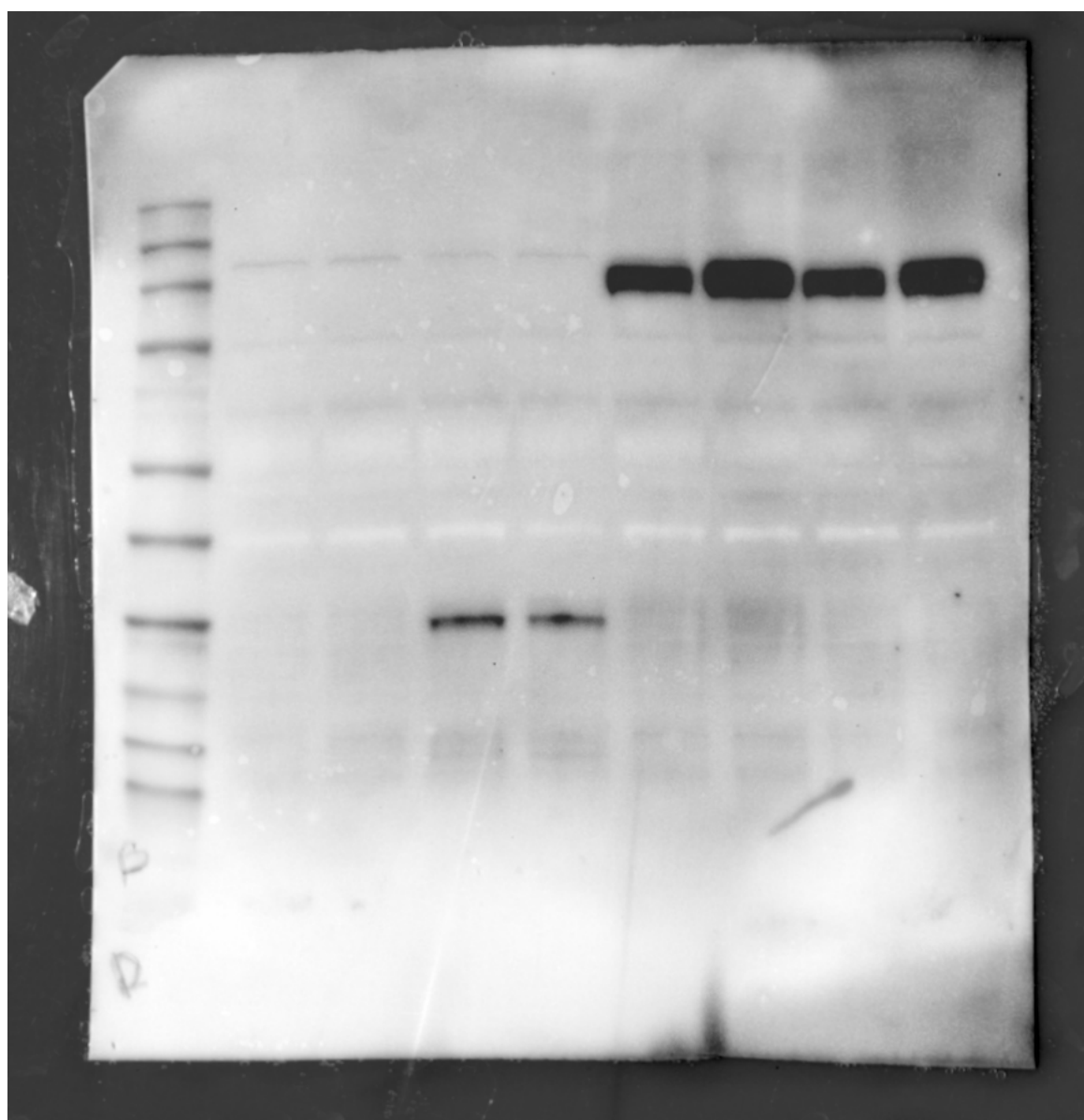

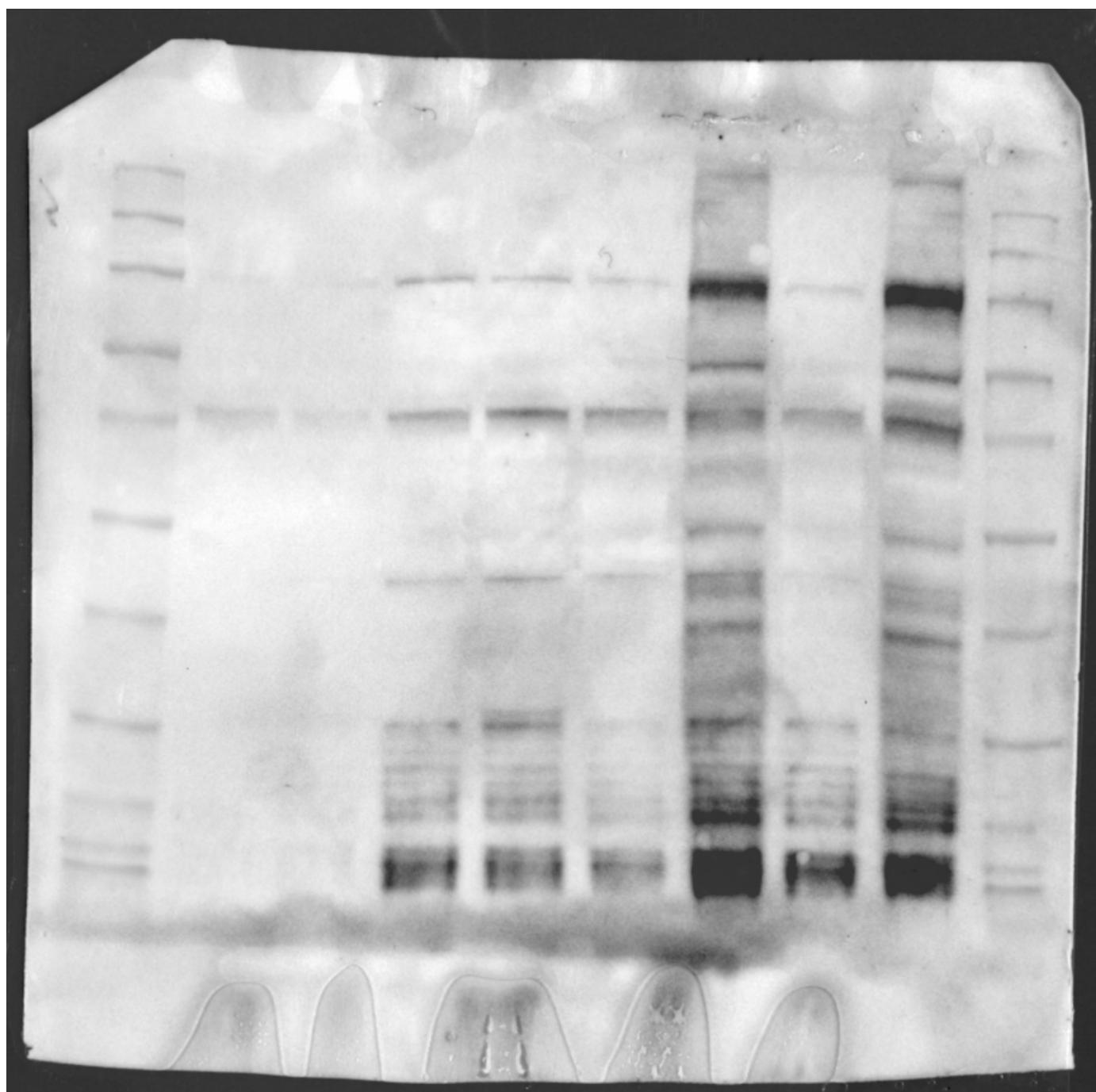

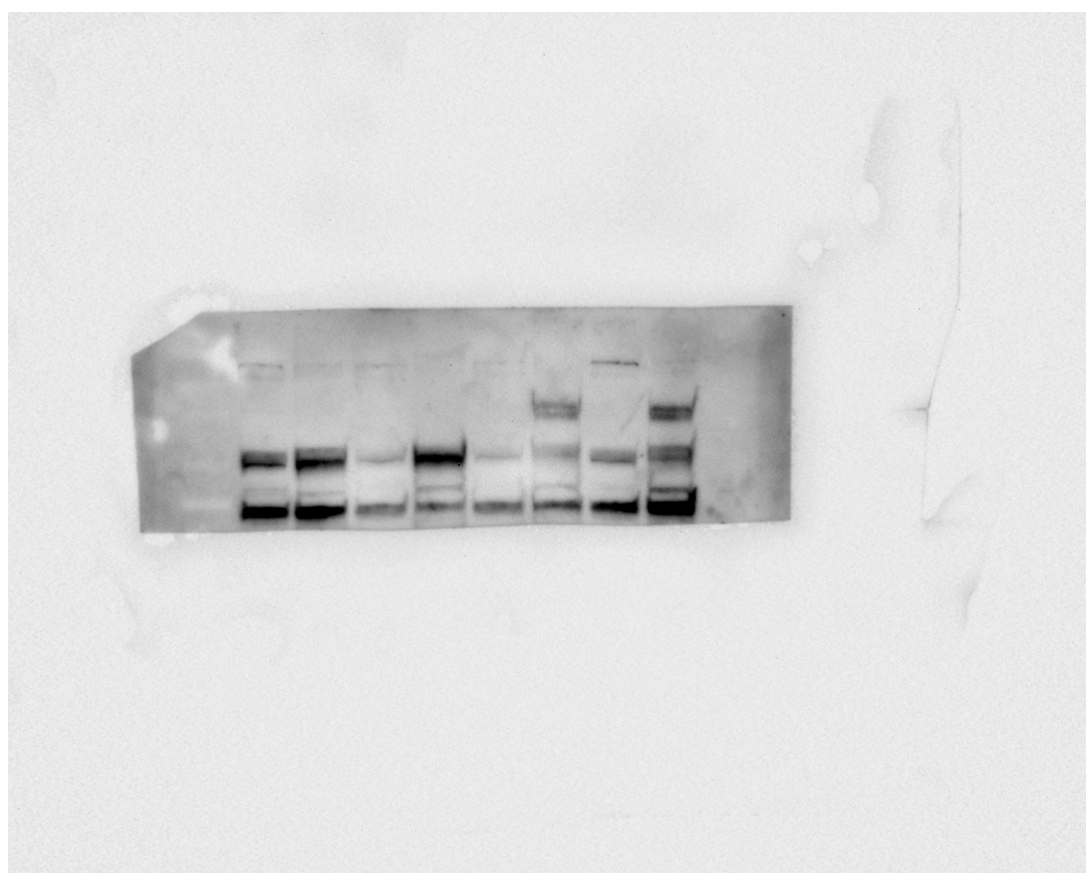

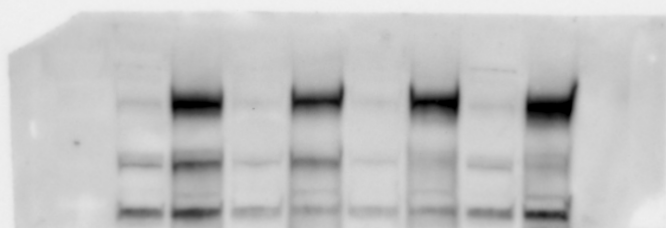

Supplement: Supplementary file 2 — Original blots (uncropped) [file 41420_2024_2032_MOESM2_ESM.pdf]
